# Supplementary material for: Calculation of the Vapour Pressure of Organic Molecules by Means of a Group-Additivity Method and Their Resultant Gibbs Free Energy and Entropy of Vaporization at 298.15 K
Source: Molecules. 2021 Feb 17;26(4):1045. doi: 10.3390/molecules26041045 (PMC7922249; doi:10.3390/molecules26041045)
Supplement: Supplementary file 1 [file molecules-26-01045-s001.zip › molecules-1089923-SM-proofed/S02. Experimental vs. calculated logVP Data Table.pdf]

Molecule name	logVP exp	logVP calc	logVP test	Deviation
(-)-Methyl jasmonate	-0.91	-1.18	-1.23	0.32
(-)-trans-Pinocarveol	1.34	1.2	1.24	0.1
(+)-trans-Myrtanol	0.37	1.01	1	-0.64
(2-Bromoethyl)benzene	1.51	1.43	1.42	0.09
(Chloromethyl)benzene	2.24	2.17	2.16	0.08
(E)-1,3,3,3-Tetrafluoropropene	5.7	5.69		0.01
(E)-1-Chloro-3,3,3-trifluoropropene	5.12	4.88	4.85	0.26
(Z)-1,3,3,3-Tetrafluoropropene	5.28	5.69		-0.41
1-(1,1-Difluoroethoxy)-1,1,2,2-tetrafluoroethane	4.01	4.31	4.35	-0.34
1-(2,2-Difluoroethoxy)-1,1,2,2,2-pentafluoroethane	4.66	4.62	4.61	0.04
1-(2,2-Difluoroethoxy)-1,1,2,2,3,3,3-heptafluoropropane	4.27	4.25	4.25	0.02
1-(2-Aminoethyl)-4-methylpiperazine	1.42	1.6	1.58	-0.16
1,1,1,2,2,3,3,4,4,5,5-Undecafluoro-5-methoxypentane	3.97	4.01	4.02	-0.05
1,1,1,2,2,3,3,4,4-Nonafluoro-4-propoxybutane	3.78	3.63	3.61	0.17
1,1,1,2,2,3,3-Heptafluoro-3-(2,2,2-trifluoroethoxy)propane	4.55	4.56	4.52	0.03
1,1,1,2,2,3,3-Heptafluoro-3-(2,2,3,3,3-pentafluoropropoxy)propane	4.21	4.19	4.17	0.04
1,1,1,2,2,3,3-Heptafluoro-3-(2,2,3,3-tetrafluoropropoxy)propane	3.97	3.88	3.88	0.09
1,1,1,2,2,3,3-Heptafluoro-3-(fluoromethoxy)propane	4.7	4.48		0.22
1,1,1,2,2,3,3-Heptafluoro-4-methoxybutane	4.21	4.26	4.25	-0.04
1,1,1,2,2-Pentafluoro-3-(1,1,2,2-tetrafluoroethoxy)propane	4.2	4.25	4.26	-0.06
1,1,1,2,2-Pentafluoro-3-methoxypropane	4.61	4.63	4.63	-0.02
1,1,1,2,2-Pentafluoro-3-pentafluoroethoxypropane	4.64	4.56	4.55	0.09
1,1,1,2,2-Pentafluoropropane	5.67	5.6	5.63	0.04
1,1,1,2,3,3,3-Heptafluoropropane	5.66	5.46	5.44	0.22
1,1,1,2,3,3,4,4-Octafluoro-4-methoxy-2-trifluoromethylbutane	3.99	4.03	4	-0.01
1,1,1,2,3,3-Hexafluoro-3-(2,2,2-trifluoroethoxy)propane	4.15	4.33	4.34	-0.19
1,1,1,2,3,3-Hexafluoro-3-(2,2,3,3,3-pentafluoropropoxy)propane	3.87	3.96	4	-0.13
1,1,1,2,3,3-Hexafluoro-3-(2,2,3,3-tetrafluoropropoxy)propane	3.5	3.65	3.68	-0.18
1,1,1,2,3,3-Hexafluoro-3-methoxypropane	4.5	4.52	4.52	-0.02
1,1,1,2,3,3-Hexafluoro-4-methoxybutane	3.91	3.8	3.71	0.2
1,1,1,2,3,3-Hexafluoro-4-trifluoromethoxybutane	4.32	4.62	4.69	-0.37
1,1,1,2,3,3-Hexafluoropropane	5.42	5.15	5.07	0.35
1,1,1,2,4,4,4-Heptafluoro-2-trifluoromethoxybutane	4.57	4.19	4.03	0.54
1,1,1,2,4,4-Hexafluoro-2-(trifluoromethoxy)butane	4.42	4.5	4.52	-0.1

1,1,1,2-Tetrachlorodifluoroethane	3.86	4.02	4.04	-0.18
1,1,1,2-Tetrachloroethane	3.22	3.24	3.28	-0.06
1,1,1,2-Tetrafluoro-2-(trifluoromethoxy)butane	4.66	4.72	4.9	-0.24
1,1,1,3,3,3-Hexafluoro-2-fluoromethoxypropane	4.41	4.59		-0.18
1,1,1,3,3,3-Hexafluoro-2-methoxy-2-(trifluoromethyl)propane	4.53	4.74	4.74	-0.21
1,1,1,3,3,3-Hexafluoro-2-methoxypropane	4.56	4.86	4.84	-0.28
1,1,1,3,3,3-Hexafluoropropane	5.43	5.59	5.58	-0.15
1,1,1,3,3-Pentafluoro-3-methoxy-2-trifluoromethylpropane	4.23	4.53	4.5	-0.27
1,1,1,3,3-Pentafluorobutane	4.75	5.13	5.16	-0.41
1,1,1,3,3-Pentafluoropropane	5.48	5.28	5.28	0.2
1,1,1-Trichloroethane	4.21	4.31	4.33	-0.12
1,1,1-Trichloropropane	3.62	3.84	3.87	-0.25
1,1,1-Trichlorotrifluoroethane	4.68	4.4	4.38	0.3
1,1,1-Trifluoro-2-(1,1,2-trifluoroethoxy)ethane	4.29	4.65	4.72	-0.43
1,1,1-Trifluoro-2-(2,2,2-trifluoroethoxy)ethane	4.32	4.81	4.83	-0.51
1,1,1-Trifluoroethane	6.1	5.97	5.99	0.11
1,1,2,2,3,3,4,4-Octafluoro-5-methoxypentane	3.17	3.58	3.6	-0.43
1,1,2,2,3,3-Hexafluoro-1-methoxypropane	4.26	4.44	4.42	-0.16
1,1,2,2,3-Pentafluoropropane	4.99	5.1	5.09	-0.1
1,1,2,2-Tetrachloro-1,2-difluoroethane	3.82	3.96		-0.14
1,1,2,2-Tetrachloroethane	2.84	2.82	2.82	0.02
1,1,2,2-Tetrafluoro-1-(2,2,2-trifluoroethoxy)ethane	4.45	4.62	4.62	-0.17
1,1,2,2-Tetrafluoro-1-fluoromethoxyethane	4.5	4.57		-0.07
1,1,2,2-Tetrafluoro-3-(1,1,2,2-tetrafluoroethoxy)propane	3.74	3.94	3.97	-0.23
1,1,2,2-Tetrafluoro-3-methoxypropane	4.15	4.32	4.34	-0.19
1,1,2,2-Tetrafluoro-3-pentafluoroethoxypropane	4.35	4.25	4.21	0.14
1,1,2,2-Tetrafluoro-3-trifluoromethoxypropane	4.65	4.91	4.91	-0.26
1,1,2-Trichloroethane	3.5	3.28	3.26	0.24
1,1,2-Trichloropropane	2.61	3.13	3.19	-0.58
1,1,2-Trifluoro-1-methoxyethane	4.69	4.84	4.86	-0.17
1,1,2-Trifluoroethane	5.33	5.47	5.51	-0.18
1,1,3-Trimethylcyclopentane	3.73	3.79	3.78	-0.05
1,10-Decanediol	-2.3	-2.55	-2.56	0.26
1,10-Decanediol dinitrate	-2	-1.62	-1.59	-0.41
1,11-Undecanediol	-3.08	-3.01	-3.01	-0.07
1,12-Dodecanediol	-3.57	-3.48	-3.49	-0.08
1,13-Tridecanediol	-3.99	-3.95	-3.99	0
1,14-Tetradecanediol	-4.45	-4.42	-4.44	-0.01
1,15-Pentadecanediol	-4.92	-4.89	-4.91	-0.01
1,16-Hexadecanediol	-5.25	-5.36	-5.36	0.11

1,1-Bis(4-methylphenyl)ethane	-0.86	-0.6	-0.57	-0.29
1,1-Dichloro-1-fluoroethane	4.9	4.92		-0.02
1,1-Dichlorobutane	3.48	3.41	3.44	0.04
1,1-Dichloroethane	4.48	4.35	4.35	0.13
1,1-Dichloropropane	3.76	3.88	3.9	-0.14
1,1-Dichlorotetrafluoroethane	5.34	5.01	4.95	0.39
1,1-Diethylcyclohexane	2.23	2.33	2.36	-0.13
1,1-Difluoro-2-methoxyethane	4.6	4.69	4.64	-0.04
1,1-Difluoroethane	5.78	5.66	5.66	0.12
1,1-Difluoroethylene	6.56	6.32	6.3	0.26
1,1-Dimethylcyclohexane	3.48	3.27	3.26	0.22
1,1-Dimethylcyclopentane	4.05	4	4.01	0.04
1,1-Diphenylbutane	-0.63	-1.02	-1.08	0.45
1,1-Diphenylethane	-0.2	-0.08	-0.07	-0.13
1,1-Diphenylpropane	-0.26	-0.55	-0.61	0.35
1,2,3,4,5,6,7-Heptachloronaphthalene	-3.65	-3.48	-3.51	-0.14
1,2,3,4,5,6,8-Heptachloronaphthalene	-3.61	-3.48	-3.45	-0.16
1,2,3,4,5,7-Hexachloronaphthalene	-2.87	-2.84	-2.85	-0.02
1,2,3,4,6,7,8-Heptachlorodibenzofuran	-5.71	-5.94	-5.92	0.21
1,2,3,4,6,7,8-Heptachlorodibenzo-p-dioxin	-6.69	-6.42	-6.36	-0.33
1,2,3,4,6,7-Hexachloronaphthalene	-2.92	-2.84	-2.84	-0.08
1,2,3,4,6,8,9-Heptachlorodibenzofuran	-6.09	-5.94	-5.97	-0.12
1,2,3,4,6,8-Hexachlorodibenzofuran	-5.12	-5.3	-5.31	0.2
1,2,3,4,6-Pentachloronaphthalene	-2.32	-2.2	-2.24	-0.08
1,2,3,4,7,8,9-Heptachlorodibenzofuran	-5.88	-5.94	-5.92	0.04
1,2,3,4,7,8-Hexachlorodibenzofuran	-5.11	-5.3	-5.27	0.17
1,2,3,4,7,8-Hexachlorodibenzo-p-dioxin	-5.84	-5.78	-5.79	-0.05
1,2,3,4,7-Pentachlorodibenzofuran	-4.61	-4.66	-4.62	0.01
1,2,3,4,7-Pentachlorodibenzo-p-dioxin	-5.34	-5.14	-5.15	-0.19
1,2,3,4-Tetrachlorobenzene	0.32	0.94	0.93	-0.61
1,2,3,4-Tetrachlorodibenzofuran	-3.93	-4.02	-3.99	0.06
1,2,3,4-Tetrachloronaphthalene	-1.76	-1.56	-1.61	-0.15
1,2,3,4-Tetrafluorobenzene	3.82	3.9	3.88	-0.06
1,2,3,5,6,7-Hexachloronaphthalene	-2.92	-2.84	-2.88	-0.04
1,2,3,5,7,8-Hexachloronaphthalene	-3	-2.84	-2.87	-0.13
1,2,3,5,7-Pentachloronaphthalene	-2.23	-2.2	-2.23	0
1,2,3,5,8-Pentachloronaphthalene	-2.48	-2.2	-2.17	-0.31
1,2,3,5-Tetrachlorobenzene	1.24	0.94	0.95	0.29
1,2,3,5-Tetrachloronaphthalene	-1.75	-1.56	-1.57	-0.18
1,2,3,5-Tetraethylbenzene	0.36	0.58	0.65	-0.29
1,2,3,5-Tetrafluorobenzene	3.99	3.9	3.9	0.09
1,2,3,5-Tetramethylbenzene	1.81	2.46	2.53	-0.72

1,2,3,6,7,8-Hexachlorodibenzofuran	-5.14	-5.3	-5.31	0.18
1,2,3,6,7,8-Hexachlorodibenzo-p-dioxin	-6.07	-5.78	-5.81	-0.26
1,2,3,7,8,9-Hexachlorodibenzofuran	-5.3	-5.3	-5.28	-0.02
1,2,3,7,8,9-Hexachlorodibenzo-p-dioxin	-6.07	-5.78	-5.78	-0.29
1,2,3,7,8-Pentachlorodibenzofuran	-4.49	-4.66	-4.63	0.14
1,2,3,7,8-Pentachlorodibenzo-p-dioxin	-5.38	-5.14	-5.15	-0.23
1,2,3,7-Tetrachlorodibenzofuran	-3.85	-4.02	-3.99	0.14
1,2,3,7-Tetrachlorodibenzo-p-dioxin	-4.57	-4.5	-4.51	-0.06
1,2,3-Trichlorobenzene	1.44	1.58	1.57	-0.13
1,2,3-Trichloronaphthalene	-1.19	-0.92	-0.92	-0.27
1,2,3-Trichloropropane	2.71	2.52	2.53	0.18
1,2,3-Triethylbenzene	1.26	1.31	1.32	-0.06
1,2,4,5,6,8-Hexachloronaphthalene	-3.02	-2.84	-2.87	-0.15
1,2,4,5-Tetrachlorobenzene	1.37	0.94	0.93	0.44
1,2,4,5-Tetrafluorobenzene	3.88	3.9	3.97	-0.09
1,2,4,5-Tetramethylbenzene	1.82	2.46	2.45	-0.63
1,2,4,6,7,8-Hexachlorodibenzofuran	-5.35	-5.3	-5.26	-0.09
1,2,4,6,7,9-Hexachlorodibenzo-p-dioxin	-5.87	-5.75	-5.73	-0.14
1,2,4,6,8,9-Hexachlorodibenzofuran	-5.04	-5.3	-5.28	0.25
1,2,4,7,8-Pentachlorodibenzofuran	-4.63	-4.66	-4.69	0.06
1,2,4,7,8-Pentachlorodibenzo-p-dioxin	-5.22	-5.14	-5.08	-0.14
1,2,4,7-Tetrachloronaphthalene	-1.61	-1.56	-1.58	-0.03
1,2,4-Trichlorobenzene	1.59	1.58	1.56	0.03
1,2,4-Trichlorodibenzo-p-dioxin	-4	-3.86	-3.87	-0.13
1,2,4-Triethylbenzene	1.54	1.31	1.3	0.24
1,2,4-Trimethylbenzene	2.44	2.72	2.72	-0.28
1,2,7,8-Tetrachlorodibenzofuran	-3.98	-3.97	-3.98	0
1,2,7,8-Tetrachlorodibenzo-p-dioxin	-4.36	-4.5	-4.49	0.13
1,2-Benzenedicarboxylic acid, di-2-propenyl ester	-1.67	-1.3	-1.32	-0.35
1,2-Bis(2-aminoethoxy)ethane	0.73	0.96	0.99	-0.26
1,2-Bis(dimethylamino)ethane	3.33	3.3	3.33	0
1,2-Butanediamine	2.63	2.83	2.83	-0.2
1,2-Butanediol	0.98	0.8	0.77	0.21
1,2-Dibromobenzene	1.42	1.64	1.67	-0.25
1,2-Dibromobutane	2.61	2.47	2.5	0.11
1,2-Dibromoethane	3.17	3.2	3.17	0
1,2-Dibromoheptane	1.33	1.06	1.05	0.28
1,2-Dibromopropane	3.02	2.94	2.93	0.09
1,2-Dibromotetrafluoroethane	4.72	4.56		0.16
1,2-Dichloro-1,1,2-trifluoroethane	4.94	4.99	4.98	-0.04
1,2-Dichlorobenzene	2.26	2.22	2.19	0.07
1,2-Dichlorobutane	3.35	3.12	3.13	0.22

1,2-Dichloronaphthalene	-0.48	-0.28	-0.31	-0.17
1,2-Dichloropropane	3.81	3.59	3.61	0.2
1,2-Diethylbenzene	2.14	2.04	2.01	0.13
1,2-Difluorobenzene	3.63	3.7	3.67	-0.04
1,2-Dimethylbenzene	2.95	2.98	2.95	0
1,2-Dimethylnaphthalene	-0.06	0.48	0.5	-0.56
1,2-Diphenylethane	-0.13	-0.34	-0.36	0.23
1,2-Ethanediamine	3.24	3.46	3.46	-0.22
1,2-Ethanediol	1.08	1.22	1.21	-0.13
1,2-Ethanediol dinitrate	2.29	2.14	2.12	0.17
1,2-Ethanedithiol	3.03	3.18	3.19	-0.16
1,2-Hexanediol	0.13	-0.14	-0.14	0.27
1,2-Pentadiene	4.69	4.61		0.08
1,2-Pentanediol	0.54	0.33	0.33	0.21
1,2-Propanediol	1.24	1.27	1.24	0
1,3,5,7-Tetrachloronaphthalene	-1.38	-1.56	-1.57	0.19
1,3,5,8-Tetrachloronaphthalene	-1.68	-1.56	-1.59	-0.09
1,3,5-Tribromobenzene	0.72	0.71	0.68	0.03
1,3,5-Trichlorobenzene	1.8	1.58	1.55	0.24
1,3,5-Trifluorobenzene	4.14	3.8	3.78	0.36
1,3,5-Trimethylbenzene	2.53	2.72	2.73	-0.2
1,3,6,8-Tetrachlorodibenzofuran	-3.53	-4.02	-4.04	0.51
1,3,6,8-Tetrachlorodibenzo-p-dioxin	-4.17	-4.5	-4.51	0.34
1,3,7,8-Tetrachlorodibenzofuran	-3.7	-4.02	-3.98	0.28
1,3,7,8-Tetrachlorodibenzo-p-dioxin	-4.38	-4.5	-4.49	0.11
1,3,7,9-Tetrachlorodibenzofuran	-3.75	-4.02	-4	0.25
1,3,7,9-Tetrachlorodibenzo-p-dioxin	-4.26	-4.5	-4.47	0.21
1,3,7-Trichlorodibenzo-p-dioxin	-4.44	-3.86	-3.8	-0.64
1,3,7-Trichloronaphthalene	-0.94	-0.92	-0.97	0.03
1,3-Butanediol dinitrate	1.59	1.44	1.39	0.19
1,3-Butyleneglycol	0.4	0.8	0.8	-0.4
1,3-Dibromobenzene	1.55	1.64	1.67	-0.12
1,3-Dibromopropane	2.26	2.73	2.82	-0.56
1,3-Dichloro-2-propanol	2.02	1.83	1.83	0.19
1,3-Dichlorobenzene	2.39	2.22	2.23	0.16
1,3-Dichloronaphthalene	-0.46	-0.28	-0.31	-0.15
1,3-Dichloropropane	3.39	3.27	3.26	0.13
1,3-Dichloro-trans-2-butene	3.16	3.26	3.28	-0.12
1,3-Diethylbenzene	2.17	2.04	2.06	0.11
1,3-Dihydroisobenzofuran	1.92	1.35	1.3	0.62
1,3-Diisopropylbenzene	1.74	1.62	1.63	0.11
1,3-Diisopropylnaphthalene	-0.67	-0.88	-0.88	0.21

1,3-Dimethylbenzene	3.04	2.98	2.97	0.07
1,3-Dimethylnaphthalene	0.29	0.48	0.47	-0.18
1,3-Dioxane	3.64	3.45	3.38	0.26
1,3-Dioxolane	4.04	3.94	3.91	0.13
1,3-Divinylbenzene	1.97	2.38	2.33	-0.36
1,3-Pentanediamine	2.32	2.36	2.37	-0.05
1,3-Propanediamine	2.76	2.99	3	-0.24
1,3-Propanediol	0.79	0.75	0.74	0.05
1,3-Propanediol dinitrate	1.73	1.67	1.7	0.03
1,4,5-Trimethylnaphthalene	-0.17	0.22	0.2	-0.37
1,4-Butanediamine	2.28	2.52	2.52	-0.24
1,4-Butanediol dinitrate	1.13	1.2	1.16	-0.03
1,4-Butanedithiol	1.98	2.24	2.24	-0.26
1,4-Dichlorobenzene	2.38	2.22	2.25	0.13
1,4-Dichlorobutane	2.76	2.8	2.81	-0.05
1,4-Dichloronaphthalene	-0.38	-0.28	-0.31	-0.07
1,4-Diethylbenzene	2.14	2.04	2.06	0.08
1,4-Diisopropylbenzene	1.52	1.62	1.63	-0.11
1,4-Dimethylbenzene	3.08	2.98	2.97	0.11
1,4-Dimethylnaphthalene	0.36	0.48	0.48	-0.12
1,4-Dimethylpiperazine	3.19	3.22	3.2	-0.01
1,4-Dioxane	3.71	4.12	4.13	-0.42
1,4-Di-t-butylbenzene	0.9	0.98	1.01	-0.11
1,4-Pentadiene	4.99	4.81	4.82	0.17
1,5-Diamino-2-methylpentane	1.64	1.84	1.83	-0.19
1,5-Dichloronaphthalene	-0.45	-0.28	-0.29	-0.16
1,5-Dichloropentane	2.17	2.33	2.36	-0.19
1,5-Dimethylnaphthalene	0.29	0.48	0.49	-0.2
1,5-Hexadiene	4.47	4.34	4.36	0.11
1,5-Pentanediamine	1.85	2.05	2.04	-0.19
1,5-Pentanediol	-0.27	-0.19	-0.21	-0.06
1,5-Pentanediol dinitrate	0.74	0.73	0.7	0.04
1,5-Pentanedithiol	1.57	1.77	1.77	-0.2
1,6,7-Trimethylnaphthalene	-0.34	0.22	0.2	-0.54
1,6-Dichloronaphthalene	-0.45	-0.28	-0.31	-0.14
1,6-Dimethylnaphthalene	0.31	0.48	0.45	-0.14
1,6-Hexanediamine	1.64	1.58	1.56	0.08
1,6-Hexanedioic acid	-3.37	-3.48	-3.5	0.13
1,6-Hexanediol dinitrate	0.05	0.26	0.25	-0.2
1,6-Hexanedithiol	1.14	1.3	1.27	-0.13
1,7-Dichloronaphthalene	-0.45	-0.28	-0.31	-0.14
1,7-Heptanediamine	1.24	1.11	1.09	0.15

1,7-Heptanediol	-1.22	-1.13	-1.15	-0.07
1,7-Heptanediol dinitrate	-0.46	-0.21	-0.24	-0.22
1,7-Heptanedithiol	0.84	0.83	0.83	0.01
1,8-Cineole	1.96	2.15	2.09	-0.13
1,8-Dichloronaphthalene	-0.7	-0.28	-0.31	-0.39
1,8-Octanediamine	0.74	0.64	0.63	0.11
1,8-Octanediol	-1.74	-1.61	-1.6	-0.14
1,8-Octanediol dinitrate	-1.05	-0.68	-0.65	-0.4
1,9-Nonanediol	-2.14	-2.07	-2.07	-0.07
1-Acetyladamantane	0.22	-0.05	-0.13	0.35
1-Adamantanol	0.29	0.09	-0.05	0.34
1-Amino-3-methoxypropane	3.28	3.23	3.18	0.1
1-Azidooctane	1.63	1.35	1.32	0.31
1-Benzylpyrazole	0.17	0.21	0.19	-0.02
1-Bromo-2-chloro-1,1,2-trifluoroethane	4.56	4.62		-0.06
1-Bromo-2-chloroethane	3.65	3.47	3.46	0.19
1-Bromo-2-ethylbenzene	1.52	1.84	1.82	-0.3
1-Bromo-2-fluorobenzene	2.68	2.67	2.65	0.03
1-Bromo-3-fluorobenzene	2.85	2.67	2.66	0.19
1-Bromo-4-chlorobenzene	1.83	1.93	1.96	-0.13
1-Bromo-4-fluorobenzene	2.78	2.67	2.7	0.08
1-Bromobutane	3.74	3.6	3.61	0.13
1-Bromodecane	0.72	0.78	0.78	-0.06
1-Bromododecane	-0.28	-0.16	-0.16	-0.12
1-Bromonaphthalene	0.13	0.07	0.04	0.09
1-Bromononane	1.21	1.25	1.25	-0.04
1-Bromooctane	1.71	1.72	1.72	-0.01
1-Bromopentane	3.23	3.13	3.13	0.1
1-Bromoperfluorooctane	2.84	3.09		-0.25
1-Bromopropane	4.04	4.07	4.07	-0.03
1-Bromoundecane	0.22	0.31	0.32	-0.1
1-Butanethiol	3.79	3.59	3.58	0.21
1-Butanol	2.95	2.98	2.98	-0.03
1-Butene	5.23	5.11	5.09	0.14
1-Butylamine	3.79	3.73	3.73	0.06
1-Butyne	5.28	5.14	5.15	0.12
1-Chloro-1,1-difluoroethane	5.54	5.59	5.61	-0.07
1-Chloro-2-fluorobenzene	3.11	2.96	3	0.11
1-Chloro-3-fluorobenzene	3.25	2.96	2.94	0.31
1-Chloro-4-fluorobenzene	3.43	2.96	2.95	0.48
1-Chlorobutane	3.91	3.87	3.86	0.05
1-Chlorodecane	1.02	1.05	1.06	-0.04

1-Chlorodibenzo-p-dioxin	-1.92	-2.58	-2.61	0.69
1-Chlorododecane	-0.1	0.11	0.12	-0.22
1-Chloroheptane	2.58	2.46	2.47	0.11
1-Chlorohexane	3.1	2.93	2.93	0.17
1-Chloronaphthalene	0.58	0.36	0.33	0.25
1-Chlorononane	1.54	1.52	1.54	0
1-Chlorooctane	2.11	1.99	2.01	0.1
1-Chloropentadecane	-0.85	-1.3	-1.28	0.43
1-Chloropentane	3.62	3.4	3.41	0.21
1-Chloropropane	4.53	4.34	4.35	0.18
1-Chlorotridecane	-0.37	-0.36	-0.37	0
1-Chloroundecane	0.47	0.58	0.59	-0.12
1-Decene	2.37	2.29	2.31	0.06
1-Decylazide	0.63	0.41	0.36	0.27
1-Difluoromethoxy-1,1,2-trifluoroethane	4.69	4.7	4.67	0.02
1-Dodecanol	-0.93	-0.78	-0.77	-0.16
1-Dodecene	1.42	1.35	1.37	0.05
1-Ethoxy-1,1,2,2,3,3,3-heptafluoropropane	4.55	4.47	4.45	0.1
1-Ethoxy-1,1,2,2,3,3,4,4,4-nonafluorobutane	4.13	4.1	4.13	0
1-Ethoxy-1,1,2,2,3,3,4,4,5,5,5-undecafluoropentane	3.7	3.73	3.76	-0.06
1-Ethoxy-1,1,2,2,3,4,4,4-octafluoro-3-trifluoromethylbutane	3.71	3.75	3.73	-0.02
1-Ethoxy-1,1,2,2-tetrafluoroethane	4.46	4.53	4.56	-0.1
1-Ethyl-1-methylcyclopentane	3.41	3.53	3.5	-0.09
1-Ethyl-2-isopropylbenzene	2.03	1.83	1.81	0.22
1-Ethyl-3-isopropylbenzene	2.03	1.83	1.83	0.2
1-Ethyl-4-isopropylbenzene	1.94	1.83	1.83	0.11
1-Ethylnaphthalene	0.41	0.27	0.25	0.16
1-Ethylpyrazole	2.98	2.85		0.13
1-Ethylthiooctane	1.08	0.94	0.95	0.13
1-Fluorobutane	4.89	4.75	4.75	0.14
1-Fluorodecane	1.93	1.93	1.94	-0.01
1-Fluorohexane	3.85	3.81	3.81	0.04
1-Fluorononane	2.41	2.4	2.4	0.01
1-Fluorooctane	2.9	2.87	2.85	0.05
1-Fluoropentane	4.39	4.28	4.29	0.1
1-Fluoropropane	5.43	5.22	5.2	0.23
1-Heneicosanol	-4.82	-5.01	-5.03	0.21
1-Heptadecanol	-3.16	-3.13	-3.14	-0.02
1-Heptanethiol	2.25	2.18	2.18	0.07
1-Heptanol	1.46	1.57	1.58	-0.12
1-Heptene	3.87	3.7	3.72	0.15

1-Heptyne	3.75	3.73	3.74	0
1-Hexadecanol	-2.62	-2.66	-2.65	0.03
1-Hexanethiol	2.78	2.65	2.65	0.13
1-Hexanol	2.09	2.04	2.04	0.05
1-Hexene	4.18	4.17	4.18	0
1-Hexyne	4.26	4.2	4.22	0.04
1H-Perfluorooctane	3.34	3.53	3.57	-0.23
1-Iodobutane	3.26	3.13	3.12	0.14
1-Iododecane	0.28	0.31	0.32	-0.04
1-Iodododecane	-0.82	-0.63	-0.6	-0.22
1-Iodoheptane	1.78	1.72	1.72	0.06
1-Iodohexane	2.28	2.19	2.19	0.09
1-Iodononane	0.78	0.78	0.8	-0.02
1-Iodooctane	1.28	1.25	1.24	0.04
1-Iodopentane	2.77	2.66	2.66	0.11
1-Iodoundecane	-0.35	-0.16	-0.13	-0.22
1-Isobutylimidazole	0.98	0.86	0.86	0.12
1-Isopropylimidazole	1.23	1.38	1.45	-0.22
1-Methoxy-2-propanol	3.22	3	2.98	0.24
1-Methyl-1H-pyrazole	3.18	3.26		-0.08
1-Methyl-3-phenoxybenzene	-0.01	-0.02	-0.04	0.03
1-Methyl-4-benzylbenzene	-0.14	-0.13	-0.18	0.04
1-Methyl-4-isopropylcyclohexene	2.26	1.82	1.8	0.46
1-Methyl-4-vinylcyclohexene	2.76	2.2	2.17	0.59
1-Methylfluorene	-1.15	-1.83	-1.76	0.61
1-Methylnaphthalene	0.97	0.74	0.75	0.22
1-Methylphenanthrene	-1.75	-1.76	-1.82	0.08
1-Methylpiperazine	2.97	3.13	3.19	-0.22
1-Naphthylamine	-0.77	-0.95	-0.99	0.22
1-Nitro-2,6-diisopropylbenzene	0.1	-0.42	-0.45	0.55
1-Nitro-2-isopropylbenzene	0.8	0.52	0.51	0.29
1-Nitrobutane	2.71	2.7	2.77	-0.06
1-Nitronaphthalene	-0.7	-1.04	-1.08	0.39
1-Nitropentane	2.27	2.23	2.22	0.05
1-Nitropropane	3.37	3.17	3.13	0.24
1-Nitropyrene	-6.23	-5.68	-5.66	-0.57
1-Nonadecanol	-3.96	-4.07	-4.07	0.11
1-Nonanol	0.5	0.63	0.64	-0.14
1-Nonene	2.87	2.76	2.78	0.09
1-Nonyne	2.69	2.79	2.8	-0.11
1-Octadecanol	-3.55	-3.6	-3.6	0.05
1-Octanethiol	1.79	1.71	1.7	0.09

1-Octanol	1.06	1.1	1.12	-0.06
1-Octyne	3.24	3.26	3.28	-0.04
1-Pentadecanol	-2.23	-2.19	-2.2	-0.03
1-Pentadecene	-0.11	-0.06	-0.05	-0.06
1-Pentanethiol	3.27	3.12	3.12	0.14
1-Pentanol	2.52	2.51	2.52	0
1-Pentene	4.72	4.64	4.66	0.06
1-Pentyne	4.76	4.67	4.68	0.08
1-Phenyldodecane	-1.74	-1.93	-1.95	0.21
1-Phenylimidazole	-0.39	-0.3		-0.09
1-Phenylnaphthalene	-2.5	-1.9	-1.93	-0.57
1-Piperazineethanamine	0.91	1.51	1.47	-0.56
1-Propanethiol	4.11	4.06	4.06	0.05
1-Propanol	3.45	3.45	3.44	0.01
1-Propoxy-2-propanol	2.37	2.25	2.25	0.12
1-Propylamine	4.32	4.2	4.21	0.11
1-Pyrenecarboxaldehyde	-5.27	-4.94	-4.98	-0.28
1-s-Butylimidazole	0.95	0.91	0.85	0.1
1-t-Butyl-4-ethylbenzene	1.28	1.51	1.53	-0.25
1-t-Butylimidazole	1.13	1.23	1.29	-0.16
1-Tetradecene	0.37	0.41	0.41	-0.04
1-Tetradecylamine	-0.52	-0.97	-0.96	0.44
1-trans-5-trans-9-cis-Cyclododecatriene	1.07	0.56	0.5	0.57
1-Tridecanol	-1.37	-1.25	-1.27	-0.1
1-Tridecene	0.95	0.88	0.87	0.08
1-Undecanol	-0.38	-0.31	-0.31	-0.07
1-Undecene	2.01	1.82	1.84	0.17
2,2,2-Trifluoroethanol	3.98	4.01	4.01	-0.03
2,2',3,3',4,4',5,5',6-Nonachlorobiphenyl	-4.97	-5.16	-5.17	0.2
2,2',3,3',4,4',5,5',6-Nonachlorodiphenyl ether	-5.16	-5.52	-5.49	0.33
2,2',3,3',4,4',5,5'-Octachlorobiphenyl	-4.69	-4.52	-4.55	-0.14
2,2',3,3',4,4',5,5'-Octachlorodiphenyl ether	-4.76	-4.88	-4.89	0.13
2,2',3,3',4,4',5,6,6'-Nonachlorobiphenyl	-4.5	-5.16	-5.21	0.71
2,2',3,3',4,4',5,6'-Octachlorobiphenyl	-4.46	-4.52	-4.54	0.08
2,2',3,3',4,4',5,6-Octachlorobiphenyl	-4.66	-4.52	-4.51	-0.15
2,2',3,3',4,4',5,6'-Octachlorodiphenyl ether	-5.05	-4.88	-4.92	-0.13
2,2',3,3',4,4',5-Heptachlorobiphenyl	-4.07	-3.88	-3.93	-0.14
2,2',3,3',4,4',6,6'-Octachlorobiphenyl	-4.04	-4.52	-4.49	0.45
2,2',3,3',4,4',6,6'-Octachlorodiphenyl ether	-4.76	-4.88	-4.92	0.16
2,2',3,3',4,4',6-Heptachlorobiphenyl	-3.73	-3.88	-3.88	0.15
2,2',3,3',4,4'-Hexachlorobiphenyl	-3.45	-3.24	-3.27	-0.17
2,2',3,3',4,4'-Hexachlorodiphenyl ether	-4.06	-3.6	-3.61	-0.45

2,2',3,3',4,5,5',6,6'-Nonachlorobiphenyl	-5.42	-5.16	-5.12	-0.3
2,2',3,3',4,5,5',6'-Octachlorobiphenyl	-4.3	-4.52	-4.57	0.27
2,2',3,3',4,5,5'-Heptachlorobiphenyl	-3.85	-3.88	-3.89	0.04
2,2',3,3',4,5,6,6'-Octachlorobiphenyl	-4.4	-4.52	-4.55	0.15
2,2',3,3',4,5',6,6'-Octachlorobiphenyl	-4.23	-4.52	-4.53	0.3
2,2',3,3',4,5,6'-Heptachlorobiphenyl	-3.92	-3.88	-3.88	-0.04
2,2',3,3',4,5',6'-Heptachlorobiphenyl	-3.86	-3.88	-3.9	0.04
2,2',3,3',4,5,6-Heptachlorobiphenyl	-4.26	-3.88	-3.9	-0.36
2,2',3,3',4,5',6-Heptachlorobiphenyl	-3.4	-3.88	-3.93	0.53
2,2',3,3',4,5'-Hexachlorobiphenyl	-3.27	-3.24	-3.23	-0.04
2,2',3,3',4,5-Hexachlorobiphenyl	-3.24	-3.24	-3.26	0.02
2,2',3,3',4,6,6'-Heptachlorobiphenyl	-3.38	-3.88	-3.93	0.55
2,2',3,3',4,6'-Hexachlorobiphenyl	-3.25	-3.24	-3.25	0
2,2',3,3',4,6-Hexachlorobiphenyl	-3.16	-3.24	-3.23	0.07
2,2',3,3',4-Pentabromodiphenyl ether	-4.32	-4.41	-4.43	0.11
2,2',3,3',4-Pentachlorobiphenyl	-2.7	-2.6	-2.6	-0.1
2,2',3,3',5,5',6,6'-Octachlorobiphenyl	-4.54	-4.52	-4.53	-0.01
2,2',3,3',5,5',6-Heptachlorobiphenyl	-3.34	-3.88	-3.88	0.54
2,2',3,3',5,5'-Hexachlorobiphenyl	-3.1	-3.24	-3.27	0.17
2,2',3,3',5,6,6'-Heptachlorobiphenyl	-3.3	-3.88	-3.9	0.6
2,2',3,3',5,6'-Hexachlorobiphenyl	-3.13	-3.24	-3.26	0.13
2,2',3,3',5,6-Hexachlorobiphenyl	-3.32	-3.24	-3.25	-0.07
2,2',3,3',5-Pentachlorobiphenyl	-2.52	-2.6	-2.65	0.13
2,2',3,3',6,6'-Hexachlorobiphenyl	-3.28	-3.24	-3.23	-0.05
2,2',3,3',6-Pentachlorobiphenyl	-2.43	-2.6	-2.65	0.22
2,2',3,3'-Tetrachlorobiphenyl	-2.01	-1.96	-1.97	-0.04
2,2,3,3-Tetramethylhexane	2.73	2.94	2.99	-0.26
2,2,3,3-Tetramethylpentane	3.12	3.41	3.44	-0.32
2,2',3,4,4',5,5',6-Octachlorobiphenyl	-4.46	-4.52	-4.55	0.09
2,2',3,4,4',5,5'-Heptachlorobiphenyl	-4.02	-3.88	-3.9	-0.12
2,2',3,4,4',5,6,6'-Octachlorobiphenyl	-3.86	-4.52	-4.57	0.71
2,2',3,4,4',5,6-Heptachlorobiphenyl	-3.54	-3.88	-3.87	0.34
2,2',3,4,4',5',6-Heptachlorobiphenyl	-3.7	-3.88	-3.86	0.16
2,2',3,4,4',5,6'-Heptachlorobiphenyl	-3.67	-3.88	-3.9	0.23
2,2',3,4,4',5,6'-Heptachlorodiphenyl ether	-4	-4.24	-4.28	0.28
2,2',3,4,4',5'-Hexachlorobiphenyl	-3.34	-3.24	-3.25	-0.09
2,2',3,4,4',5-Hexachlorobiphenyl	-3.42	-3.24	-3.29	-0.13
2,2',3,4,4',5'-Hexachlorodiphenyl ether	-3.77	-3.6	-3.57	-0.2
2,2',3,4,4',5-Hexachlorodiphenyl ether	-3.7	-3.6	-3.59	-0.11
2,2',3,4,4',6,6'-Heptachlorodiphenyl ether	-3.88	-4.24	-4.27	0.39
2,2',3,4,4',6'-Hexachlorobiphenyl	-2.9	-3.24	-3.25	0.35
2,2',3,4,4',6-Hexachlorobiphenyl	-2.9	-3.24	-3.26	0.36

2,2',3,4,4',6'-Hexachlorodiphenyl ether	-3.57	-3.6	-3.57	0
2,2',3,4,4'-Pentabromodiphenyl ether	-4.55	-4.41	-4.4	-0.15
2,2',3,4,4'-Pentachlorobiphenyl	-2.63	-2.6	-2.61	-0.02
2,2',3,4,4'-Pentachlorodiphenyl ether	-3.16	-2.96	-3	-0.16
2,2',3,4,5,5',6-Heptabromodiphenyl ether	-6.33	-6.27	-6.39	0.06
2,2',3,4,5,5',6-Heptachlorobiphenyl	-4.32	-3.88	-3.89	-0.43
2,2',3,4',5,5',6-Heptachlorobiphenyl	-3.52	-3.88	-3.88	0.36
2,2',3,4,5,5'-Hexachlorobiphenyl	-3.3	-3.24	-3.24	-0.06
2,2',3,4',5,5'-Hexachlorobiphenyl	-3.14	-3.24	-3.27	0.13
2,2',3,4,5,6,6'-Heptachlorobiphenyl	-3.33	-3.88	-3.9	0.57
2,2',3,4,5,6'-Hexachlorobiphenyl	-3.13	-3.24	-3.27	0.14
2,2',3,4',5,6'-Hexachlorobiphenyl	-2.72	-3.24	-3.26	0.54
2,2',3,4,5,6-Hexachlorobiphenyl	-3.53	-3.24	-3.26	-0.27
2,2',3,4',5,6-Hexachlorobiphenyl	-3.95	-3.24	-3.26	-0.69
2,2',3,4,5',6-Hexachlorobiphenyl	-2.78	-3.24	-3.23	0.45
2,2',3,4',5',6-Hexachlorobiphenyl	-3.07	-3.24	-3.25	0.18
2,2',3,4,5-Pentachlorobiphenyl	-2.54	-2.6	-2.61	0.07
2,2',3,4',5-Pentachlorobiphenyl	-2.6	-2.6	-2.61	0.01
2,2',3,4,5'-Pentachlorobiphenyl	-2.65	-2.6	-2.65	0
2,2',3,4,6-Pentachlorobiphenyl	-2.51	-2.6	-2.59	0.08
2,2',3,4',6-Pentachlorobiphenyl	-2.42	-2.6	-2.59	0.17
2,2',3,4,6'-Pentachlorobiphenyl	-2.28	-2.6	-2.65	0.37
2,2',3,4',6'-Pentachlorobiphenyl	-2.21	-2.6	-2.65	0.44
2,2',3,4'-Tetrachlorobiphenyl	-1.88	-1.96	-2.01	0.13
2,2',3,4-Tetrachlorobiphenyl	-1.91	-1.96	-2.01	0.1
2,2,3,4-Tetramethylpentane	3.24	3.52	3.54	-0.3
2,2',3,5,5',6-Hexachlorobiphenyl	-2.72	-3.24	-3.24	0.52
2,2',3,5,5'-Pentachlorobiphenyl	-2.41	-2.6	-2.63	0.22
2,2',3,5',6-Pentachlorobiphenyl	-2.27	-2.6	-2.6	0.33
2,2',3,5'-Tetrachlorobiphenyl	-1.9	-1.96	-1.97	0.07
2,2',3,5-Tetrachlorobiphenyl	-1.74	-1.96	-1.98	0.24
2,2',3,6'-Tetrachlorobiphenyl	-1.81	-1.96	-1.98	0.17
2,2',3,6-Tetrachlorobiphenyl	-1.65	-1.96	-1.97	0.32
2,2',3-Trichlorobiphenyl	-1.37	-1.32	-1.37	0
2,2,3-Trimethylpentane	3.63	3.73	3.74	-0.11
2,2',4,4',5,5'-Hexabromodiphenyl ether	-5.24	-5.34	-5.35	0.11
2,2',4,4',5,5'-Hexachlorobiphenyl	-3.23	-3.24	-3.29	0.06
2,2',4,4',5,5'-Hexachlorodiphenyl ether	-3.46	-3.6	-3.59	0.13
2,2',4,4',5,6'-Hexabromodiphenyl ether	-5.42	-5.34	-5.38	-0.04
2,2',4,4',5,6'-Hexachlorobiphenyl	-2.77	-3.24	-3.24	0.47
2,2',4,4',5,6'-Hexachlorodiphenyl ether	-3.19	-3.6	-3.63	0.44
2,2',4,4',5-Pentabromodiphenyl ether	-4.3	-4.41	-4.42	0.12

2,2',4,4',5-Pentachlorobiphenyl	-2.54	-2.6	-2.62	0.08
2,2',4,4',5-Pentachlorodiphenyl ether	-2.87	-2.96	-3	0.13
2,2',4,4',6,6'-Hexabromobiphenyl	-5.1	-4.98	-5.03	-0.06
2,2',4,4',6,6'-Hexachlorobiphenyl	-3.07	-3.24	-3.23	0.16
2,2',4,4',6-Pentachlorodiphenyl ether	-2.66	-2.96	-3.08	0.42
2,2',4,4'-Tetrabromodiphenyl ether	-3.6	-3.48	-3.57	-0.03
2,2',4,4'-Tetrachlorobiphenyl	-1.94	-1.96	-1.97	0.03
2,2',4,4'-Tetrachlorodiphenyl ether	-2.28	-2.32	-2.29	0.01
2,2,4,4-Tetramethylpentane	3.42	3.41	3.45	-0.03
2,2',4,5,5'-PCB	-2.8	-2.6	-2.62	-0.18
2,2',4,5,5'-Pentachlorodiphenyl ether	-2.76	-2.96	-3	0.24
2,2',4,5',6-Pentachlorobiphenyl	-2.21	-2.6	-2.63	0.42
2,2',4,5,6'-Pentachlorobiphenyl	-2.18	-2.6	-2.65	0.47
2,2',4,5'-Tetrachlorobiphenyl	-1.91	-1.96	-1.98	0.07
2,2',4,5-Tetrachlorobiphenyl	-1.94	-1.96	-1.95	0.01
2,2',4,6,6'-Pentachlorobiphenyl	-2.36	-2.6	-2.6	0.24
2,2',4,6'-Tetrachlorobiphenyl	-1.58	-1.96	-2.01	0.43
2,2',4,6-Tetrachlorobiphenyl	-1.36	-1.96	-1.97	0.61
2,2',4-Tribromodiphenyl ether	-2.66	-2.55	-2.55	-0.11
2,2',4-Trichlorobiphenyl	-1.48	-1.32	-1.35	-0.12
2,2,4-Trimethyl-4-methoxypentane	2.87	3	3.05	-0.18
2,2,4-Trimethylhexane	3.32	3.26	3.28	0.04
2,2',5,5'-Tetrachlorobiphenyl	-1.9	-1.96	-1.98	0.08
2,2,5,5-Tetramethylhexane	3.07	2.94	3.01	0.06
2,2',5,6'-Tetrachlorobiphenyl	-2.31	-1.96	-1.98	-0.33
2,2',5-Trichlorobiphenyl	-1.22	-1.32	-1.33	0.11
2,2,5-Trimethylhexane	3.34	3.26	3.28	0.06
2,2',6-Trichlorobiphenyl	-1.28	-1.32	-1.37	0.09
2,2’-Diaminodiethylamine	1.24	1.67	1.73	-0.49
2,2-Dichloro-1,1,1-trifluoroethane	4.95	4.44	4.41	0.54
2,2'-Dichlorobiphenyl	-0.49	-0.68	-0.67	0.18
2,2'-Dichlorodiisopropyl ether	2.02	2.03	2.04	-0.02
2,2-Dimethyl-3-ethylpentane	3.18	3.26	3.28	-0.1
2,2-Dimethylheptane	3.2	3	3.01	0.19
2,2-Dimethylhexane	3.66	3.47	3.5	0.16
2,2-Dimethyloctane	2.69	2.53	2.55	0.14
2,2-Dimethylpentane	4.14	3.94	3.97	0.17
2,2-Dimethylpropane	5.23	4.88	4.91	0.32
2,3,3',4,4',5,5',6-Octachlorobiphenyl	-4.6	-4.52	-4.57	-0.03
2,3,3',4,4',5,5'-Heptachlorobiphenyl	-4.32	-3.88	-3.87	-0.45
2',3,3',4,4',5,6-Heptabromodiphenyl ether	-6.24	-6.27	-6.28	0.04
2,3,3',4,4',5,6-Heptachlorobiphenyl	-4.29	-3.88	-3.93	-0.36

2,3,3',4,4',5',6-Heptachlorobiphenyl	-3.98	-3.88	-3.9	-0.08
2,3,3',4,4',5-Hexabromodiphenyl ether	-5.03	-5.34	-5.45	0.43
2,3,3',4,4',5'-Hexachlorobiphenyl	-3.7	-3.24	-3.25	-0.45
2,3,3',4,4',5-Hexachlorobiphenyl	-3.68	-3.24	-3.25	-0.43
2,3,3',4,4',6-Hexachlorobiphenyl	-3.21	-3.24	-3.24	0.03
2,3,3',4,4'-Pentachlorobiphenyl	-3.06	-2.6	-2.63	-0.43
2,3,3',4,4'-Pentachlorodiphenyl ether	-3.29	-2.96	-2.97	-0.32
2,3,3',4,5,5',6-Heptachlorobiphenyl	-4.04	-3.88	-3.93	-0.11
2,3,3',4',5,5',6-Heptachlorobiphenyl	-3.72	-3.88	-3.93	0.21
2,3,3',4,5,5'-Hexachlorobiphenyl	-2.92	-3.24	-3.23	0.31
2,3,3',4',5,5'-Hexachlorobiphenyl	-2.86	-3.24	-3.23	0.37
2,3,3',4,5,6-Hexachlorobiphenyl	-3.57	-3.24	-3.29	-0.28
2,3,3',4',5,6-Hexachlorobiphenyl	-3.48	-3.24	-3.25	-0.23
2,3,3',4,5',6-Hexachlorobiphenyl	-2.86	-3.24	-3.26	0.4
2,3,3',4',5',6-Hexachlorobiphenyl	-3.35	-3.24	-3.29	-0.06
2,3,3',4',5'-Pentachlorobiphenyl	-3.11	-2.6	-2.59	-0.52
2,3,3',4,5-Pentachlorobiphenyl	-2.86	-2.6	-2.6	-0.26
2,3,3',4',5-Pentachlorobiphenyl	-2.98	-2.6	-2.6	-0.38
2,3,3',4,5'-Pentachlorobiphenyl	-2.99	-2.6	-2.61	-0.38
2,3,3',4',6-Pentachlorobiphenyl	-2.74	-2.6	-2.63	-0.11
2,3,3',4'-Tetrachlorobiphenyl	-2.29	-1.96	-1.98	-0.31
2,3,3,4-Tetramethylpentane	3.07	3.52	3.55	-0.48
2,3,3',5,5',6-Hexachlorobiphenyl	-2.89	-3.24	-3.24	0.35
2,3,3',5,5'-Pentachlorobiphenyl	-2.81	-2.6	-2.63	-0.18
2,3,3',5,6-Pentachlorobiphenyl	-2.5	-2.6	-2.62	0.12
2,3,3',5',6-Pentachlorobiphenyl	-2.62	-2.6	-2.61	-0.01
2,3,3',5-Tetrachlorobiphenyl	-2.12	-1.96	-1.98	-0.14
2,3,3',6-Tetrachlorobiphenyl	-1.81	-1.96	-1.97	0.16
2,3,3'-Trichlorobiphenyl	-1.48	-1.32	-1.37	-0.11
2,3,3-Trimethyl-1-butene	4.19	4.31	4.32	-0.13
2,3,3-Trimethylhexane	3.18	3.26	3.29	-0.11
2,3,3-Trimethylpentane	3.56	3.73	3.75	-0.19
2,3',4,4',5,5'-Hexachlorobiphenyl	-3.55	-3.24	-3.29	-0.26
2,3',4,4',5,5'-Hexachlorodiphenyl ether	-3.64	-3.6	-3.61	-0.03
2,3,4,4',5,6-Hexachlorobiphenyl	-2.93	-3.24	-3.29	0.36
2,3',4,4',5',6-Hexachlorobiphenyl	-3.27	-3.24	-3.26	-0.01
2,3',4,4',5'-Pentachlorobiphenyl	-2.88	-2.6	-2.61	-0.27
2,3,4,4',5-Pentachlorobiphenyl	-2.9	-2.6	-2.59	-0.31
2,3',4,4',5-Pentachlorobiphenyl	-2.77	-2.6	-2.65	-0.12
2,3',4,4',6-Pentabromodiphenyl ether	-4.66	-4.41	-4.51	-0.15
2,3,4,4',6-Pentachlorobiphenyl	-2.65	-2.6	-2.65	0
2,3',4,4',6-Pentachlorobiphenyl	-2.54	-2.6	-2.61	0.07

2,3',4,4'-Tetrabromodiphenyl ether	-3.72	-3.48	-3.49	-0.23
2,3',4,4'-Tetrachlorobiphenyl	-2.18	-1.96	-1.99	-0.19
2,3',4,4'-Tetrachlorodiphenyl ether	-2.39	-2.32	-2.33	-0.06
2,3',4,5,5'-Pentachlorobiphenyl	-2.85	-2.6	-2.65	-0.2
2,3',4',5,5'-Pentachlorobiphenyl	-2.95	-2.6	-2.6	-0.35
2,3,4,5,6-Pentabromodiphenyl ether	-4.3	-4.41	-4.51	0.21
2,3,4,5,6-Pentachlorobiphenyl	-2.62	-2.6	-2.61	-0.01
2,3',4,5',6-Pentachlorobiphenyl	-2.43	-2.6	-2.62	0.19
2,3,4',5,6-Pentachlorobiphenyl	-2.59	-2.6	-2.63	0.04
2,3',4',5',6-Pentachlorobiphenyl	-2.73	-2.6	-2.59	-0.14
2,3,4,5,6-Pentafluorotoluene	3.38	3.74	3.81	-0.44
2,3,4,5-Tetrachloroanisole	0.08	0.49	0.52	-0.44
2,3',4',5-Tetrachlorobiphenyl	-2.24	-1.96	-1.97	-0.27
2,3',4,5'-Tetrachlorobiphenyl	-2.17	-1.96	-1.97	-0.2
2,3',4',5'-Tetrachlorobiphenyl	-2.22	-1.96	-2.01	-0.21
2,3,4,5-Tetrachlorobiphenyl	-2.23	-1.96	-1.99	-0.24
2,3',4,5-Tetrachlorobiphenyl	-2.18	-1.96	-1.95	-0.23
2,3,4',5-Tetrachlorobiphenyl	-2.17	-1.96	-1.98	-0.19
2,3,4,6,7,8-Hexachlorodibenzofuran	-5.3	-5.3	-5.31	0.01
2,3',4,6-Tetrabromodiphenyl ether	-3.5	-3.48	-3.52	0.03
2,3',4',6-Tetrabromodiphenyl ether	-3.39	-3.48	-3.49	0.1
2,3,4,6-Tetrachlorobiphenyl	-1.78	-1.96	-1.98	0.2
2,3,4',6-Tetrachlorobiphenyl	-2.11	-1.96	-1.98	-0.13
2,3',4',6-Tetrachlorobiphenyl	-2.02	-1.96	-1.99	-0.03
2,3,4,6-Tetrachlorophenol	-0.25	-0.81	-0.83	0.58
2,3,4,7,8-Pentachlorodibenzofuran	-4.66	-4.66	-4.68	0.02
2',3,4-Tribromodiphenyl ether	-2.83	-2.55	-2.61	-0.22
2,3',4-Tribromodiphenyl ether	-2.7	-2.55	-2.63	-0.07
2,3',4-Trichlorobiphenyl	-1.43	-1.32	-1.33	-0.1
2,3,4'-Trichlorobiphenyl	-1.55	-1.32	-1.33	-0.22
2,3',4'-Trichlorobiphenyl	-1.53	-1.32	-1.34	-0.19
2,3,4-Trichlorobiphenyl	-1.53	-1.32	-1.31	-0.22
2,3,4-Trichlorophenol	0	-0.17	-0.19	0.19
2,3,4-Trimethylpentane	3.58	3.84	3.82	-0.24
2,3',5,5'-Tetrachlorobiphenyl	-2.12	-1.96	-1.99	-0.13
2,3,5,6-Tetrachlorobiphenyl	-2.33	-1.96	-1.97	-0.36
2,3',5'-Trichlorobiphenyl	-1.35	-1.32	-1.32	-0.03
2,3,5-Trichlorobiphenyl	-1.4	-1.32	-1.32	-0.08
2,3',5-Trichlorobiphenyl	-1.44	-1.32	-1.34	-0.1
2,3,6-Trichlorobiphenyl	-1.15	-1.32	-1.34	0.19
2,3',6-Trichlorobiphenyl	-1.31	-1.32	-1.34	0.03
2,3,7,8-Tetrachlorodibenzofuran	-3.91	-4.02	-4	0.09

2,3,7-Trichlorodibenzo-p-dioxin	-3.67	-3.86	-3.87	0.2
2,3,8-Trichlorodibenzofuran	-3.04	-3.38	-3.4	0.36
2,3-Butandione	3.88	3.38	3.36	0.52
2,3-Butanediol dinitrate	2.03	1.68	1.69	0.34
2,3-Butyleneglycol	1.39	1.32	1.28	0.1
2,3'-Dichlorobiphenyl	-0.78	-0.68	-0.69	-0.09
2,3-Dichlorobiphenyl	-0.79	-0.68	-0.71	-0.08
2,3-Dichlorobutane	3.51	3.44	3.49	0.02
2,3-Dichlorodibenzo-p-dioxin	-3.41	-3.22	-3.16	-0.25
2,3-Dichloronaphthalene	-0.48	-0.28	-0.33	-0.15
2,3-Dichloropropene	3.91	3.7	3.69	0.22
2,3-Dichlorotoluene	1.75	1.96	1.96	-0.21
2,3-Dimethyl-1,3-butadiene	4.31	4.74	4.81	-0.51
2,3-Dimethyl-1-butene	4.53	4.63	4.64	-0.11
2,3-Dimethyl-1-hexene	3.57	3.69	3.72	-0.15
2,3-Dimethyl-1-pentene	4.03	4.16	4.17	-0.14
2,3-Dimethyl-2-butanol	2.63	3.09	3.14	-0.51
2,3-Dimethyl-2-pentene	3.82	4.13	4.09	-0.27
2,3-Dimethylbutane	4.51	4.52	4.53	-0.02
2,3-Dimethylhexane	3.54	3.58	3.58	-0.04
2,3-Dimethylpentane	3.96	4.05	4.06	-0.1
2,3-Lutidine	2.55	2.76	2.78	-0.23
2,3-Pentadiene	4.63	4.64		-0.01
2,3-Xylenol	0.55	1.23	1.23	-0.68
2,4,4',5-Tetrachlorobiphenyl	-2.14	-1.96	-1.97	-0.17
2,4,4',5-Tetrachlorodiphenyl ether	-2.32	-2.32	-2.36	0.04
2,4,4',6-Tetrabromodiphenyl ether	-3.41	-3.48	-3.49	0.08
2,4,4'-Tribromodiphenyl ether	-2.8	-2.59	-2.61	-0.19
2,4,4'-Trichlorobiphenyl	-1.47	-1.32	-1.34	-0.13
2,4,4'-Trichlorodiphenyl ether	-1.69	-1.68	-1.72	0.03
2,4,4-Trimethyl-2-pentene	3.7	3.87	3.88	-0.18
2,4,4-Trimethylhexane	3.25	3.26	3.29	-0.04
2,4,5-T	-2.2	-2.01	-2.01	-0.19
2,4,5-Trichlorobiphenyl	-1.35	-1.32	-1.37	0.02
2,4',5-Trichlorobiphenyl	-1.46	-1.32	-1.34	-0.12
2,4,5-Trichlorodiphenyl ether	-1.54	-1.68	-1.69	0.15
2,4',5-Trichlorodiphenyl ether	-1.64	-1.68	-1.72	0.08
2,4,5-Trichlorophenol	0.43	-0.17	-0.19	0.62
2,4,6-Collidine	2.43	2.55	2.65	-0.22
2,4,6-Tribromodiphenyl ether	-2.4	-2.55	-2.59	0.19
2,4',6-Tribromodiphenyl ether	-2.72	-2.55	-2.55	-0.17
2,4,6-Tribromophenol	-0.74	-1.04	-1.08	0.34

2,4,6-Trichloroanisole	0.82	1.13	1.12	-0.3
2,4,6-Trichlorobiphenyl	-1.38	-1.32	-1.34	-0.04
2,4',6-Trichlorobiphenyl	-1.24	-1.32	-1.35	0.11
2,4,6-Trichlorodibenzofuran	-2.88	-3.38	-3.36	0.48
2,4,6-Trichlorophenol	0.02	-0.17	-0.19	0.21
2,4,6-Tri-t-butylnitrobenzene	-2.53	-2.32	-2.26	-0.27
2,4,8-Trichlorodibenzofuran	-2.86	-3.38	-3.34	0.48
2,4'-Dibromodiphenyl ether	-1.91	-1.62	-1.63	-0.28
2,4-Dibromodiphenyl ether	-1.82	-1.62	-1.61	-0.2
2,4'-Dichlorobiphenyl	-0.78	-0.68	-0.7	-0.08
2,4-Dichlorobiphenyl	-0.76	-0.68	-0.71	-0.05
2,4-Dichlorodiphenyl ether	-0.91	-1.04	-1.05	0.14
2,4-Dichlorophenoxyacetic acid	-1.96	-1.37	-1.36	-0.6
2,4-Dichlorotoluene	1.86	1.96	1.96	-0.1
2,4-Dimethyl-1-pentene	4.1	4.16	4.2	-0.1
2,4-Dimethyl-2-pentene	4.05	4.19	4.19	-0.14
2,4-Dimethyl-3-ethylpentane	3.13	3.37	3.4	-0.27
2,4-Dimethyl-3-pentanone	3.26	3.27	3.24	0.02
2,4-Dimethylhexane	3.61	3.58	3.59	0.02
2,4-Dimethylpentane	4.12	4.05	4.04	0.08
2,4-Dinitrophenol	-2.26	-2.33	-2.36	0.1
2,4-Di-t-butylphenol	-0.31	-0.77	-0.74	0.43
2,4-Lutidine	2.62	2.76	2.8	-0.18
2,4-Xylenol	1.11	1.23	1.26	-0.15
2,5-Aldehydine	2.3	2.29	2.33	-0.03
2,5-Dichlorobiphenyl	-0.75	-0.68	-0.7	-0.04
2,5-Dichlorotoluene	1.85	1.96	1.96	-0.11
2,5-Dimethyl-1,5-hexadiene	3.34	3.8	3.87	-0.53
2,5-Dimethylhexane	3.61	3.58	3.61	0
2,5-Dimethyltetrahydrofuran	3.92	4.11	4.13	-0.21
2,5-Dimethylthiophene	3.07	3.1	3.16	-0.09
2,5-Lutidine	2.65	2.76	2.76	-0.11
2,5-Xylenol	1.35	1.23	1.17	0.18
2,5-Xylidine	1.21	1.03	1.02	0.19
2,6-Dibromodiphenyl ether	-1.59	-1.62	-1.65	0.06
2,6-Dichlorobiphenyl	-0.54	-0.68	-0.7	0.16
2,6-Dichlorodiphenyl ether	-0.76	-1.04	-1.08	0.32
2,6-Dichloronaphthalene	-0.46	-0.28	-0.31	-0.15
2,6-Dichlorophenol	0.64	0.47	0.47	0.17

2,6-Dichlorosyringaldehyde	-1.6	-1.73	-1.81	0.21
2,6-Dichlorotoluene	1.85	1.96	1.95	-0.1
2,6-Diethylaniline	-0.3	0.09	0.12	-0.42
2,6-Dimethyl-4-heptanol	1.61	1.67	1.69	-0.08
2,6-Dimethylheptan-4-one	2.35	2.33	2.33	0.02
2,6-Dimethylheptane	3.09	3.11	3.12	-0.03
2,6-Dimethylnaphthalene	0.31	0.48	0.48	-0.17
2,6-Lutidine	2.88	2.81	2.74	0.14
2,6-Xylenol	1.33	1.23	1.27	0.06
2,6-Xylidine	1.23	1.03	1	0.23
2,7-Dibromofluorene	-2.96	-3.43	-3.46	0.5
2,7-Dichloronaphthalene	-0.46	-0.28	-0.25	-0.21
2,7-Diiodofluorene	-4.25	-4.23	-4.24	-0.01
2,7-Dimethyloctane	2.7	2.64	2.63	0.07
2,8-Dichlorodibenzo-p-dioxin	-3.85	-3.22	-3.23	-0.62
2-Adamantanone	0.22	0.06	-0.04	0.26
2-Azidoacetonitrile	2.46	2.97	3.19	-0.73
2-Azidoethanol	1.85	2.21	2.38	-0.53
2-Azidoethoxyethane	3.12	2.92	2.91	0.21
2-Bromo-7-chlorofluorene	-2.48	-3.14	-3.17	0.69
2-Bromo-7-iodofluorene	-3.6	-3.83	-3.82	0.22
2-Bromoadamantane	0.52	0.39	0.35	0.17
2-Bromobutane	3.9	3.81	3.81	0.09
2-Bromodiphenyl ether	-0.8	-0.73	-0.73	-0.07
2-Bromofluorene	-1.75	-2.5	-2.51	0.77
2-Bromonaphthalene	-0.3	0.07	0.02	-0.32
2-Bromopropane	4.46	4.28	4.28	0.18
2-Bromotoluene	2.27	2.31	2.32	-0.05
2-Butanethiol	4.03	3.9	3.92	0.11
2-Butanol	3.41	3.5	3.52	-0.11
2-Butanone	4.12	4.16	4.16	-0.04
2-Butyne	4.97	4.9	4.91	0.06
2-Chloro-1,1,1,2-tetrafluoroethane	5.62	5.37	5.27	0.35
2-Chloro-1,1,1-trifluoroethane	5.31	4.9	4.9	0.41
2-Chloro-1,1,2-trifluoroethyl difluoromethyl ether	4.46	4.29	4.28	0.18
2-Chloro-1,1,2-trifluoroethyl ethyl ether	3.89	4.15	4.2	-0.31
2-Chloro-1,1,2-trifluoroethyl propyl ether	3.5	3.68	3.7	-0.2
2-Chloro-1,1-difluoroethylene	5.69	5.53	5.47	0.22
2-Chloroadamantane	0.79	0.77	0.7	0.09

2-Chloroaniline	1.43	0.91	0.91	0.52
2-Chlorobiphenyl	0.05	-0.04	-0.09	0.14
2-Chlorobutane	4.34	4.19	4.22	0.12
2-Chlorodiphenyl ether	-0.27	-0.4	-0.44	0.17
2-Chloroethanol	2.98	2.85	2.84	0.14
2-Chloronaphthalene	0.58	0.36	0.33	0.25
2-Chloronitrobenzene	0.74	0.82	0.83	-0.09
2-Chloropentane	3.81	3.72	3.73	0.08
2-Chloropropane	4.83	4.66	4.66	0.17
2-Chloropropanoic acid	1.55	1.8	1.83	-0.28
2-Chloropropene	5.02	4.77	4.84	0.18
2-Chloropyridine	2.47	2.34	2.12	0.35
2-Chlorosyringaldehyde	-1.1	-1.09	-1.17	0.07
2-Chlorotoluene	2.67	2.6	2.59	0.08
2-cis-Decene	2.23	2.32	2.33	-0.1
2-cis-Dodecene	1.21	1.38	1.39	-0.18
2-Decanol	0.54	0.68	0.69	-0.15
2-Diethylaminoethanethiol	2.47	2.42	2.43	0.04
2-Difluoromethoxy-1,1,1,3,3,3-hexafluoropropane	4.3	4.55	4.86	-0.56
2-Difluoromethoxy-1,1-difluoroethane	4.48	4.55	4.5	-0.02
2-Diisopropylaminoethanethiol	1.79	2.1	2.2	-0.41
2-Dimethylaminoethanethiol	3.21	3.24	3.22	-0.01
2-Dodecanol	-0.44	-0.26	-0.25	-0.19
2-Ethoxy-1,1,1,3,3,3-hexafluoro-2-trifluoromethylpropane	4.3	3.84	3.72	0.58
2-Ethoxyethanol	2.87	2.67	2.64	0.23
2-Ethoxyethylacetate	2.51	2.79	2.78	-0.27
2-Ethyl-1-butanol	2.31	2.3	2.3	0.01
2-Ethyl-1-butene	4.37	4.37	4.41	-0.04
2-Ethyl-1-hexanol	1.28	1.36	1.36	-0.08
2-Ethyl-1-pentene	3.88	3.9	3.92	-0.04
2-Ethylfuran	3.89	3.35	3.34	0.55
2-Ethylhexyl acetate	1.51	1.48	1.48	0.03
2-Ethylhexyl acrylate	1.39	1.18	1.18	0.21
2-Ethyl-m-xylene	2.13	2.25	2.25	-0.12
2-Ethylnaphthalene	0.41	0.27	0.23	0.18
2-Ethylnitrobenzene	0.91	0.73	0.75	0.16
2-Ethyl-p-xylene	2.1	2.25	2.26	-0.16
2-Ethylpyridine	2.82	2.55	2.5	0.32
2-Ethylthiophene	3.06	3.03	3.03	0.03
2-Ethyltoluene	2.53	2.51	2.54	-0.01
2-Fluorenylaldehyde	-3.08	-2.87	-2.89	-0.19

2-Fluoroiodobenzene	2.1	2.27	2.27	-0.17
2-Fluoronitrobenzene	1.31	1.56	1.57	-0.26
2-Heptanol	2	2.09	2.08	-0.08
2-Heptanone	2.71	2.75	2.76	-0.05
2-Hexanol	2.52	2.56	2.54	-0.02
2-Hexyne	4.03	3.96	3.94	0.09
2-Hydroxy-4-methoxybenzaldehyde	0.03	0	-0.02	0.05
2-Hydroxy-5-methoxybenzaldehyde	0.31	-0.03	-0.06	0.37
2-Hydroxy-6-methoxybenzaldehyde	-0.06	0	0.01	-0.07
2-Imidazolidinone	-2.46	-2.33		-0.13
2-Iodofluorene	-2.39	-2.9	-2.98	0.59
2-Isopropylnaphthalene	-0.14	0.06	0.02	-0.16
2-Isopropyltoluene	2.32	2.3	2.28	0.04
2-Methoxyaniline	1.05	1.1	1.09	-0.04
2-Methoxyethylamine	3.83	3.7	3.67	0.16
2-Methoxy-p-cresol	0.5	1.04	1.07	-0.57
2-Methoxyphenol	1.14	1.3	1.3	-0.16
2-Methyl-1,1-diphenylpropane	-0.55	-0.76	-0.78	0.23
2-Methyl-1,2-propanediamine	3.12	3.15	3.18	-0.06
2-Methyl-1-butanol	2.62	2.77	2.77	-0.15
2-Methyl-1-heptene	3.44	3.43	3.45	-0.01
2-Methyl-1-hexene	3.91	3.9	3.89	0.02
2-Methyl-1-octene	2.94	2.96	2.99	-0.05
2-Methyl-1-pentanol	2.38	2.3	2.3	0.08
2-Methyl-1-pentene	4.41	4.37	4.35	0.06
2-Methyl-1-propanethiol	3.97	3.85	3.84	0.13
2-Methyl-1-propanol	3.21	3.24	3.25	-0.04
2-Methyl-2-butanol	3.35	3.3	3.31	0.04
2-Methyl-2-butenenitrile	3.53	3.67	3.67	-0.14
2-Methyl-2-pentanol	2.93	2.83	2.78	0.15
2-Methyl-2-pentene	4.32	4.4	4.39	-0.07
2-Methyl-2-propanol	3.74	3.77	3.8	-0.06
2-Methyl-3-butenenitrile	3.44	3.67	3.69	-0.25
2-Methyl-3-pentanol	2.86	2.82	2.84	0.02
2-Methylacrylonitrile	3.98	4.11	4.07	-0.09
2-Methylaminoethanol	2.09	2.54	2.6	-0.51
2-Methylanthracene	-1.78	-1.76	-1.82	0.04
2-Methylaziridine	4.19	4.25	4.26	-0.07
2-Methylbenzaldehyde	1.8	1.94	1.9	-0.1
2-Methyldecane	1.92	1.91	1.92	0
2-Methylglutaric acid	-3	-3.22	-3.23	0.23
2-Methylheptane	3.44	3.32	3.32	0.12

2-Methylhexane	3.91	3.79	3.82	0.09
2-Methylnaphthalene	0.86	0.74	0.71	0.15
2-Methylnonane	2.4	2.38	2.38	0.02
2-Methyloctane	2.93	2.85	2.88	0.05
2-Methylpentane	4.45	4.26	4.28	0.17
2-Methylpropanal	4.36	4.16	4.16	0.2
2-Methylpropane	5.38	5.2	5.22	0.16
2-Methylpropanoic acid	2.41	2.34	2.3	0.11
2-Methylpropylamine	4.27	3.99	3.99	0.28
2-Methylpyrazine	3.04	2.75	2.71	0.33
2-Methylstyrene	2.38	2.68	2.66	-0.28
2-Methyltetrahydrofuran	4.37	4.34	4.32	0.05
2-Methylthiophene	3.52	3.5	3.49	0.03
2-Nitroaniline	-1.14	-0.49	-0.39	-0.75
2-Nitrobutane	3.02	2.9		0.12
2-Nitro-m-xylene	1.24	0.94	0.96	0.28
2-Nitropropane	3.35	3.37		-0.02
2-Nitro-t-butylbenzene	0.68	0.2	0.19	0.49
2-Nitrotoluene	1.29	1.2	1.19	0.1
2-Nonanol	1.03	1.15	1.17	-0.14
2-Nonanone	1.78	1.81	1.8	-0.02
2-n-Propylphenol	1.07	0.55	0.56	0.51
2-n-Propylthiophene	2.62	2.56	2.58	0.04
2-Octanol	1.53	1.62	1.61	-0.08
2-Octanone	2.26	2.28	2.29	-0.03
2-Pentanol	2.92	3.03	3.01	-0.09
2-Pentyne	4.5	4.43	4.41	0.09
2-Phenylethylamine	1.63	1.56	1.55	0.08
2-Picoline	3.18	3.02	2.99	0.19
2-Propanethiol	4.57	4.37	4.36	0.21
2-Propanol	3.8	3.97	3.98	-0.18
2-Propyltoluene	2.12	2.04	2.03	0.09
2-Propyn-1-ol	3.34	3.65	3.63	-0.29
2-Pyrrolidone	0.45	0.37	0.33	0.12
2-t-Butyl-4-methylphenol	0.37	0.23	0.23	0.14
2-t-Butyl-5-methylphenol	0.56	0.23	0.24	0.32
2-t-Butylanthracene	-2.86	-2.76	-2.79	-0.07
2-t-Butylphenol	1.04	0.49	0.49	0.55
2-Tetradecanol	-1.52	-1.2	-1.18	-0.34
2-t-Pentylphenol	0.69	0.02	0.02	0.67
2-Undecanol	0.1	0.21	0.23	-0.14
2-Undecanone	0.75	0.87	0.87	-0.12

3-(Difluoromethoxy)-1,1,1,2,2-pentafluoropropane	4.65	4.49	4.45	0.2
3-(Perfluorobutyl)propanol	1.95	1.59	1.59	0.36
3,3,3-Trifluoropropene	5.76	5.67	5.68	0.08
3,3',4,4',5-Pentachlorobiphenyl	-3.31	-2.6	-2.63	-0.68
3,3',4,4',5-Pentachlorodiphenyl ether	-3.25	-2.96	-2.97	-0.28
3,3',4,4'-Tetrabromodiphenyl ether	-3.92	-3.48	-3.48	-0.44
3,3',4,4'-Tetrachlorobiphenyl	-2.66	-1.96	-1.97	-0.69
3,3',4,4'-Tetrachlorodiphenyl ether	-2.59	-2.32	-2.36	-0.23
3,3',4,5,5'-Pentachlorobiphenyl	-3.11	-2.6	-2.65	-0.46
3,3',4,5'-Tetrachlorobiphenyl	-2.47	-1.96	-1.95	-0.52
3,3',4,5-Tetrachlorobiphenyl	-2.5	-1.96	-1.97	-0.53
3,3',4-Tribromodiphenyl ether	-2.94	-2.55	-2.59	-0.35
3,3',4-Trichlorobiphenyl	-1.89	-1.32	-1.32	-0.57
3,3',5,5'-Tetrachlorobiphenyl	-2.26	-1.96	-1.99	-0.27
3,3',5-Trichlorobiphenyl	-1.72	-1.32	-1.34	-0.38
3,3,5-Trimethylheptane	2.75	2.79	2.83	-0.08
3,3'-Bitolyl	-0.39	0.08	0.09	-0.48
3,3'-Dichlorobiphenyl	-1.05	-0.68	-0.7	-0.35
3,3-Dimethyl-1-butene	4.76	4.58	4.6	0.16
3,3-Dimethyl-1-pentene	4.14	4.11	4.12	0.02
3,3-Dimethylhexane	3.58	3.47	3.47	0.11
3,3-Dimethylpentane	4.04	3.94	3.95	0.09
3,4,4',5-Tetrachlorobiphenyl	-2.54	-1.96	-1.95	-0.59
3,4,4'-Tribromodiphenyl ether	-3.1	-2.55	-2.61	-0.49
3,4,4'-Trichlorobiphenyl	-1.9	-1.32	-1.34	-0.56
3,4,5,6-Tetrachloroguaiacol	-0.86	-1.26	-1.27	0.41
3,4,5-Trichlorobiphenyl	-1.98	-1.32	-1.32	-0.66
3,4',5-Trichlorobiphenyl	-1.69	-1.32	-1.34	-0.35
3,4,5-Trichloroguaiacol	-0.19	-0.62	-0.63	0.44
3,4'-Dibromodiphenyl ether	-2	-1.62	-1.66	-0.34
3,4-Dibromodiphenyl ether	-1.97	-1.62	-1.63	-0.34
3,4-Dichloroaniline	0.23	0.27	0.27	-0.04
3,4'-Dichlorobiphenyl	-1.23	-0.68	-0.73	-0.5
3,4-Dichlorobiphenyl	-1.27	-0.68	-0.69	-0.58
3,4-Dichloronitrobenzene	0.15	0.18	0.17	-0.02
3,4-Dichlorophenol	0.38	0.47	0.49	-0.11
3,4-Dichlorotoluene	1.75	1.96	1.95	-0.2
3,4-Dimethylhexane	3.46	3.58	3.61	-0.15
3,4-Lutidine	2.35	2.71	2.79	-0.44
3,4-Xylidine	0.56	1.03	1.06	-0.5
3,5,5-Trimethyl-2-cyclohexenone	1.7	1.06	1.05	0.65
3,5-Dichlorobiphenyl	-0.9	-0.68	-0.71	-0.19

3,5-Dichlorosyringol	-0.33	-0.43	-0.41	0.08
3,5-Lutidine	2.36	2.71	2.74	-0.38
3-Bromochlorobenzene	2	1.93	1.92	0.08
3-Bromodiphenyl ether	-0.9	-0.69	-0.79	-0.11
3-Bromopyridine	2.39	2.3	2.25	0.14
3-Bromotoluene	2.17	2.31	2.32	-0.15
3-Chloroaniline	1.1	0.91	0.88	0.22
3-Chlorobiphenyl	0	-0.04	-0.06	0.06
3-Chloronitrobenzene	0.8	0.82	0.8	0
3-Chlorophenol	1.22	1.11	1.09	0.13
3-Chlorostyrene	2.13	2.3	2.31	-0.19
3-Chlorosyringol	-0.08	0.21	0.23	-0.31
3-Chlorotoluene	2.69	2.6	2.59	0.1
3-Difluoromethoxy-1,1,2,2-tetrafluoropropane	4.12	4.18	4.16	-0.04
3-Ethyl-1-pentene	4.04	3.96	3.95	0.08
3-Ethyl-2-methylpentane	3.5	3.58	3.6	-0.1
3-Ethyl-3-methylpentane	3.49	3.47	3.49	0
3-Ethylheptane	3.32	2.85	2.87	0.45
3-Ethylhexane	3.43	3.32	3.35	0.08
3-Ethyl-o-xylene	1.94	2.25	2.28	-0.34
3-Ethylpentane	3.89	3.79	3.81	0.08
3-Ethylphenol	0.84	1.02	1.02	-0.18
3-Ethylpyridine	2.52	2.5	2.51	0.01
3-Ethyltoluene	2.63	2.51	2.5	0.13
3-Fluorobenzotrifluoride	3.71	3.43	3.43	0.28
3-Fluoroiodobenzene	2.19	2.27	2.26	-0.07
3-Fluoronitrobenzene	1.76	1.56	1.58	0.18
3-Heptanol	2	2.09	2.11	-0.11
3-Heptanone	2.56	2.75	2.76	-0.2
3-Hexanol	2.57	2.56	2.58	-0.01
3-Hexanone	3.27	3.22	3.23	0.04
3-Hexyne	4.07	3.96	4.01	0.06
3-Hydroxybenzaldehyde	0.26	0.45	0.45	-0.19
3-Isopropyltoluene	2.31	2.3	2.3	0.01
3-Methoxysalicylaldehyde	-0.04	0	0.01	-0.05
3-Methyl-1,2-butadiene	4.76	4.81		-0.05
3-Methyl-1-butene	5.07	4.9	4.92	0.15
3-Methyl-1-butyne	4.94	4.93	4.94	0
3-Methyl-1-hexene	4.04	3.96	3.95	0.09
3-Methyl-1-pentene	4.55	4.43	4.44	0.11
3-Methyl-2-butanol	3.09	3.29	3.31	-0.22
3-Methyl-2-butanone	3.85	3.95	3.96	-0.11

3-Methyl-2-butenyl acetate	2.67	3.03	3.03	-0.36
3-Methyl-2-pentanol	2.64	2.82	2.84	-0.2
3-Methyl-3-pentanol	2.87	2.83	2.84	0.03
3-Methylbenzaldehyde	1.65	1.94	1.91	-0.26
3-Methylbutanoic acid	1.79	1.87	1.87	-0.08
3-Methylcholanthrene	-5.83	-6.44	-6.52	0.69
3-Methyl-cis-2-pentene	4.31	4.4	4.4	-0.09
3-Methylcyclopentene	3.41	3.26	3.24	0.17
3-Methyleneheptane	3.43	3.43	3.46	-0.03
3-Methylglutaric acid	-3.04	-3.22	-3.23	0.19
3-Methylheptane	3.41	3.32	3.33	0.08
3-Methylhexane	3.91	3.79	3.81	0.1
3-Methylnonane	2.42	2.38	2.41	0.01
3-Methyloctane	2.94	2.85	2.87	0.07
3-Methylpentane	4.4	4.26	4.26	0.14
3-Methylpyridine	2.9	2.97	3.04	-0.14
3-Methylstyrene	2.4	2.68	2.7	-0.3
3-Methylthiophene	3.48	3.63	3.69	-0.21
3-Methyl-trans-2-pentene	4.27	4.4	4.43	-0.16
3-Methylundecane	1.41	1.44	1.44	-0.03
3-Nitrobenzotrifluoride	1.54	1.29	1.26	0.27
3-Nitrotoluene	1.15	1.2	1.2	-0.05
3-Nonanol	1.18	1.15	1.17	0.01
3-Octanol	1.64	1.62	1.64	0
3-Octanone	2.46	2.28	2.29	0.17
3-Pentanol	3.06	3.03	3.04	0.02
3-Propyltoluene	2.18	2.04	2.05	0.13
3-t-Butylphenol	0.07	0.49	0.5	-0.43
4,4,6-Trimethyl-1,3-dioxane	2.91	2.78	2.73	0.18
4,4'-Dibromodiphenyl ether	-2.06	-1.62	-1.61	-0.45
4,4'-Dichlorobiphenyl	-1.14	-0.68	-0.68	-0.46
4,4-Dimethyl-1-pentene	4.26	4.11	4.11	0.15
4,5,6-Trichloroguaiacol	-0.6	-0.62	-0.61	0.01
4,5-Dichloroguaiacol	0.19	0.02	0.01	0.18
4,5-Dimethylphenanthrene	-1.91	-2.02	-2.07	0.16
4-Acetoxyacetophenone	-0.13	0	-0.19	0.07
4-Allyl-2-methoxyphenol	0.23	0.27	0.26	-0.03
4-Amino-3,5,6-trichloro-2-pyridinecarboxlic acid	-4.08	-3.96	-3.74	-0.34
4-Bromodiphenyl ether	-0.59	-0.69	-0.73	0.14
4-Bromophenol	0.56	0.82	0.8	-0.24
4-Bromotoluene	2.18	2.31	2.31	-0.13
4-Chloroaniline	0.55	0.91	0.97	-0.42

4-Chlorobiphenyl	-0.04	-0.04	-0.06	0.02
4-Chloroiodobenzene	1.21	1.53	1.54	-0.33
4-Chloro-m-cresol	0.82	0.85	0.83	-0.01
4-Chloronitrobenzene	0.46	0.82	0.81	-0.35
4-Chlorophenol	1.07	1.11	1.13	-0.06
4-Chlorotoluene	2.57	2.6	2.59	-0.02
4-Cumylphenyl diphenyl phosphate	-8.76	-8.8	-8.67	-0.09
4-Ethyl-m-xylene	2.07	2.25	2.28	-0.21
4-Ethyl-o-xylene	2.14	2.25	2.3	-0.16
4-Ethylphenol	0.7	1.02	1.01	-0.32
4-Ethylpyridine	2.48	2.5	2.51	-0.03
4-Ethyltoluene	2.6	2.51	2.5	0.1
4-Fluoroiodobenzene	2.22	2.27	2.28	-0.06
4-Fluoronitrobenzene	1.65	1.56	1.55	0.1
4-Fluorophenol	1.71	1.85	1.88	-0.17
4-Heptanol	2.11	2.09	2.1	0.01
4-Heptanone	2.22	2.75	2.76	-0.54
4-Iodophenol	0.33	0.42	0.4	-0.07
4-Methyl-1-hexene	4	3.96	3.95	0.05
4-Methyl-1-pentanol	2.05	2.3	2.29	-0.24
4-Methyl-1-pentene	4.56	4.43	4.44	0.12
4-Methyl-2-pentanol	2.84	2.82	2.84	0
4-Methylbenzaldehyde	1.54	1.94	1.91	-0.37
4-Methyl-cis-2-pentene	4.51	4.46	4.49	0.02
4-Methylcyclopentene	3.43	3.26	3.27	0.16
4-Methylheptane	3.44	3.32	3.34	0.1
4-Methylnonane	2.49	2.38	2.38	0.11
4-Methyloctane	2.96	2.85	2.85	0.11
4-Methylpent-3-en-2-one	3.17	3.62	3.66	-0.49
4-Methylpyridine	2.9	2.97	2.98	-0.08
4-Methylstyrene	2.39	2.68	2.7	-0.31
4-Methyl-trans-2-pentene	4.47	4.46	4.44	0.03
4-Nitrotoluene	1.36	1.2	1.19	0.17
4-Octylphenol	-1.15	-1.8	-1.79	0.64
4-Propylphenol	0.88	0.55	0.51	0.37
4-Propyltoluene	2.17	2.04	2.01	0.16
4-t-Butylphenol	0.56	0.49	0.51	0.05
5-Ethyl-m-xylene	1.87	2.25	2.3	-0.43
5-Isopropyl-2-methylphenol	0.55	0.55	0.59	-0.04
5-Methyl-1-hexene	4.02	3.96	3.95	0.07
5-Methylnonane	2.47	2.38	2.4	0.07
5-Nonanone	1.89	1.81	1.82	0.07

6-Methyl-1-heptene	3.1	3.49	3.5	-0.4
7,12-Dimethylbenz[a]anthracene	-5.2	-4.52	-4.61	-0.58
9,10-Benzophenanthrene	-3.89	-4	-4.07	0.18
9,10-Dihydroanthracene	-1.48	-2.16	-2.17	0.69
9,10-Diphenylanthracene	-7.73	-7.3	-7.35	-0.38
Acenaphthene	-0.49	-1.18	-1.23	0.74
Acenaphthylene	-0.9	-1.56	-1.58	0.68
Acetaldehyde	5.08	4.84	4.85	0.23
Acetamide	1.99	2.3	2.37	-0.38
Acetic acid	3.21	3.02	3.02	0.19
Acetol	2.75	2.67	2.65	0.1
Acetonitrile	4.08	4.68	4.69	-0.61
Acetophenone	1.72	1.99	1.98	-0.26
Acetylacetone	3.01	2.91	2.92	0.09
Acetylchloride	4.86	4.75	4.74	0.12
Acrylic acid	2.76	2.72	2.69	0.07
Adamantane	1.26	1.52	1.45	-0.19
Adiponitrile	2.1	1.6	1.51	0.58
Allyl acetate	3.67	3.74	3.74	-0.07
Allyl alcohol	3.54	3.62	3.61	-0.07
Allyl chloride	4.69	4.51	4.52	0.17
Allyl cyanide	3.39	3.91	3.94	-0.55
Allyl hexanoate	1.91	1.86	1.86	0.05
Allyl sulfide	3.12	3.16	3.17	-0.05
Allylamine	4.53	4.37	4.36	0.17
alpha,alpha-Dimethylphenethylamine	1.51	1.25	1.15	0.35
alpha-Curcumene	0.04	0.35	0.33	-0.29
alpha-Ionone	0.33	-0.02	0.02	0.31
alpha-Methyl benzylamine	1.92	1.87	1.84	0.08
alpha-Methylstyrene	2.47	2.67	2.65	-0.18
alpha-Pinene	2.77	2.45	2.5	0.27
Alverine	-2.85	-3.39	-3.42	0.57
Ametryn	-3.44	-3.54		0.1
Amyl acetate	2.66	2.63	2.65	0.01
Amyl propionate	2.68	2.16	2.19	0.49
Amylamine	3.32	3.26	3.26	0.06
Amylbenzene	1.65	1.36	1.35	0.3
Aniline	1.96	1.55	1.56	0.4
Anthracene	-1.18	-1.5	-1.5	0.32
Arachic alcohol	-4.38	-4.54	-4.56	0.18
Atrazine	-4.4	-4.32		-0.08
Azepane	3.08	3.29	3.33	-0.25

Azetidine	4.37	4.36	4.36	0.01
Azidocyclopentane	3.15	3.13	3.01	0.14
Aziridine	4.47	4.41	4.38	0.09
Azocane	2.61	3.16	3.12	-0.51
Barban	-4.3	-4.32	-4.36	0.06
Benz[a]anthracene	-3.73	-4	-4.09	0.36
Benz[e]acephenanthrylene	-6	-6.22	-6.29	0.29
Benzalchloride	1.82	1.71	1.69	0.13
Benzaldehyde	2.23	2.2	2.18	0.05
Benzene	4.1	3.5	3.48	0.62
Benzo[a]fluorene	-3.96	-4.07	-4.05	0.09
Benzo[a]phenanthrene	-3.75	-4	-4.07	0.32
Benzo[a]pyrene	-6.14	-6.14	-6.21	0.07
Benzo[b]fluorene	-3.51	-4.07	-4.02	0.51
Benzo[b]naphtho[2,3-d]thiophene	-3.39	-3.36		-0.03
Benzo[b]triphenylene	-6.46	-6.5	-6.59	0.13
Benzo[e]pyrene	-6.1	-6.14	-6.21	0.11
Benzo[f]quinoline	-2.13	-1.93		-0.2
Benzo[ghi]perylene	-8	-8.28	-8.41	0.41
Benzonitrile	2	2.04	2.03	-0.03
Benzophenone	-0.88	-0.65	-0.66	-0.21
Benzotrifluoride	3.71	3.33	3.33	0.38
Benzoyl chloride	1.92	2.11	2.15	-0.23
Benzphetamine	-1.4	-1.73	-1.74	0.34
Benzyl acetate	1.37	1.4	1.4	-0.03
Benzyl alcohol	1.18	1.28	1.27	-0.09
Benzyl benzoate	-1.51	-1.24	-1.26	-0.25
Benzyl butyl phthalate	-4.21	-4.28	-4.37	0.16
Benzyl ethyl ether	2.09	1.99	1.94	0.15
Benzylamine	1.98	2.03	2.01	-0.03
beta-Citronellol	0.31	0.82	0.84	-0.53
beta-HCH	-0.58	-0.7	-0.75	0.18
beta-Phellandrene	2.32	1.65	1.62	0.7
beta-Sesquiphellandrene	0.18	-0.3	-0.33	0.51
Bicifadine	0.11	0.4	0.43	-0.32
Bicyclohexyl	1.16	1.3	1.24	-0.08
Bifenox	-5.27	-4.9	-4.94	-0.33
Biphenyl	0.63	0.6	0.61	0.02
Biphenylene	-0.43	-0.54	-0.49	0.06
Bis(2-chloroethyl) ether	2.32	2.49	2.51	-0.19
Bis(3-aminopropyl)amine	0.61	0.73	0.69	-0.08
Bis(perfluorobutyl)disulfide	2.56	2.4	2.33	0.23

Bistrifluoromethyl disulfide	4.85	4.8		0.05
Bromobenzene	2.73	2.57	2.56	0.17
Bromocyclohexane	2.61	2.67	2.66	-0.05
Bromoethane	4.6	4.54	4.54	0.06
Bromoethylene	5.13	4.8		0.33
Butanal	4.17	3.9	3.88	0.29
Butane	5.36	4.94	4.96	0.4
Butanedioic acid	-2.51	-2.54	-2.56	0.05
Butanoic acid	2.12	2.08	2.1	0.02
Butyl 1,1-dimethylpropyl ether	2.79	2.78	2.83	-0.04
Butyl 4-oxopentanoate	0.59	0.91	0.89	-0.3
Butyl butanoate	2.38	2.16	2.18	0.2
Butyl glycolate	1.65	1.14	1.14	0.51
Butyl isocyanate	3.46	3.45		0.01
Butyl lactate	1.67	1.19	1.18	0.49
Butyl propanoate	2.76	2.63	2.64	0.12
Butyl t-octyl ether	1.79	1.78	1.8	-0.01
Butyl-1,2-dinitrate	1.8	1.78	1.8	0
Butylacetamide	0.78	0.29	0.24	0.54
Butylbenzene	2.14	1.83	1.81	0.33
Butylcellosolve	2.09	1.73	1.72	0.37
Butylcyclohexane	2.24	2.18	2.16	0.08
Butylcyclopentane	2.69	2.91	2.91	-0.22
Butylmethylketone	3.21	3.22	3.22	-0.01
Butylsilane	4.51	4.5		0.01
Butyric acid methylester	3.63	3.38	3.41	0.22
Butyronitrile	3.42	3.74	3.79	-0.38
Camphene	2.7	2.68	2.68	0.02
Camphor	1.62	1.68	1.63	-0.01
Capraldehyde	1.13	1.08	1.08	0.05
Caproic aldehyde	3.17	2.96	2.95	0.22
Caprylene	3.36	3.23	3.23	0.13
Caprylic aldehyde	2.19	2.02	2.06	0.13
Carbitol	1.24	1.42	1.4	-0.16
Carbofuran	-4.14	-4.11	-4.08	-0.06
Carvone	1.04	0.81	0.89	0.15
Cedrol	-1.16	-0.88	-0.92	-0.23
Cetane	-0.72	-0.7	-0.68	-0.04
Cetene	-0.44	-0.53	-0.51	0.07
Chloral	3.84	3.27	3.22	0.62
Chlorfenvinphos	-3	-2.63	-2.46	-0.54
Chloroacetic acid	1.6	1.95	1.93	-0.33

Chloroacetic acid ethylester	2.81	2.97	2.98	-0.17
Chloroacetone	3.21	3.56	3.55	-0.34
Chloroacetyl chloride	3.52	3.68	3.73	-0.21
Chlorobenzene	3.2	2.86	2.84	0.36
Chlorocyclohexane	3.03	3.05	3.04	-0.01
Chloroethane	5.08	4.81	4.81	0.27
Chloroethylene	5.54	5.23	5.2	0.34
Chloropentafluorobenzene	3.38	3.36	3.34	0.04
Chloroprene	4.48	4.47	4.55	-0.07
Chloropropham	-2.88	-2.5	-2.29	-0.59
Chlorothalonil	-2.09	-1.98	-1.95	-0.14
Chlorpyrifos-methyl	-2.25	-2.26		0.01
Chlorpyriphos	-2.6	-2.82		0.22
Cinnamaldehyde	0.92	1.46	1.46	-0.54
cis 3-Hexenyl butyrate	1.41	1.42	1.43	-0.02
cis- Cyclooctene	3.01	2.72	2.71	0.3
cis-1,1,1,4,4,4-Hexafluoro-2-butene	4.87	5.32	5.33	-0.46
cis-1,2-Dibromoethylene	3.39	3.58		-0.19
cis-1,2-Dichloroethylene	4.44	4.44	4.39	0.05
cis-1,2-Dimethylcyclohexane	3.29	3.38	3.33	-0.04
cis-1,2-Dimethylcyclopentane	3.81	4.11	4.11	-0.3
cis-1,3-Dichloropropene	3.71	3.98	4.03	-0.32
cis-1,3-Dimethylcyclohexane	3.46	3.38	3.37	0.09
cis-1,3-Dimethylcyclopentane	4.07	4.11	4.09	-0.02
cis-1,3-Pentadiene	4.7	4.84	4.84	-0.14
cis-1,4-Dichloro-2-butene	2.74	3	3	-0.26
cis-1,4-Dimethylcyclohexane	3.38	3.38	3.37	0.01
cis-1,4-Hexadiene	4.37	4.37	4.37	0
cis-1-Phenyl-1-propene	2.31	2.5	2.52	-0.21
cis-2-Butene	5.33	5.14	5.13	0.2
cis-2-Heptene	3.81	3.73	3.74	0.07
cis-2-Hexene	4.3	4.2	4.2	0.1
cis-2-Methylcyclohexanol	2.3	2.15	2.15	0.15
cis-2-Octene	3.38	3.26	3.26	0.12
cis-2-Pentene	4.82	4.67	4.69	0.13
cis-3-Heptene	3.87	3.73	3.74	0.13
cis-3-Hexene	4.3	4.2	4.21	0.09
cis-3-Hexenyl 2-methylbutyrate	1.19	1.21	1.21	-0.02
cis-3-Hexenyl propionate	1.82	1.89	1.87	-0.05
cis-3-Octene	3.37	3.26	3.27	0.1
cis-4,6-Dimethyl-1,3-dioxane	2.9	2.99	3.07	-0.17
cis-4-Octene	3.38	3.26	3.26	0.12

cis-5-Octenyl propionate	0.85	0.95	0.98	-0.13
cis-Crotononitrile	3.63	3.94	3.99	-0.36
cis-Cyclohexane-1,2-dinitrate	0.74	0.54	0.51	0.23
cis-Cyclohexane-1,3-dinitrate	0.25	0.54	0.53	-0.28
cis-Decalin	2.04	2.4	2.31	-0.27
cis-Stilbene	-0.02	-0.14	-0.17	0.15
cis-Verbenol	0.72	1.01	1.01	-0.29
Citronellal	1.43	1.74	1.72	-0.29
Citronellyl acetate	0.6	0.94	0.98	-0.38
Coumarin	-0.4	-0.79	-0.81	0.42
Crotonaldehyde	3.72	4.1	4.11	-0.39
Cryofluorane	5.31	5.3	5.33	-0.02
Cumene	2.77	2.56	2.53	0.24
Cyanazine	-5.28	-5.67		0.39
Cyanoethylene	4.21	4.38	4.37	-0.16
Cyclobutane	5.2	4.74	4.71	0.49
Cyclobutanone	3.75	3.23	3.28	0.47
Cyclogeraniolane	3.17	3.06	3.08	0.09
Cycloheptane	3.46	3.67	3.63	-0.17
Cycloheptene	3.43	2.85	2.84	0.59
Cyclohexane	4.11	3.8	3.8	0.31
Cyclohexanethiol	2.75	2.76	2.72	0.02
Cyclohexanol	2.05	2.36	2.35	-0.3
Cyclohexanone	2.76	2.34	2.32	0.44
Cyclohexyl azide	2.52	2.4	2.32	0.2
Cyclohexylamine	3.15	2.9	2.89	0.26
Cyclohexylbenzene	0.74	0.95	0.89	-0.15
Cyclooctane	2.88	3.54	3.47	-0.59
Cyclopentane	4.62	4.53	4.51	0.11
Cyclopentanol	2.47	3.09	3.08	-0.61
Cyclopentanone	3.2	2.71	2.69	0.51
Cymperator	-6.72	-6.82	-6.87	0.15
DBCP	1.88	1.87	1.88	0
DDD	-3.01	-2.89	-2.9	-0.11
DDE	-2.59	-2.44	-2.46	-0.13
DDT	-3.24	-2.93	-2.95	-0.29
Decabromodiphenyl ether	-8.53	-9.06	-9.21	0.68
Decachlorodiphenyl ether	-5.8	-6.16	-6.2	0.4
Decafluorotetrahydrothiophene	3.98	4.07	4.21	-0.23
Decamethylcyclopentasiloxane	1.32	1.28	1.25	0.07
Decamethyltetrasiloxane	1.7	1.39		0.31
Decane	2.24	2.12	2.13	0.11

Decanedioic acid	-5.42	-5.36	-5.38	-0.04
Decyl-1,2-dinitrate	-0.96	-1.04	-1.05	0.09
Decylbenzene	-0.75	-0.99	-1	0.25
Decylcyclohexane	-0.72	-0.64	-0.68	-0.04
delta-HCH	-0.82	-0.7	-0.65	-0.17
Demeton	-0.88	-0.69	-0.69	-0.18
Deprenyl	0.4	0.64	0.66	-0.26
Devrinol	-3.28	-3.72	-3.79	0.51
Diallate	-1.7	-1.59	-1.56	-0.14
Diamylamine	1.58	1.27	1.33	0.25
Dibenz[ah]anthracene	-6.6	-6.5	-6.47	-0.13
Dibenzo[b,d]thiophene	-0.82	-0.86	-0.91	0.09
Dibenzo[b,e]pyridine	-2.19	-2.09		-0.1
Dibenzo-p-dioxin	-1.26	-1.94	-1.95	0.69
Dibenzylsulfide	-1.31	-1.52	-1.57	0.26
Dibutyl carbonate	1.41	1.38	1.37	0.04
Dibutyl decanedioate	-3.19	-3.44	-3.47	0.28
Dibutyl maleate	-1.06	-0.42	-0.37	-0.69
Dibutyl oxalate	0.26	0.32	0.32	-0.06
Dibutyl phthalate	-2.37	-2.58	-2.61	0.24
Dibutylacetamide	0.38	0.24	0.2	0.18
Dibutylamine	2.53	2.21	2.19	0.34
Dibutylsulfide	2.21	1.88	1.87	0.34
Dibutylsulfoxide	0.36	-0.15	-0.37	0.73
Dichlone	-3.48	-3.18	-3.03	-0.45
Dichloroacetic acid	1.38	1.49	1.53	-0.15
Diclofop-methyl	-4.23	-3.56	-3.52	-0.71
Dicofol	-4.27	-4.36	-4.47	0.2
Diethion	-3.8	-3.84		0.04
Diethyl disulfide	2.75	2.8	2.83	-0.08
Diethyl glutarate	0.55	0.79	0.8	-0.25
Diethyl malonate	1.57	1.73	1.78	-0.21
Diethyl methylphosphonate	1.75	1.78		-0.03
Diethyl oxalate	1.76	2.2	2.21	-0.45
Diethyl phthalate	-0.69	-0.7	-0.75	0.06
Diethyl succinate	1.01	1.26	1.27	-0.26
Diethyl sulphide	3.89	3.76	3.75	0.14
Diethylacetamide	1.9	2.12	2.22	-0.32
Diethylacetic acid	1.4	1.4	1.36	0.04
Diethylamine	4.49	4.09	4.09	0.4
Diethylcarbonate	3.18	3.26		-0.08
Diethyleneglycol monobutyl ether	0.48	0.48	0.47	0.01

Diethylketone	3.7	3.69	3.7	0
Diethylsulfoxide	1.31	1.73	1.88	-0.58
Diheptylamine	-0.29	-0.61	-0.59	0.3
Dihexyl phthalate	-3.94	-4.46	-4.48	0.54
Dihexyl sulfide	0.02	0	0.02	0
Dihexylacetamide	-1.39	-1.64	-1.68	0.29
Dihexyladipate	-3.4	-3.44	-3.43	0.03
Dihexylamine	0.65	0.33	0.33	0.32
Dihydropinene	2.58	3.21	3.31	-0.73
Diisobutyl ether	3.34	3.27	3.28	0.06
Diisobutyl phthalate	-2	-2.06	-2.06	0.06
Diisobutylamine	2.99	2.77	2.73	0.26
Diisobutylene	3.79	3.84	3.84	-0.05
Diisopropyl ether	4.3	4.17	4.2	0.1
Diisopropyl methylphosphonate	1.66	1.32		0.33
Diisopropyl sulfide	3.41	3.44	3.6	-0.19
Diisopropylamine	4.02	3.77	3.87	0.15
Diisopropylsulfoxide	1.27	1.41	1.51	-0.25
Dimethoate	-2.94	-2.97	-3.03	0.09
Dimethyl carbonate	3.85	3.82	3.8	0.05
Dimethyl disulfide	3.6	3.78		-0.18
Dimethyl hexanedioate	0.61	0.88	0.88	-0.27
Dimethyl oxalate	2.33	2.76	2.76	-0.43
Dimethyl phthalate	-0.38	-0.14	-0.13	-0.25
Dimethyl sulfide	4.82	4.74	4.73	0.09
Dimethylacetal	4.38	4.52	4.52	-0.14
Dimethylacetamide	2.41	2.94	3.04	-0.63
Dimethylamine	5.3	4.91	4.85	0.45
Dimethylether	5.77	5.19	5.14	0.63
Di-n-butylether	2.92	2.75	2.77	0.15
Di-n-propyl phthalate	-1.76	-1.64	-1.67	-0.09
Dioctyl sulfide	-1.52	-1.88	-1.9	0.38
Dioctylamine	-1.22	-1.55	-1.57	0.35
Dipentyl phthalate	-3.25	-3.52	-3.57	0.32
Diphenyl ether	0.43	0.24	0.12	0.31
Diphenylmethane	0.29	0.13	0.08	0.21
Dipropyl carbonate	2.32	2.32		0
Dipropyl ether	3.92	3.69	3.71	0.21
Dipropyl malonate	0.77	0.79	0.77	-0.01
Dipropyl oxalate	1.14	1.26	1.29	-0.15
Dipropyl sulfide	2.95	2.82	2.84	0.11
Dipropylamine	3.42	3.15	3.13	0.29

Dipropylsulfoxide	0.88	0.79	0.75	0.13
Di-s-butylsulfide	2.52	2.5	2.66	-0.14
Disulfoton	-1.62	-1.41	-1.32	-0.3
Diuron	-3.6	-3.78		0.18
Docosane	-3.67	-3.52	-3.49	-0.18
Dodecafluorotetrahydrothiophene	4.4	3.96		0.44
Dodecahydrosqualene	-5.82	-5.72	-5.72	-0.1
Dodecamethylcyclohexasiloxane	0.48	0.62	0.53	-0.05
Dodecane	1.2	1.18	1.21	-0.01
Dodecyl acetate	-0.5	-0.66	-0.66	0.16
Dopentacontane	-17.71	-17.62	-17.6	-0.11
Dotetracontane	-13.19	-12.92	-12.91	-0.28
Dotriacontane	-8.44	-8.22	-8.19	-0.25
d-Verbenone	1.19	1.21	1.24	-0.05
Eicosane	-2.68	-2.58	-2.55	-0.13
Endrin	-2.28	-2.03		-0.25
Enilconazole	-3.8	-3.92	-4.04	0.24
Epichlorohydrin	3.38	3.65	3.64	-0.26
Ethalfluralin	-3.65	-3.45	-3.42	-0.23
Ethanediol diacetate	1.48	2.2	2.25	-0.77
Ethanethiol	4.58	4.53	4.53	0.05
Ethanol	3.9	3.92	3.92	-0.02
Ethene	6.4	6.02	6.02	0.38
Ethyl 2-methylbutanoate	3.55	2.89	2.89	0.66
Ethyl 2-methylpentanoate	2.61	2.42	2.43	0.18
Ethyl acetate	4.09	4.04	4.05	0.04
Ethyl acetoacetate	1.98	2.32	2.34	-0.36
Ethyl acrylate	3.73	3.74	3.74	-0.01
Ethyl benzoate	1.37	1.4	1.4	-0.03
Ethyl butanoate	3.23	3.1	3.11	0.12
Ethyl decanoate	0.38	0.28	0.29	0.09
Ethyl ether	4.85	4.63	4.63	0.22
Ethyl formate	4.53	4.53	4.53	0
Ethyl glycolate	2.35	2.08	2.06	0.29
Ethyl hexanoate	2.2	2.16	2.18	0.02
Ethyl iodide	4.04	4.07	4.07	-0.03
Ethyl isobutanoate	3.53	3.36	3.37	0.16
Ethyl isovalerate	3.06	2.89	2.91	0.15
Ethyl lactate	2.44	2.13	2.09	0.35
Ethyl levulinate	1.4	1.85	1.87	-0.47
Ethyl perfluorooctanoate	2.11	1.91	1.86	0.25
Ethyl propanoate	3.67	3.57	3.59	0.08

Ethyl t-amyl ether	3.77	3.72	3.72	0.05
Ethyl t-butyl ether	4.21	4.19	4.21	0
Ethyl t-octyl ether	2.71	2.72	2.77	-0.06
Ethyl trans-cinnamate	0.18	0.66	0.65	-0.47
Ethylacetamide	1	1.23	1.28	-0.28
Ethylamine	4.95	4.67	4.68	0.27
Ethylbenzene	3.1	2.77	2.78	0.32
Ethylcarbamate	1.31	1.36	1.53	-0.22
Ethylcyclohexane	3.23	3.12	3.09	0.14
Ethylcyclopentane	3.73	3.37	3.39	0.34
Ethylenedichloride	4.02	3.74	3.74	0.28
Ethylepoxide	4.4	4.25	4.19	0.21
Ethylisopropylketone	3.38	3.48	3.48	-0.1
Ethylmethylether	5.3	4.91	4.88	0.42
Ethylnitrate	3.94	4.01	4.01	-0.07
Ethylnitrite	5.13	5.06	5.08	0.05
Ethyl-n-propylether	4.39	4.16	4.15	0.24
Ethyl-t-butylsulfide	3.42	3.35	3.35	0.06
Ethyne	6.59	6.32	6.25	0.34
Ethynylbenzene	3.03	2.97	2.94	0.09
Fenchlorphos	-2.15	-2.04	-2.11	-0.04
Fenchone	2.01	1.73	1.69	0.32
Fenitrothion	-2.5	-2.42	-2.4	-0.1
Fenoprop	-2.64	-2.24	-2.22	-0.42
Fenoxycarb	-5.77	-6.08	-6.2	0.43
Fenpropidin	-1.8	-1.95	-1.95	0.15
Fluorene	-1.18	-1.57	-1.52	0.34
Fluorobenzene	4.01	3.6	3.61	0.4
Fluoroethane	5.94	5.69	5.67	0.27
Fluoroethylene	6.44	6.04		0.4
Formic acid	3.71	3.51	3.43	0.28
Freon 113	4.65	4.63	4.62	0.03
Furfural	2.49	2.78	2.77	-0.28
Furfuranol	1.99	1.86	1.8	0.19
gamma-Bisabolene	-0.14	-0.73	-0.87	0.73
Glycerol formal	1.52	1.75	1.69	-0.17
Glyceryltriacetate	-0.46	0.13	0.16	-0.62
Guajen	0.27	0.48	0.48	-0.21
Hemimellitene	2.36	2.72	2.76	-0.4
Heneicosane	-3.18	-3.05	-3.04	-0.14
Heptachlor epoxide	-3.46	-3.62		0.16
Heptacosane	-5.95	-5.87	-5.85	-0.1

Heptadecane	-1.2	-1.17	-1.18	-0.02
Heptafluoropropyl trifluoromethyl ether	5.3	5.34	5.29	0
Heptane	3.76	3.53	3.54	0.22
Heptanedioic acid	-3.95	-3.95	-3.94	-0.01
Heptanonitrile	2.26	2.33	2.35	-0.09
Heptatriacontane	-10.82	-10.57	-10.54	-0.27
Heptyl bromide	2.22	2.19	2.23	-0.01
Heptyl butyrate	0.89	0.75	0.76	0.13
Heptylbenzene	0.74	0.42	0.43	0.31
Hexachlorobenzene	-0.52	-0.34	-0.34	-0.18
Hexachlorobutadiene	1.49	1.28	1.07	0.42
Hexacosane	-5.55	-5.4	-5.4	-0.15
Hexadecylamine	-1.96	-1.91	-1.9	-0.06
Hexafluorobenzene	4.07	4.1	4.09	-0.02
Hexamethyldisiloxane	3.75	4.07		-0.32
Hexamethyleneglycol	-1.16	-0.66	-0.68	-0.48
Hexane	4.3	4	4.02	0.28
Hexane-1,5-dinitrate	0.72	0.5	0.5	0.22
Hexane-2,5-dinitrate	0.84	0.74	0.68	0.16
Hexanenitrile	2.6	2.8	2.78	-0.18
Hexapentacontane	-19.36	-19.5	-19.47	0.11
Hexatetracontane	-15.06	-14.8	-14.77	-0.29
Hexatriacontane	-10.36	-10.1	-10.07	-0.29
Hexyl bromide	2.72	2.66	2.66	0.06
Hexyl glycolate	0.66	0.2	0.2	0.46
Hexyl hexanoate	0.5	0.28	0.3	0.2
Hexyl t-octyl ether	0.84	0.84	0.82	0.02
Hexyl-1,2-dinitrate	0.92	0.84	0.86	0.06
Hexylbenzene	1.17	0.89	0.88	0.29
Hydrogenperoxide	2.48	2.4		0.08
Icosafluorononane	3.11	3.47	3.51	-0.4
Indeno[1,2,3-cd]pyrene	-7.99	-8.36	-8.35	0.36
Iodobenzene	2.14	2.17	2.18	-0.04
Isoamyl acetate	2.88	2.89	2.92	-0.04
Isoamyl isobutyrate	2.26	2.21	2.22	0.03
Isobutyl acetate	3.38	3.36	3.38	0
Isobutyl acrylate	3.05	3.06	3.05	0
Isobutyl butanoate	2.7	2.42	2.44	0.26
Isobutyl chloride	4.28	4.13	4.14	0.14
Isobutyl formate	3.73	3.85	3.86	-0.13
Isobutyl isobutyrate	2.75	2.68	2.71	0.04
Isobutyl t-butylether	3.5	3.51	3.49	0.01

Isobutylbenzene	2.4	2.09	2.08	0.32
Isobutylene	5.48	5.31	5.3	0.18
Isobutylmethylether	4.44	4.23	4.24	0.2
iso-Butylsilane	4.63	4.76		-0.13
Isobutyronitrile	3.66	4	4.03	-0.37
Isocrotonic acid	1.91	2.28	2.3	-0.39
Isocumene	2.65	2.3	2.3	0.35
Isooctane	3.82	3.73	3.77	0.05
Isopentane	4.94	4.73	4.75	0.19
Isopentene	4.91	4.84	4.87	0.04
Isopentyl alcohol	2.5	2.77	2.78	-0.28
Isopentyl bromide	3.66	3.39	3.38	0.28
Isopentyl formate	3.3	3.38	3.39	-0.09
Isophytol	-3	-2.8	-2.86	-0.14
Isoprene	4.88	5.01	5.06	-0.18
Isopropyl formate	4.26	4.3	4.3	-0.04
Isopropyl lactate	2.38	1.9	1.94	0.44
Isopropyl methyl ether	4.9	4.68	4.7	0.2
iso-Propyl nitrite	4.78	4.83	4.85	-0.07
Isopropyl propyl ether	4.06	3.93	3.94	0.12
Isopropylacetate	3.9	3.81	3.82	0.08
Isopropylamine	4.88	4.51	4.51	0.37
Isopropylcyclohexane	2.8	2.91	2.89	-0.09
Isopropylcyclopentane	3.33	3.64	3.63	-0.3
Isopulegol	1.35	1.37	1.33	0.02
Isoquinoline	0.83	0.73	0.67	0.16
Linalool	1.33	1.52	1.58	-0.25
Linalyl acetate	1.09	1.35	1.44	-0.35
Lindane	-0.78	-0.7	-0.61	-0.17
L-Menthone	1.58	1.24	1.22	0.36
Malathion	-2.86	-3.04	-3.11	0.25
Malonic acid	-2.14	-2.07	-2.09	-0.05
m-Cresol	1.28	1.49	1.5	-0.22
Menthol	0.86	1.26	1.28	-0.43
Methacrolein	4.32	4.27	4.31	0.01
Methanethiol	5.08	5.02	5.04	0.04
Methanol	4.23	4.2	4.19	0.04
Methoxybenzene	2.7	3.05	3.04	-0.35
Methyl acetate	4.45	4.32	4.33	0.12
Methyl acrylate	4.07	4.02	4.02	0.04
Methyl arachidate	-3.82	-4.14	-4.14	0.32
Methyl benzoate	1.7	1.68	1.67	0.03

Methyl cellosolve	3.11	2.94	2.95	0.16
Methyl decanoate	0.75	0.56	0.56	0.19
Methyl docosanoate	-5.05	-5.08	-5.07	0.02
Methyl formate	4.89	4.81	4.82	0.07
Methyl heneicosanoate	-4.5	-4.61	-4.6	0.1
Methyl heptacosanoate	-7.38	-7.43	-7.41	0.03
Methyl heptadecanoate	-2.52	-2.73	-2.7	0.18
Methyl hexacosanoate	-6.9	-6.96	-6.96	0.06
Methyl hexadecanoate	-2.05	-2.26	-2.26	0.21
Methyl hexanoate	2.69	2.44	2.45	0.24
Methyl hydroxyacetate	2.48	2.35	2.35	0.13
Methyl isoamyl ketone	2.85	3.01	3.01	-0.16
Methyl isobutyl ketone	3.43	3.48	3.48	-0.05
Methyl laurate	-0.15	-0.38	-0.35	0.2
Methyl levulinate	1.65	2.13	2.12	-0.48
Methyl methacrylate	3.71	3.75	3.74	-0.03
Methyl nonadecanoate	-3.36	-3.67	-3.65	0.29
Methyl nonanoate	1.23	1.03	1.03	0.2
Methyl octacosanoate	-7.86	-7.9	-7.89	0.03
Methyl octadecanoate	-3.07	-3.2	-3.19	0.12
Methyl octanoate	1.7	1.5	1.53	0.17
Methyl pentacosanoate	-6.43	-6.49	-6.48	0.05
Methyl pentadecanoate	-1.57	-1.79	-1.79	0.22
Methyl propanoate	4.04	3.85	3.86	0.18
Methyl propyl sulfide	3.83	3.78	3.78	0.05
Methyl t-butyl sulfide	3.82	3.84	3.89	-0.07
Methyl tetracosanoate	-5.94	-6.02	-6	0.06
Methyl tetradecanoate	-1.18	-1.32	-1.32	0.14
Methyl t-pentyl ether	4	4	4	0
Methyl tridecanoate	-0.64	-0.85	-0.83	0.19
Methyl undecanoate	0.29	0.09	0.11	0.18
Methyl valerate	3.15	2.91	2.91	0.24
Methylacetamide	1.2	1.64	1.7	-0.5
Methylacetoacetate	2.39	2.6	2.59	-0.2
Methylamine	5.36	5.08	5.08	0.28
Methylbenzene	3.55	3.24	3.22	0.33
Methylchloroacetate	3	3.25	3.24	-0.24
Methylcyclohexane	3.79	3.59	3.59	0.2
Methylcyclopentane	4.26	3.72	3.69	0.57
Methylenanthate	2.23	1.97	1.98	0.25
Methylenecyclohexane	3.73	3.02	2.98	0.75
Methylisocyanate	4.78	4.8		-0.02

Methyllactate	2.66	2.4	2.4	0.26
Methylmalonic acid	-2.96	-2.28	-2.24	-0.72
Methylnitrite	5.63	5.34	5.23	0.4
Methyl-n-propylketone	3.69	3.69	3.69	0
Methyloxiran	4.87	4.72	4.7	0.17
Methylparathion	-2.33	-2.16	-2.18	-0.15
Methylphosphonic acid dimethyl ester	2.05	2.34		-0.29
Methylpropylether	4.79	4.44	4.44	0.35
Methylthio bis(trifluoromethyl)phosphine	3.89	3.86	3.81	0.08
Metolachlor	-2.38	-2.36	-2.27	-0.11
Molinate	-0.13	-0.17		0.04
Monocrotophos	-2.03	-2.04	-2.04	0.01
Morpholine	3.12	3.58	3.53	-0.41
MTBE	4.54	4.47	4.47	0.07
m-Toluidine	1.6	1.29	1.25	0.35
Mustard gas	1.18	1.62	1.66	-0.48
Myristyl alcohol	-1.82	-1.72	-1.7	-0.12
N,N-Dibutylformamide	0.61	0.34	0.27	0.34
N,N-Diethylaniline	1.26	1.37	1.39	-0.13
N,N-Diethylformamide	2.17	2.22	2.16	0.01
N,N-Dimethyl-1-naphthylamine	0.02	-0.31	-0.45	0.47
N,N-Dimethyl-2,3-dimethyl-3-phenyl-2-butanamine	0.26	0.64	0.68	-0.42
N,N-Dimethyl-3-methyl-3-phenyl-2-butaneamine	0.81	0.79	0.82	-0.01
N,N-Dimethylaniline	1.99	2.19	2.23	-0.24
N,N-Dimethylbenzylamine	2.25	1.95	1.93	0.32
N,N-Dimethyldodecylamine	-0.07	-0.11	-0.09	0.02
N,N-Dimethylethanolamine	2.93	2.64	2.64	0.29
N,N-Dimethylformamide	2.74	3.04	3.19	-0.45
N,N-Dimethylhexadecylamine	-1.99	-1.99	-1.96	-0.03
N,N-Dimethyloctylamine	1.81	1.77	1.77	0.04
N,N-Dimethyltetradecylamine	-1.04	-1.05	-1.05	0.01
N,N-Dipropylformamide	1.5	1.28	1.22	0.28
Naphthalene	1.03	1	0.98	0.05
N-Benzylformamide	-1.35	-1.31	-1.32	-0.03
n-Butyl acetate	3.18	3.1	3.1	0.08
n-Butyl acrylate	2.86	2.8	2.81	0.05
n-Butyl methyl ether	4.26	3.97	3.94	0.32
n-Butylbenzoate	0.67	0.46	0.44	0.23
n-Butylethylether	3.86	3.69	3.69	0.17
N-Butylformamide	0.51	0.39	0.35	0.16
n-Butylformate	3.58	3.59	3.6	-0.02
n-Butylmethacrylate	2.47	2.53	2.52	-0.05

n-Butylmethylsulfide	3.32	3.31	3.32	0
n-Decyl alcohol	0.06	0.16	0.17	-0.11
n-Decylacetate	0.49	0.28	0.31	0.18
n-Decylamine	1.05	0.91	0.92	0.13
n-Dodecylamine	0.31	-0.03	-0.02	0.33
Neburon	-5.2	-5.13		-0.07
Neohexane	4.63	4.41	4.41	0.22
N-Ethylmorpholine	2.84	3.26	3.25	-0.41
n-Heptaldehyde	2.67	2.49	2.5	0.17
n-Heptylamine	2.46	2.32	2.33	0.13
n-Hexanoic acid	0.78	1.14	1.14	-0.36
n-Hexylacetate	2.24	2.16	2.16	0.08
n-Hexylamine	2.85	2.79	2.8	0.04
Nitrobenzene	1.51	1.46	1.46	0.05
Nitrocyclohexane	1.67	1.76		-0.09
Nitroethane	3.44	3.64	3.71	-0.27
Nitroisobutane	2.94	2.96	2.98	-0.04
N-Methyl-2,3-dimethyl-3-phenyl-2-butaneamine	0.33	0.55	0.57	-0.24
N-Methyl-2-pyrrolidone	1.66	1.67	1.75	-0.09
N-Methyl-3-methyl-3-phenyl-2-butaneamine	1.01	0.7	0.69	0.32
N-Methylformamide	1.55	1.74	1.82	-0.27
N-Methylmorpholine	3.48	3.67	3.67	-0.19
N-Methylpiperidine	3.41	3.51	3.49	-0.08
N-Methylpropionamide	1.57	1.17	1.11	0.46
N-Methylpyrrolidine	4.13	4.24	4.24	-0.11
N-Methylsuccinimide	0.32	0.33	0.39	-0.07
N-Nitrosodiethylamine	2.2	1.85	1.74	0.46
N-Nitrosodimethylamine	2.7	2.67	2.67	0.03
N-Nitrosopiperidine	1.27	1.18	1.13	0.14
N-Nitrosopyrrolidine	1.07	1.55	1.68	-0.61
n-Nonanal	1.93	1.55	1.53	0.4
n-Nonylamine	1.59	1.38	1.39	0.2
n-Nonylmercaptan	1.48	1.24	1.23	0.25
n-Octanoic acid	-0.34	0.2	0.21	-0.55
n-Octylacetate	1.39	1.22	1.22	0.17
n-Octylamine	1.89	1.85	1.85	0.04
Nonacosane	-6.99	-6.81	-6.82	-0.17
Nonadecane	-2.18	-2.11	-2.11	-0.07
Nonafluorocyclopentane	4.78	4.8	4.81	-0.03
Nonane	2.76	2.59	2.6	0.16
Nonanedinitrile	0.71	0.19	0.14	0.57
Nonanedioic acid	-5.07	-4.89	-4.9	-0.17

Nonylbenzene	-0.25	-0.52	-0.5	0.25
Nopinene	2.61	2.64	2.61	0
n-Propyl acetate	3.65	3.57	3.57	0.08
n-Propyl benzoate	1.06	0.93	0.92	0.14
n-Propyl iodide	3.76	3.6	3.59	0.17
n-Propylformate	4.05	4.06	4.08	-0.03
n-Propylnitrate	3.5	3.54	3.55	-0.05
n-Undecylamine	0.46	0.44	0.44	0.02
n-Valeraldehyde	3.67	3.43	3.46	0.21
O,O-Diethyl O-2-diethylaminoethyl phosphate	-0.59	-0.68	-0.67	0.08
O,O-Diethyl O-2-diethylaminoethyl thiophosphate	-0.56	-0.68	-0.73	0.17
O,O-Diethyl O-2-dimethylaminoethyl thiophosphate	-0.01	0.14	0.18	-0.18
O,O-Diethyl O-3-(1-dimethylamino)prop-2-yl thiophosphate	-0.2	-0.09	-0.12	-0.08
O,O-Diethyl O-3-diethylaminopropyl thiophosphate	-1	-1.15	-1.18	0.18
O,O-Diethyl S-2-diethylaminoethyl dithiophosphate	-1.35	-1.4	-1.46	0.11
o-Cresol	1.59	1.49	1.47	0.12
Octachlorodibenzofuran	-6.59	-6.58	-6.56	-0.02
Octachlorodibenzo-p-dioxin	-7.18	-7.06	-6.99	-0.19
Octachloronaphthalene	-4.25	-4.12	-4.12	-0.13
Octacosane	-6.5	-6.34	-6.35	-0.15
Octadecane	-1.7	-1.64	-1.64	-0.06
Octamethylcyclotetrasiloxane	2.08	2.18	2.19	-0.11
Octamethyltrisiloxane	2.72	2.73		-0.01
Octane	3.26	3.06	3.07	0.19
Octanedioic acid	-4.74	-4.42	-4.42	-0.32
Octatetracontane	-15.97	-15.74	-15.72	-0.25
Octatriacontane	-11.29	-11.04	-11.05	-0.24
Octyl glycolate	-0.22	-0.74	-0.75	0.53
Octyl-1,2-dinitrate	-0.04	-0.1	-0.1	0.06
Octylbenzene	0.24	-0.05	-0.05	0.29
o-Cumenol	1.21	0.81	0.8	0.41
o-Toluidine	1.53	1.29	1.25	0.28
Oxetane	4.65	4.9	4.88	-0.23
Oxirane	5.24	4.95	4.91	0.33
Parathion	-2.87	-2.72	-2.77	-0.1
PCNB	-1.83	-1.74	-1.73	-0.1
p-Cresol	1.24	1.49	1.51	-0.27
p-Cymene	2.33	2.3	2.31	0.02
Pelargonic acid	-0.79	-0.27	-0.23	-0.56
Pentachlorobenzene	0.24	0.3	0.28	-0.05
Pentachloroethane	2.67	2.78	2.78	-0.11

Pentachlorophenol	-1.83	-1.45	-1.47	-0.36
Pentacontane	-16.84	-16.68	-16.65	-0.19
Pentacosane	-5.09	-4.93	-4.9	-0.19
Pentadecane	-0.24	-0.23	-0.2	-0.04
Pentadecylamine	-1.44	-1.44	-1.44	0
Pentaethylbenzene	-0.34	-0.15	-0.06	-0.28
Pentafluoro(fluorosulfonato)sulfur	4.77	4.75		0.02
Pentafluorobenzene	3.96	4	4.03	-0.08
Pentafluoroethane	6.12	5.75	5.75	0.37
Pentafluorophenol	2.48	2.25	2.25	0.23
Pentane	4.8	4.47	4.49	0.31
Pentane-1,4-dinitrate	0.97	0.97	0.97	0
Pentanedinitrile	2.23	2.07	2.02	0.21
Pentanedioic acid	-2.91	-3.01	-3.02	0.11
Pentanenitrile	2.99	3.27	3.3	-0.31
Pentanoic acid	1.53	1.61	1.58	-0.05
Pentatriacontane	-9.9	-9.63	-9.64	-0.25
Pentyl butyrate	2.04	1.69	1.69	0.35
Pentyl glycolate	1.16	0.67	0.66	0.5
Pentyl t-octyl ether	1.31	1.31	1.33	-0.03
Pentyl-1,2-dinitrate	1.38	1.31	1.3	0.08
Pentylcyclopentane	2.18	2.44	2.41	-0.23
Perchlorobiphenyl	-5.86	-5.8	-5.75	-0.11
Perfluoro-1,4-dithiane	4.24	4.18	4.1	0.14
Perfluoro-1,4-dithiane S,S'-bis(tetrafluoride)	3.1	3.46		-0.36
Perfluoro-2-(fluorosulfato)butane	4.24	4.25		-0.01
Perfluoro-2,3-dimethylbutane	4.43	4.62	4.71	-0.28
Perfluoro-2-methylbutane	4.94	4.97	4.96	-0.02
Perfluoro-2-methylpentane	4.48	4.6	4.59	-0.11
Perfluoro-3-methylpentane	4.48	4.6	4.57	-0.09
Perfluorobutane	5.44	5.32	5.27	0.17
Perfluorobutylhexane	2.64	2.51	2.53	0.11
Perfluorobutyloctane	1.76	1.57	1.62	0.14
Perfluorobutylpentane	3.07	2.98	2.99	0.08
Perfluorocyclobutane	5.5	5.14	5.08	0.42
Perfluorocyclohexane	4.47	4.4	4.38	0.09
Perfluorocyclopentane	5.05	5.03	5.03	0.02
Perfluoro-dibutylmethylamine	2.92	2.97		-0.05
Perfluorodiethylsulfur disulfide	4.27	4.17	4.21	0.06
Perfluorodiglyme	4.28	4.35	4.36	-0.08
Perfluorodimethylsulfur difluoride	5.07	5.09	5.13	-0.06
Perfluoroethane	6.5	6.06	6.03	0.47

Perfluoroethylcyclohexane	3.7	3.68	3.67	0.03
Perfluoroethylmethylsulfide	5.29	5.3	5.31	-0.02
Perfluoroethylmethylsulfur difluoride	4.7	4.63	4.61	0.09
Perfluorohexane	4.47	4.58	4.56	-0.09
Perfluorohexanoic acid	1.84	1.63	1.64	0.2
Perfluorohexylhexane	1.86	1.77	1.79	0.07
Perfluoromethylcyclohexane	4.15	4.05	4.08	0.07
Perfluoromethylpropylsulfide	4.8	4.93	4.95	-0.15
Perfluoromethylpropylsulfur difluoride	4.26	4.26	4.27	-0.01
Perfluorooctylethylene	2.68	3.08	3.15	-0.47
Perfluoropentane	4.94	4.95	4.95	-0.01
Perfluoropropane	5.95	5.69	5.64	0.31
Perfluoropropene	5.87	6.09		-0.22
Perfluorotetrahydrofuran	5.41	5.01	4.93	0.48
Perfluorotetrahydrothiophene	4.79	4.8	4.78	0.01
Perfluorotoluene	3.59	3.83	3.87	-0.28
Perfluoro-trans-1,2-dimethylcyclobutane	4.7	4.44	4.35	0.34
Perfluoro-trans-decalin	2.96	2.94	2.96	0
Perfluorotributylamine	1.83	1.94	2	-0.17
Perfluorotriethylamine	4.24	4.16	4.14	0.1
Perfluorotriglyme	3.5	3.34	3.19	0.31
Perfluorotripropylamine	3.05	3.05		0
Permethrin	-5.51	-5.39	-5.58	0.07
Perylene	-6.26	-6.14	-6.23	-0.03
Phenanthrene	-1.59	-1.5	-1.55	-0.04
Phencyclidine	-1.55	-1.46	-1.48	-0.07
Phenethyl acetate	1.12	0.93	0.93	0.19
Phenethyl alcohol	1.08	0.81	0.81	0.27
Phenethyl butyrate	0.28	-0.01	-0.01	0.29
Phenethyl hexanoate	-0.64	-0.95	-0.96	0.32
Phenethyl propionate	0.68	0.46	0.44	0.24
Phenol	1.67	1.75	1.73	-0.06
Phenthoate	-3.46	-3.37	-3.37	-0.09
Phenylacetic acid	-0.29	-0.09	-0.16	-0.13
Phenylisocyanate	2.55	2.56		-0.01
Phlorol	1.33	1.02	1.01	0.32
Phorate	-0.6	-0.79		0.19
Phosmet	-4.7	-4.69		-0.01
Phytane	-1.74	-1.54	-1.51	-0.23
Phytol	-3.59	-3.36	-3.41	-0.17
Pimelonitrile	1.29	1.13	1.04	0.25
Pinacolone	3.64	3.63	3.63	0.01

Piperidine	3.61	3.42	3.38	0.23
p-Menthadiene	2.44	1.93	1.87	0.57
Pristane	-1.25	-1.07	-1.07	-0.18
Prometryn	-3.77	-3.7		-0.07
Propanal	4.63	4.37	4.36	0.27
Propane	5.97	5.41	5.42	0.55
Propane-1,3-dithiol	2.71	2.71	2.68	0.03
Propanenitrile	3.8	4.21	4.26	-0.46
Propanil	-2.27	-2.44	-2.34	0.07
Propanoic acid	2.67	2.55	2.53	0.14
Propanolamine	2.31	2.24	2.23	0.08
Propanone	4.51	4.63	4.63	-0.12
Propazine	-4.76	-4.48		-0.28
Propenal	4.58	4.54	4.56	0.02
Propoxur	-2.77	-2.79	-2.79	0.02
Propyl 2-methylbutanoate	2.66	2.42	2.43	0.23
Propyl butyrate	2.8	2.63	2.63	0.17
Propyl glycolate	1.97	1.61	1.62	0.35
Propyl lactate	2.08	1.66	1.65	0.43
Propyl nitrite	4.63	4.59	4.5	0.13
Propyl propanoate	3.26	3.1	3.12	0.14
Propyl tert-amyl ether	3.3	3.25	3.24	0.06
Propyl t-octyl ether	2.34	2.25	2.23	0.11
Propyl-1,2-dinitrate	2.18	2.25	2.26	-0.08
Propylacetamide	0.58	0.76	0.72	-0.14
Propylcyclohexane	2.74	2.65	2.63	0.11
Propylcyclopentane	3.21	2.9	2.89	0.32
Propylene	5.77	5.58	5.59	0.18
Propylenediamine	3.17	3.3	3.3	-0.13
Propylsilane	5.06	4.97		0.09
Propyne	5.51	5.61	5.63	-0.12
p-Toluidine	1.54	1.29	1.31	0.23
Pulegone	1.24	0.98	0.96	0.28
Pyrene	-3.38	-3.64	-3.69	0.31
Pyridine	3.44	3.23	3.19	0.25
Pyrrolidine	3.93	4.15	4.09	-0.16
Pyruvaldehyde	3.55	3.59	3.55	0
Quinoline	0.92	0.57		0.35
R-1234yf	5.83	5.79		0.04
R134a	5.82	5.78	5.83	-0.01
Raspberry ketone	-0.45	-0.7	-0.7	0.25
Refrigerant 115	5.96	5.68	5.65	0.31

s-Butylacetate	3.35	3.34	3.38	-0.03
s-Butylamine	4.37	4.04	4.04	0.33
s-Butylbenzene	2.35	2.09	2.09	0.26
s-Butylnitrite	4.27	4.36	4.37	-0.1
Sebaconitrile	0.37	-0.28	-0.37	0.74
sec-Hexyl acetate	2.72	2.66	2.65	0.07
s-Phenethyl alcohol	1.38	1.33	1.29	0.08
s-trans-1,3-Butadiene	5.45	5.28	5.29	0.16
Styrene	2.95	2.94	2.95	0
Styrene oxide	1.6	2.08	2.06	-0.46
Suberonitrile	1.03	0.66	0.57	0.46
Sulcatone	2.22	2.68	2.7	-0.49
t-Butyl lactate	2.24	1.69	1.62	0.62
t-Butylacetamide	0.78	0.92	0.96	-0.18
t-Butylamine	4.69	4.36	4.31	0.38
t-Butylbenzene	2.46	2.24	2.25	0.21
t-Butylhydroperoxide	2.86	2.92		-0.06
t-Butylmercaptan	4.38	4.12	4.04	0.34
t-Butylnitrite	4.39	4.62	4.68	-0.29
t-Butylthio bis(trifluoromethyl)phosphine	2.95	2.96	3.01	-0.06
Terbufos	-1.46	-1.2		-0.26
Terbutryn	-3.66	-3.69		0.03
Tetrachloroethylene	3.4	3.64	3.79	-0.39
Tetracontane	-12.26	-11.98	-11.95	-0.31
Tetracosane	-4.63	-4.46	-4.43	-0.2
Tetradecane	0.11	0.24	0.25	-0.14
Tetradecylbenzene	-2.7	-2.87	-2.86	0.16
Tetraethylmethane	3.01	3	3.05	-0.04
Tetrafluoroethene	6.53	6.62	6.76	-0.23
Tetrahydrofuran	4.33	4.09	4.1	0.23
Tetrahydropyran	3.98	3.96	3.91	0.07
Tetramethylene glycol	0.16	0.28	0.26	-0.1
Tetramethylethene	4.22	4.6	4.65	-0.43
Tetrapentacontane	-18.53	-18.56	-18.53	0
Tetratetracontane	-14.12	-13.86	-13.87	-0.25
Thianaphthene	1.45	1.52	1.46	-0.01
Thietane	3.85	3.86	3.84	0.01
Thiobencarb	-2.75	-2.65		-0.1
Thiolane	3.41	3.7	3.68	-0.27
Thiophene	4.02	3.9	3.83	0.19
TNT	-2.76	-2.88	-2.95	0.19
Tolclofos-methyl	-1.24	-1.66	-1.69	0.45

trans,trans-2,4-Hexadienyl acetate	1.95	2.56	2.56	-0.61
trans-1,2-Dibromocycloheptane	1.33	1.41	1.4	-0.07
trans-1,2-Dibromocyclohexane	1.49	1.54	1.55	-0.06
trans-1,2-Dibromocyclooctane	1.04	1.28	1.31	-0.27
trans-1,2-Dibromocyclopentane	2.15	2.27	2.29	-0.14
trans-1,2-Dibromoethene	3.61	3.58		0.03
trans-1,2-Dichloroethylene	4.42	4.44	4.47	-0.05
trans-1,2-Dimethylcyclohexane	3.29	3.38	3.33	-0.04
trans-1,2-Dimethylcyclopentane	3.93	4.11	4.11	-0.18
trans-1,3,5-Hexatriene	4.08	4.54	4.54	-0.46
trans-1,3-Dichloropropene	3.67	3.72	3.73	-0.06
trans-1,3-Dimethylcyclohexane	3.37	3.38	3.36	0.01
trans-1,3-Dimethylcyclopentane	3.93	4.11	4.1	-0.17
trans-1,3-Pentadiene	4.76	4.84	4.84	-0.08
trans-1,4-Dichloro-2-butene	2.66	3	2.99	-0.33
trans-1,4-Dimethylcyclohexane	3.47	3.38	3.37	0.1
trans-1,4-Hexadiene	4.37	4.37	4.37	0
trans-1-Propenylbenzene	2.27	2.5	2.52	-0.25
trans-2-Butene	5.37	5.14	5.13	0.24
trans-2-Decene	2.27	2.32	2.31	-0.04
trans-2-Dodecene	1.21	1.38	1.39	-0.18
trans-2-Heptene	3.85	3.73	3.74	0.11
trans-2-Hexene	4.32	4.2	4.2	0.12
trans-2-Methoxy-4-(1-propenyI)phenol	-0.22	0.3	0.31	-0.53
trans-2-Methylcyclohexanol	2.22	2.15	2.15	0.07
trans-2-Octene	3.38	3.26	3.26	0.11
trans-2-Pentene	4.84	4.67	4.69	0.15
trans-2-Phenylcyclopropylamine	1.19	1.04	0.97	0.22
trans-3-Heptene	3.83	3.73	3.74	0.09
trans-3-Hexene	4.32	4.2	4.21	0.11
trans-3-Methylcyclohexanol	1.9	2.15	2.11	-0.21
trans-3-Octene	3.36	3.26	3.25	0.11
trans-4,4-Dimethyl-2-pentene	4.17	4.14	4.17	0
trans-4,6-Dimethyl-1,3-dioxane	2.73	2.99	3.07	-0.35
trans-4-Octene	3.39	3.26	3.26	0.13
trans-Crotononitrile	3.36	3.94	3.97	-0.61
trans-Cycloheptane-1,2-dinitrate	0.05	0.41	0.42	-0.37
trans-Cyclohexane-1,2-dinitrate	0.75	0.54	0.46	0.29
trans-Cyclohexane-1,3-dinitrate	0.71	0.54	0.5	0.21
trans-Decalin	2.22	2.4	2.36	-0.14
Triacontane	-7.48	-7.28	-7.29	-0.19
Triallate	-1.8	-1.99	-2.05	0.25

Tributyl borate	1.04	0.79	0.75	0.29
Tributyl phosphate	-0.82	-1.39	-1.51	0.69
Tributylamine	1.32	0.95	0.96	0.36
Trichloroacetic acid	1.34	1.45	1.42	-0.08
Trichloroacetylchloride	3.44	3.18	3.07	0.37
Trichloroethylene	3.98	4.04	4.06	-0.08
Trichlorosyringol	-1.11	-1.07	-1.02	-0.09
Trichlorotoluene	1.54	1.67	1.68	-0.14
Triclopyr	-2.54	-2.23	-2.07	-0.47
Tricosane	-4.4	-3.99	-3.99	-0.41
Tridecane	0.76	0.71	0.73	0.03
Tridecylamine	-0.5	-0.5	-0.49	-0.01
Tridecylbenzene	-2.75	-2.4	-2.38	-0.37
Triethanolamine	-3.32	-3.59	-3.66	0.34
Triethyl borate	3.35	3.61	3.67	-0.32
Triethyl phosphate	1.25	1.43	1.47	-0.22
Triethylamine	3.88	3.77	3.79	0.09
Triisobutyl borate	1.68	1.57	1.55	0.13
Triisopropyl borate	3.06	2.92	2.85	0.21
Trimethoxyborine	4.26	4.45	4.51	-0.25
Trimethyl phosphate	2.07	2.27	2.34	-0.27
Trimethylamine	5.33	5	5.02	0.31
Trimethylethylene	4.8	4.87	4.9	-0.1
Tripropyl borate	2.21	2.2	2.2	0.01
Tripropyl phosphate	0.04	0.02	0.06	-0.02
Tripropylamine	2.3	2.36	2.4	-0.1
Triptane	4.14	4.2	4.21	-0.07
Undecane	1.73	1.65	1.64	0.09
Undecanedioic acid	-5.28	-5.83	-5.88	0.6
Undecylbenzene	-1.34	-1.46	-1.46	0.12
Vinclozolin	-3.89	-3.94		0.05
Vinyl acetate	4.19	4.39	4.48	-0.29
Vinylidenechloride	4.88	4.83	4.92	-0.04
Vinylpropionate	3.76	3.92	3.98	-0.22
Z-1-Chloro-2,3,3,3-tetrafluoropropene	5.17	5		0.17
Zingiberene	-0.03	-0.61	-0.65	0.62
