# Supplementary material for: Calculation of the Vapour Pressure of Organic Molecules by Means of a Group-Additivity Method and Their Resultant Gibbs Free Energy and Entropy of Vaporization at 298.15 K
Source: Molecules. 2021 Feb 17;26(4):1045. doi: 10.3390/molecules26041045 (PMC7922249; doi:10.3390/molecules26041045)
Supplement: Supplementary file 1 [file molecules-26-01045-s001.zip › molecules-1089923-SM-proofed/S03. Experimental vs. calculated deltaG_vap) Data Table.pdf]

**Molecule name**	**deltaG °(vap) exp**	**deltaG °(vap) calc**	**Deviation**	**Dev in %**
(-)-Methyl jasmonate	33.76	35.31	-1.55	-4.59
(-)-trans-Pinocarveol	20.94	21.72	-0.78	-3.72
(+)-trans-Myrtanol	26.49	22.81	3.68	13.89
(2-Bromoethyl)benzene	19.95	20.41	-0.46	-2.31
(Chloromethyl)benzene	15.79	16.19	-0.40	-2.53
(E)-1,3,3,3-Tetrafluoropropene	-3.95	-3.91	-0.04	1.01
(E)-1-Chloro-3,3,3-trifluoropropene	-0.62	0.72	-1.34	216.13
(Z)-1,3,3,3-Tetrafluoropropene	-1.57	-3.91	2.34	-149.04
1-(1,1-Difluoroethoxy)-1,1,2,2-tetrafluoroethane	5.67	3.97	1.70	29.98
1-(2,2-Difluoroethoxy)-1,1,2,2,2-pentafluoroethane	2	2.2	-0.20	-10.00
1-(2,2-Difluoroethoxy)-1,1,2,2,3,3,3-heptafluoropropane	4.22	4.31	-0.09	-2.13
1-(2-Aminoethyl)-4-methylpiperazine	20.46	19.44	1.02	4.99
1,1,1,2,2,3,3,4,4,5,5-Undecafluoro-5-methoxypentane	5.91	5.68	0.23	3.89
1,1,1,2,2,3,3,4,4-Nonafluoro-4-propoxybutane	6.98	7.85	-0.87	-12.46
1,1,1,2,2,3,3-Heptafluoro-3-(2,2,2-trifluoroethoxy)propane	2.61	2.54	0.07	2.68
1,1,1,2,2,3,3-Heptafluoro-3-(2,2,3,3,3-pentafluoropropoxy)propane	4.52	4.66	-0.14	-3.10
1,1,1,2,2,3,3-Heptafluoro-3-(2,2,3,3-tetrafluoropropoxy)propane	5.92	6.43	-0.51	-8.61
1,1,1,2,2,3,3-Heptafluoro-3-(fluoromethoxy)propane	1.77	3	-1.23	-69.49
1,1,1,2,2,3,3-Heptafluoro-4-methoxybutane	4.55	4.26	0.29	6.37
1,1,1,2,2-Pentafluoro-3-(1,1,2,2-tetrafluoroethoxy)propane	4.6	4.31	0.29	6.30
1,1,1,2,2-Pentafluoro-3-methoxypropane	2.26	2.14	0.12	5.31
1,1,1,2,2-Pentafluoro-3-pentafluoroethoxypropane	2.11	2.54	-0.43	-20.38
1,1,1,2,2-Pentafluoropropane	-3.77	-3.39	-0.38	10.08
1,1,1,2,3,3,3-Heptafluoropropane	-3.71	-2.59	-1.12	30.19
1,1,1,2,3,3,4,4-Octafluoro-4-methoxy-2-trifluoromethylbutane	5.81	5.57	0.24	4.13
1,1,1,2,3,3-Hexafluoro-3-(2,2,2-trifluoroethoxy)propane	4.87	3.86	1.01	20.74
1,1,1,2,3,3-Hexafluoro-3-(2,2,3,3,3-pentafluoropropoxy)propane	6.46	5.97	0.49	7.59
1,1,1,2,3,3-Hexafluoro-3-(2,2,3,3-tetrafluoropropoxy)propane	8.61	7.74	0.87	10.10
1,1,1,2,3,3-Hexafluoro-3-methoxypropane	2.87	2.77	0.10	3.48
1,1,1,2,3,3-Hexafluoro-4-methoxybutane	6.25	6.88	-0.63	-10.08

1,1,1,2,3,3-Hexafluoro-4-trifluoromethoxybutane	3.89	2.2	1.69	43.44
1,1,1,2,3,3-Hexafluoropropane	-2.34	-0.82	-1.52	64.96
1,1,1,2,4,4,4-Heptafluoro-2-trifluoromethoxybutane	2.48	4.66	-2.18	-87.90
1,1,1,2,4,4-Hexafluoro-2-(trifluoromethoxy)butane	3.34	2.89	0.45	13.47
1,1,1,2-Tetrachlorodifluoroethane	6.54	5.63	0.91	13.91
1,1,1,2-Tetrachloroethane	10.22	10.08	0.14	1.37
1,1,1,2-Tetrafluoro-2-(trifluoromethoxy)butane	1.96	1.63	0.33	16.84
1,1,1,3,3,3-Hexafluoro-2-fluoromethoxypropane	3.39	2.37	1.02	30.09
1,1,1,3,3,3-Hexafluoro-2-methoxy-2-(trifluoromethyl)propane	2.71	1.52	1.19	43.91
1,1,1,3,3,3-Hexafluoro-2-methoxypropane	2.56	0.83	1.73	67.58
1,1,1,3,3,3-Hexafluoropropane	-2.4	-3.34	0.94	-39.17
1,1,1,3,3-Pentafluoro-3-methoxy-2-trifluoromethylpropane	4.42	2.72	1.70	38.46
1,1,1,3,3-Pentafluorobutane	1.44	-0.71	2.15	149.31
1,1,1,3,3-Pentafluoropropane	-2.68	-1.57	-1.11	41.42
1,1,1-Trichloroethane	4.54	3.97	0.57	12.56
1,1,1-Trichloropropane	7.91	6.65	1.26	15.93
1,1,1-Trichlorotrifluoroethane	1.85	3.46	-1.61	-87.03
1,1,1-Trifluoro-2-(1,1,2-trifluoroethoxy)ethane	4.07	2.03	2.04	50.12
1,1,1-Trifluoro-2-(2,2,2-trifluoroethoxy)ethane	3.93	1.12	2.81	71.50
1,1,1-Trifluoroethane	-6.25	-5.5	-0.75	12.00
1,1,2,2,3,3,4,4-Octafluoro-5-methoxypentane	10.48	8.14	2.34	22.33
1,1,2,2,3,3-Hexafluoro-1-methoxypropane	4.25	3.23	1.02	24.00
1,1,2,2,3-Pentafluoropropane	0.11	-0.54	0.65	590.91
1,1,2,2-Tetrachloro-1,2-difluoroethane	6.79	5.97	0.82	12.08
1,1,2,2-Tetrachloroethane	12.38	12.48	-0.10	-0.81
1,1,2,2-Tetrafluoro-1-(2,2,2-trifluoroethoxy)ethane	3.15	2.2	0.95	30.16
1,1,2,2-Tetrafluoro-1-fluoromethoxyethane	2.89	2.49	0.40	13.84
1,1,2,2-Tetrafluoro-3-(1,1,2,2-tetrafluoroethoxy)propane	7.22	6.08	1.14	15.79
1,1,2,2-Tetrafluoro-3-methoxypropane	4.9	3.91	0.99	20.20
1,1,2,2-Tetrafluoro-3-pentafluoroethoxypropane	3.76	4.31	-0.55	-14.63
1,1,2,2-Tetrafluoro-3-trifluoromethoxypropane	2.05	0.55	1.50	73.17
1,1,2-Trichloroethane	8.62	9.85	-1.23	-14.27
1,1,2-Trichloropropane	13.67	10.71	2.96	21.65
1,1,2-Trifluoro-1-methoxyethane	1.8	0.95	0.85	47.22
1,1,2-Trifluoroethane	-1.83	-2.65	0.82	-44.81
1,1,3-Trimethylcyclopentane	7.3	6.94	0.36	4.93
1,10-Decanediol	41.68	43.13	-1.45	-3.48
1,10-Decanediol dinitrate	39.99	37.82	2.17	5.43
1,11-Undecanediol	46.13	45.75	0.38	0.82

1,12-Dodecanediol	48.94	48.44	0.50	1.02
1,13-Tridecanediol	51.37	51.12	0.25	0.49
1,14-Tetradecanediol	53.97	53.8	0.17	0.31
1,15-Pentadecanediol	56.67	56.48	0.19	0.34
1,16-Hexadecanediol	58.55	59.17	-0.62	-1.06
1,1-Bis(4-methylphenyl)ethane	33.5	32	1.50	4.48
1,1-Dichloro-1-fluoroethane	0.63	0.49	0.14	22.22
1,1-Dichlorobutane	8.71	9.11	-0.40	-4.59
1,1-Dichloroethane	3.02	3.74	-0.72	-23.84
1,1-Dichloropropane	7.13	6.43	0.70	9.82
1,1-Dichlorotetrafluoroethane	-1.9	-0.02	-1.88	98.95
1,1-Diethylcyclohexane	15.87	15.27	0.60	3.78
1,1-Difluoro-2-methoxyethane	2.3	1.8	0.50	21.74
1,1-Difluoroethane	-4.4	-3.73	-0.67	15.23
1,1-Difluoroethylene	-8.85	-7.5	-1.35	15.25
1,1-Dimethylcyclohexane	8.73	9.91	-1.18	-13.52
1,1-Dimethylcyclopentane	5.48	5.74	-0.26	-4.74
1,1-Diphenylbutane	32.19	34.39	-2.20	-6.83
1,1-Diphenylethane	29.74	29.03	0.71	2.39
1,1-Diphenylpropane	30.08	31.71	-1.63	-5.42
1,2,3,4,5,6,7-Heptachloronaphthalene	49.4	48.44	0.96	1.94
1,2,3,4,5,6,8-Heptachloronaphthalene	49.17	48.44	0.73	1.48
1,2,3,4,5,7-Hexachloronaphthalene	44.97	44.78	0.19	0.42
1,2,3,4,6,7,8-Heptachlorodibenzofuran	61.19	62.48	-1.29	-2.11
1,2,3,4,6,7,8-Heptachlorodibenzo-p-dioxin	66.76	65.22	1.54	2.31
1,2,3,4,6,7-Hexachloronaphthalene	45.22	44.78	0.44	0.97
1,2,3,4,6,8,9-Heptachlorodibenzofuran	63.33	62.48	0.85	1.34
1,2,3,4,6,8-Hexachlorodibenzofuran	57.77	58.83	-1.06	-1.83
1,2,3,4,6-Pentachloronaphthalene	41.83	41.13	0.70	1.67
1,2,3,4,7,8,9-Heptachlorodibenzofuran	62.16	62.48	-0.32	-0.51
1,2,3,4,7,8-Hexachlorodibenzofuran	57.71	58.83	-1.12	-1.94
1,2,3,4,7,8-Hexachlorodibenzo-p-dioxin	61.9	61.56	0.34	0.55
1,2,3,4,7-Pentachlorodibenzofuran	54.91	55.17	-0.26	-0.47
1,2,3,4,7-Pentachlorodibenzo-p-dioxin	59.05	57.91	1.14	1.93
1,2,3,4-Tetrachlorobenzene	26.77	23.21	3.56	13.30
1,2,3,4-Tetrachlorodibenzofuran	51.02	51.52	-0.50	-0.98
1,2,3,4-Tetrachloronaphthalene	38.63	37.48	1.15	2.98
1,2,3,4-Tetrafluorobenzene	6.79	6.31	0.48	7.07
1,2,3,5,6,7-Hexachloronaphthalene	45.22	44.78	0.44	0.97
1,2,3,5,7,8-Hexachloronaphthalene	45.7	44.78	0.92	2.01
1,2,3,5,7-Pentachloronaphthalene	41.28	41.13	0.15	0.36
1,2,3,5,8-Pentachloronaphthalene	42.75	41.13	1.62	3.79

1,2,3,5-Tetrachlorobenzene	21.52	23.21	-1.69	-7.85
1,2,3,5-Tetrachloronaphthalene	38.54	37.48	1.06	2.75
1,2,3,5-Tetraethylbenzene	26.54	25.26	1.28	4.82
1,2,3,5-Tetrafluorobenzene	5.8	6.31	-0.51	-8.79
1,2,3,5-Tetramethylbenzene	18.26	14.53	3.73	20.43
1,2,3,6,7,8-Hexachlorodibenzofuran	57.88	58.83	-0.95	-1.64
1,2,3,6,7,8-Hexachlorodibenzo-p-dioxin	63.22	61.56	1.66	2.63
1,2,3,7,8,9-Hexachlorodibenzofuran	58.84	58.83	0.01	0.02
1,2,3,7,8,9-Hexachlorodibenzo-p-dioxin	63.22	61.56	1.66	2.63
1,2,3,7,8-Pentachlorodibenzofuran	54.17	55.17	-1.00	-1.85
1,2,3,7,8-Pentachlorodibenzo-p-dioxin	59.28	57.91	1.37	2.31
1,2,3,7-Tetrachlorodibenzofuran	50.55	51.52	-0.97	-1.92
1,2,3,7-Tetrachlorodibenzo-p-dioxin	54.66	54.26	0.40	0.73
1,2,3-Trichlorobenzene	20.35	19.55	0.80	3.93
1,2,3-Trichloronaphthalene	35.34	33.82	1.52	4.30
1,2,3-Trichloropropane	13.13	14.19	-1.06	-8.07
1,2,3-Triethylbenzene	21.4	21.1	0.30	1.40
1,2,4,5,6,8-Hexachloronaphthalene	45.81	44.78	1.03	2.25
1,2,4,5-Tetrachlorobenzene	20.78	23.21	-2.43	-11.69
1,2,4,5-Tetrafluorobenzene	6.44	6.31	0.13	2.02
1,2,4,5-Tetramethylbenzene	18.21	14.53	3.68	20.21
1,2,4,6,7,8-Hexachlorodibenzofuran	59.11	58.83	0.28	0.47
1,2,4,6,7,9-Hexachlorodibenzo-p-dioxin	62.08	61.39	0.69	1.11
1,2,4,6,8,9-Hexachlorodibenzofuran	57.31	58.83	-1.52	-2.65
1,2,4,7,8-Pentachlorodibenzofuran	55.01	55.17	-0.16	-0.29
1,2,4,7,8-Pentachlorodibenzo-p-dioxin	58.37	57.91	0.46	0.79
1,2,4,7-Tetrachloronaphthalene	37.74	37.48	0.26	0.69
1,2,4-Trichlorobenzene	19.52	19.55	-0.03	-0.15
1,2,4-Trichlorodibenzo-p-dioxin	51.4	50.61	0.79	1.54
1,2,4-Triethylbenzene	19.81	21.1	-1.29	-6.51
1,2,4-Trimethylbenzene	14.67	13.05	1.62	11.04
1,2,7,8-Tetrachlorodibenzofuran	51.29	51.23	0.06	0.12
1,2,7,8-Tetrachlorodibenzo-p-dioxin	53.46	54.26	-0.80	-1.50
1,2-Benzenedicarboxylic acid, di-2-propenyl ester	38.12	35.99	2.13	5.59
1,2-Bis(2-aminoethoxy)ethane	24.39	23.09	1.30	5.33
1,2-Bis(dimethylamino)ethane	9.57	9.74	-0.17	-1.78
1,2-Butanediamine	13.55	12.42	1.13	8.34
1,2-Butanediol	22.98	24.01	-1.03	-4.48
1,2-Dibromobenzene	20.5	19.21	1.29	6.29
1,2-Dibromobutane	13.68	14.47	-0.79	-5.77
1,2-Dibromoethane	10.48	10.31	0.17	1.62
1,2-Dibromoheptane	21	22.52	-1.52	-7.24

1,2-Dibromopropane	11.36	11.79	-0.43	-3.79
1,2-Dibromotetrafluoroethane	1.65	2.54	-0.89	-53.94
1,2-Dichloro-1,1,2-trifluoroethane	0.4	0.09	0.31	77.50
1,2-Dichlorobenzene	15.69	15.9	-0.21	-1.34
1,2-Dichlorobutane	9.47	10.76	-1.29	-13.62
1,2-Dichloronaphthalene	31.3	30.17	1.13	3.61
1,2-Dichloropropane	6.85	8.08	-1.23	-17.96
1,2-Diethylbenzene	16.36	16.93	-0.57	-3.48
1,2-Difluorobenzene	7.86	7.45	0.41	5.22
1,2-Dimethylbenzene	11.76	11.56	0.20	1.70
1,2-Dimethylnaphthalene	28.94	25.83	3.11	10.75
1,2-Diphenylethane	29.34	30.51	-1.17	-3.99
1,2-Ethanediamine	10.1	8.82	1.28	12.67
1,2-Ethanediol	22.43	21.61	0.82	3.66
1,2-Ethanediol dinitrate	15.49	16.36	-0.87	-5.62
1,2-Ethanedithiol	11.27	10.42	0.85	7.54
1,2-Hexanediol	27.85	29.37	-1.52	-5.46
1,2-Pentadiene	1.82	2.26	-0.44	-24.18
1,2-Pentanediol	25.47	26.69	-1.22	-4.79
1,2-Propanediol	21.49	21.32	0.17	0.79
1,3,5,7-Tetrachloronaphthalene	36.46	37.48	-1.02	-2.80
1,3,5,8-Tetrachloronaphthalene	38.17	37.48	0.69	1.81
1,3,5-Tribromobenzene	24.49	24.52	-0.03	-0.12
1,3,5-Trichlorobenzene	18.33	19.55	-1.22	-6.66
1,3,5-Trifluorobenzene	4.94	6.88	-1.94	-39.27
1,3,5-Trimethylbenzene	14.15	13.05	1.10	7.77
1,3,6,8-Tetrachlorodibenzofuran	48.72	51.52	-2.80	-5.75
1,3,6,8-Tetrachlorodibenzo-p-dioxin	52.38	54.26	-1.88	-3.59
1,3,7,8-Tetrachlorodibenzofuran	49.69	51.52	-1.83	-3.68
1,3,7,8-Tetrachlorodibenzo-p-dioxin	53.57	54.26	-0.69	-1.29
1,3,7,9-Tetrachlorodibenzofuran	49.98	51.52	-1.54	-3.08
1,3,7,9-Tetrachlorodibenzo-p-dioxin	52.89	54.26	-1.37	-2.59
1,3,7-Trichlorodibenzo-p-dioxin	53.94	50.61	3.33	6.17
1,3,7-Trichloronaphthalene	33.96	33.82	0.14	0.41
1,3-Butanediol dinitrate	19.53	20.35	-0.82	-4.20
1,3-Butyleneglycol	26.31	24.01	2.30	8.74
1,3-Dibromobenzene	19.73	19.21	0.52	2.64
1,3-Dibromopropane	15.7	12.99	2.71	17.26
1,3-Dichloro-2-propanol	17.07	18.13	-1.06	-6.21
1,3-Dichlorobenzene	14.95	15.9	-0.95	-6.35
1,3-Dichloronaphthalene	31.2	30.17	1.03	3.30
1,3-Dichloropropane	9.25	9.91	-0.66	-7.14

1,3-Dichloro-trans-2-butene	10.56	9.96	0.60	5.68
1,3-Diethylbenzene	16.19	16.93	-0.74	-4.57
1,3-Dihydroisobenzofuran	17.63	20.87	-3.24	-18.38
1,3-Diisopropylbenzene	18.66	19.33	-0.67	-3.59
1,3-Diisopropylnaphthalene	32.42	33.6	-1.18	-3.64
1,3-Dimethylbenzene	11.22	11.56	-0.34	-3.03
1,3-Dimethylnaphthalene	26.92	25.83	1.09	4.05
1,3-Dioxane	7.81	8.88	-1.07	-13.70
1,3-Dioxolane	5.54	6.08	-0.54	-9.75
1,3-Divinylbenzene	17.35	14.99	2.36	13.60
1,3-Pentanediamine	15.33	15.1	0.23	1.50
1,3-Propanediamine	12.84	11.51	1.33	10.36
1,3-Propanediol	24.09	24.29	-0.20	-0.83
1,3-Propanediol dinitrate	18.71	19.04	-0.33	-1.76
1,4,5-Trimethylnaphthalene	29.53	27.32	2.21	7.48
1,4-Butanediamine	15.58	14.19	1.39	8.92
1,4-Butanediol dinitrate	22.14	21.72	0.42	1.90
1,4-Butanedithiol	17.26	15.79	1.47	8.52
1,4-Dichlorobenzene	15.01	15.9	-0.89	-5.93
1,4-Dichlorobutane	12.84	12.59	0.25	1.95
1,4-Dichloronaphthalene	30.75	30.17	0.58	1.89
1,4-Diethylbenzene	16.36	16.93	-0.57	-3.48
1,4-Diisopropylbenzene	19.92	19.33	0.59	2.96
1,4-Dimethylbenzene	11.01	11.56	-0.55	-5.00
1,4-Dimethylnaphthalene	26.54	25.83	0.71	2.68
1,4-Dimethylpiperazine	10.35	10.19	0.16	1.55
1,4-Dioxane	7.41	5.06	2.35	31.71
1,4-Di-t-butylbenzene	23.42	22.98	0.44	1.88
1,4-Pentadiene	0.08	1.12	-1.04	-1300.00
1,5-Diamino-2-methylpentane	19.23	18.07	1.16	6.03
1,5-Dichloronaphthalene	31.16	30.17	0.99	3.18
1,5-Dichloropentane	16.19	15.27	0.92	5.68
1,5-Dimethylnaphthalene	26.94	25.83	1.11	4.12
1,5-Hexadiene	3.08	3.8	-0.72	-23.38
1,5-Pentanediamine	18.04	16.87	1.17	6.49
1,5-Pentanediol	30.14	29.66	0.48	1.59
1,5-Pentanediol dinitrate	24.34	24.41	-0.07	-0.29
1,5-Pentanedithiol	19.59	18.47	1.12	5.72
1,6,7-Trimethylnaphthalene	30.5	27.32	3.18	10.43
1,6-Dichloronaphthalene	31.16	30.17	0.99	3.18
1,6-Dimethylnaphthalene	26.83	25.83	1.00	3.73
1,6-Hexanediamine	19.19	19.55	-0.36	-1.88

1,6-Hexanedioic acid	47.8	48.44	-0.64	-1.34
1,6-Hexanediol dinitrate	28.29	27.09	1.20	4.24
1,6-Hexanedithiol	22.08	21.15	0.93	4.21
1,7-Dichloronaphthalene	31.16	30.17	0.99	3.18
1,7-Heptanediamine	21.48	22.24	-0.76	-3.54
1,7-Heptanediol	35.55	35.02	0.53	1.49
1,7-Heptanediol dinitrate	31.18	29.77	1.41	4.52
1,7-Heptanedithiol	23.79	23.84	-0.05	-0.21
1,8-Cineole	17.41	16.3	1.11	6.38
1,8-Dichloronaphthalene	32.59	30.17	2.42	7.43
1,8-Octanediamine	24.35	24.92	-0.57	-2.34
1,8-Octanediol	38.51	37.76	0.75	1.95
1,8-Octanediol dinitrate	34.54	32.45	2.09	6.05
1,9-Nonanediol	40.8	40.39	0.41	1.00
1-Acetyladamantane	27.32	28.86	-1.54	-5.64
1-Adamantanol	26.92	28.06	-1.14	-4.23
1-Amino-3-methoxypropane	9.84	10.14	-0.30	-3.05
1-Azidooctane	19.27	20.87	-1.60	-8.30
1-Benzylpyrazole	27.63	27.37	0.26	0.94
1-Bromo-2-chloro-1,1,2-trifluoroethane	2.54	2.2	0.34	13.39
1-Bromo-2-chloroethane	7.76	8.77	-1.01	-13.02
1-Bromo-2-ethylbenzene	19.92	18.07	1.85	9.29
1-Bromo-2-fluorobenzene	13.3	13.33	-0.03	-0.23
1-Bromo-3-fluorobenzene	12.33	13.33	-1.00	-8.11
1-Bromo-4-chlorobenzene	18.15	17.56	0.59	3.25
1-Bromo-4-fluorobenzene	12.73	13.33	-0.60	-4.71
1-Bromobutane	7.22	8.02	-0.80	-11.08
1-Bromodecane	24.49	24.12	0.37	1.51
1-Bromododecane	30.19	29.49	0.70	2.32
1-Bromonaphthalene	27.85	28.17	-0.32	-1.15
1-Bromononane	21.69	21.44	0.25	1.15
1-Bromooctane	18.83	18.75	0.08	0.42
1-Bromopentane	10.16	10.71	-0.55	-5.41
1-Bromoperfluorooctane	12.37	10.93	1.44	11.64
1-Bromopropane	5.51	5.34	0.17	3.09
1-Bromoundecane	27.34	26.8	0.54	1.98
1-Butanethiol	6.96	8.08	-1.12	-16.09
1-Butanol	11.73	11.56	0.17	1.45
1-Butene	-1.28	-0.6	-0.68	53.13
1-Butylamine	6.93	7.28	-0.35	-5.05
1-Butyne	-1.54	-0.77	-0.77	50.00
1-Chloro-1,1-difluoroethane	-3.03	-3.34	0.31	-10.23

1-Chloro-2-fluorobenzene	10.84	11.68	-0.84	-7.75
1-Chloro-3-fluorobenzene	10.04	11.68	-1.64	-16.33
1-Chloro-4-fluorobenzene	9.02	11.68	-2.66	-29.49
1-Chlorobutane	6.27	6.48	-0.21	-3.35
1-Chlorodecane	22.77	22.58	0.19	0.83
1-Chlorodibenzo-p-dioxin	39.54	43.3	-3.76	-9.51
1-Chlorododecane	29.17	27.94	1.23	4.22
1-Chloroheptane	13.87	14.53	-0.66	-4.76
1-Chlorohexane	10.9	11.85	-0.95	-8.72
1-Chloronaphthalene	25.26	26.52	-1.26	-4.99
1-Chlorononane	19.81	19.9	-0.09	-0.45
1-Chlorooctane	16.55	17.21	-0.66	-3.99
1-Chloropentadecane	33.45	35.99	-2.54	-7.59
1-Chloropentane	7.93	9.17	-1.24	-15.64
1-Chloropropane	2.71	3.8	-1.09	-40.22
1-Chlorotridecane	30.71	30.63	0.08	0.26
1-Chloroundecane	25.91	25.26	0.65	2.51
1-Decene	15.07	15.5	-0.43	-2.85
1-Decylazide	24.96	26.23	-1.27	-5.09
1-Difluoromethoxy-1,1,2-trifluoroethane	1.78	1.75	0.03	1.69
1-Dodecanol	33.9	33.02	0.88	2.60
1-Dodecene	20.45	20.87	-0.42	-2.05
1-Ethoxy-1,1,2,2,3,3,3-heptafluoropropane	2.61	3.06	-0.45	-17.24
1-Ethoxy-1,1,2,2,3,3,4,4,4-nonafluorobutane	4.98	5.17	-0.19	-3.82
1-Ethoxy-1,1,2,2,3,3,4,4,5,5,5-undecafluoropentane	7.48	7.28	0.20	2.67
1-Ethoxy-1,1,2,2,3,4,4,4-octafluoro-3-trifluoromethylbutane	7.38	7.17	0.21	2.85
1-Ethoxy-1,1,2,2-tetrafluoroethane	3.1	2.72	0.38	12.26
1-Ethyl-1-methylcyclopentane	9.13	8.42	0.71	7.78
1-Ethyl-2-isopropylbenzene	17.01	18.13	-1.12	-6.58
1-Ethyl-3-isopropylbenzene	17.01	18.13	-1.12	-6.58
1-Ethyl-4-isopropylbenzene	17.52	18.13	-0.61	-3.48
1-Ethylnaphthalene	26.26	27.03	-0.77	-2.93
1-Ethylpyrazole	11.59	12.3	-0.71	-6.13
1-Ethylthiooctane	22.39	23.21	-0.82	-3.66
1-Fluorobutane	0.68	1.46	-0.78	-114.71
1-Fluorodecane	17.58	17.56	0.02	0.11
1-Fluorohexane	6.62	6.83	-0.21	-3.17
1-Fluorononane	14.84	14.87	-0.03	-0.20
1-Fluorooctane	12.04	12.19	-0.15	-1.25
1-Fluoropentane	3.54	4.14	-0.60	-16.95
1-Fluoropropane	-2.4	-1.22	-1.18	49.17

1-Heneicosanol	56.11	57.17	-1.06	-1.89
1-Heptadecanol	46.63	46.44	0.19	0.41
1-Heptanethiol	15.75	16.13	-0.38	-2.41
1-Heptanol	20.26	19.61	0.65	3.21
1-Heptene	6.51	7.45	-0.94	-14.44
1-Heptyne	7.2	7.28	-0.08	-1.11
1-Hexadecanol	43.52	43.76	-0.24	-0.55
1-Hexanethiol	12.73	13.45	-0.72	-5.66
1-Hexanol	16.64	16.93	-0.29	-1.74
1-Hexene	4.74	4.77	-0.03	-0.63
1-Hexyne	4.28	4.6	-0.32	-7.48
1H-Perfluorooctane	9.52	8.42	1.10	11.55
1-Iodobutane	9.96	10.71	-0.75	-7.53
1-Iododecane	27	26.8	0.20	0.74
1-Iodododecane	33.28	32.17	1.11	3.34
1-Iodoheptane	18.44	18.75	-0.31	-1.68
1-Iodohexane	15.58	16.07	-0.49	-3.15
1-Iodononane	24.14	24.12	0.02	0.08
1-Iodooctane	21.29	21.44	-0.15	-0.70
1-Iodopentane	12.78	13.39	-0.61	-4.77
1-Iodoundecane	30.55	29.49	1.06	3.47
1-Isobutylimidazole	22.98	23.66	-0.68	-2.96
1-Isopropylimidazole	21.55	20.7	0.85	3.94
1-Methoxy-2-propanol	10.19	11.45	-1.26	-12.37
1-Methyl-1H-pyrazole	10.44	9.96	0.48	4.60
1-Methyl-3-phenoxybenzene	28.62	28.69	-0.07	-0.24
1-Methyl-4-benzylbenzene	29.34	29.31	0.03	0.10
1-Methyl-4-isopropylcyclohexene	15.7	18.18	-2.48	-15.80
1-Methyl-4-vinylcyclohexene	12.84	16.02	-3.18	-24.77
1-Methylfluorene	35.14	39.02	-3.88	-11.04
1-Methylnaphthalene	23.06	24.35	-1.29	-5.59
1-Methylphenanthrene	38.53	38.62	-0.09	-0.23
1-Methylpiperazine	11.64	10.71	0.93	7.99
1-Naphthylamine	32.97	34	-1.03	-3.12
1-Nitro-2,6-diisopropylbenzene	28.02	30.97	-2.95	-10.53
1-Nitro-2-isopropylbenzene	23.98	25.6	-1.62	-6.76
1-Nitrobutane	13.13	13.16	-0.03	-0.23
1-Nitronaphthalene	32.54	34.51	-1.97	-6.05
1-Nitropentane	15.6	15.84	-0.24	-1.54
1-Nitropropane	9.36	10.48	-1.12	-11.97
1-Nitropyrene	64.13	60.99	3.14	4.90
1-Nonadecanol	51.17	51.8	-0.63	-1.23

1-Nonanol	25.74	24.98	0.76	2.95
1-Nonene	12.21	12.82	-0.61	-5.00
1-Nonyne	13.2	12.65	0.55	4.17
1-Octadecanol	48.85	49.12	-0.27	-0.55
1-Octanethiol	18.37	18.81	-0.44	-2.40
1-Octanol	22.55	22.29	0.26	1.15
1-Octyne	10.1	9.96	0.14	1.39
1-Pentadecanol	41.3	41.07	0.23	0.56
1-Pentadecene	29.19	28.92	0.27	0.92
1-Pentanethiol	9.94	10.76	-0.82	-8.25
1-Pentanol	14.18	14.25	-0.07	-0.49
1-Pentene	1.65	2.09	-0.44	-26.67
1-Pentyne	1.38	1.92	-0.54	-39.13
1-Phenyldodecane	38.48	39.59	-1.11	-2.88
1-Phenylimidazole	30.78	30.29	0.49	1.59
1-Phenylnaphthalene	42.81	39.42	3.39	7.92
1-Piperazineethanamine	23.36	19.95	3.41	14.60
1-Propanethiol	5.09	5.4	-0.31	-6.09
1-Propanol	8.9	8.88	0.02	0.22
1-Propoxy-2-propanol	15.07	15.73	-0.66	-4.38
1-Propylamine	3.9	4.6	-0.70	-17.95
1-Pyrenecarboxaldehyde	58.63	56.77	1.86	3.17
1-s-Butylimidazole	23.13	23.38	-0.25	-1.08
1-t-Butyl-4-ethylbenzene	21.29	19.95	1.34	6.29
1-t-Butylimidazole	22.14	21.55	0.59	2.66
1-Tetradecene	26.45	26.23	0.22	0.83
1-Tetradecylamine	31.56	34.11	-2.55	-8.08
1-trans-5-trans-9-cis-Cyclododecatriene	22.49	25.38	-2.89	-12.85
1-Tridecanol	36.38	35.71	0.67	1.84
1-Tridecene	23.17	23.55	-0.38	-1.64
1-Undecanol	30.76	30.34	0.42	1.37
1-Undecene	17.12	18.18	-1.06	-6.19
2,2,2-Trifluoroethanol	5.88	5.68	0.20	3.40
2,2',3,3',4,4',5,5',6-Nonachlorobiphenyl	56.92	58.03	-1.11	-1.95
2,2',3,3',4,4',5,5',6-Nonachlorodiphenyl ether	58.03	60.08	-2.05	-3.53
2,2',3,3',4,4',5,5'-Octachlorobiphenyl	55.36	54.37	0.99	1.79
2,2',3,3',4,4',5,5'-Octachlorodiphenyl ether	55.74	56.43	-0.69	-1.24
2,2',3,3',4,4',5,6,6'-Nonachlorobiphenyl	54.25	58.03	-3.78	-6.97
2,2',3,3',4,4',5,6'-Octachlorobiphenyl	54.03	54.37	-0.34	-0.63
2,2',3,3',4,4',5,6-Octachlorobiphenyl	55.17	54.37	0.80	1.45
2,2',3,3',4,4',5,6'-Octachlorodiphenyl ether	57.42	56.43	0.99	1.72
2,2',3,3',4,4',5-Heptachlorobiphenyl	51.79	50.72	1.07	2.07

2,2',3,3',4,4',6,6'-Octachlorobiphenyl	51.63	54.37	-2.74	-5.31
2,2',3,3',4,4',6,6'-Octachlorodiphenyl ether	55.73	56.43	-0.70	-1.26
2,2',3,3',4,4',6-Heptachlorobiphenyl	49.85	50.72	-0.87	-1.75
2,2',3,3',4,4'-Hexachlorobiphenyl	48.24	47.07	1.17	2.43
2,2',3,3',4,4'-Hexachlorodiphenyl ether	51.75	49.12	2.63	5.08
2,2',3,3',4,5,5',6,6'-Nonachlorobiphenyl	59.53	58.03	1.50	2.52
2,2',3,3',4,5,5',6'-Octachlorobiphenyl	53.12	54.37	-1.25	-2.35
2,2',3,3',4,5,5'-Heptachlorobiphenyl	50.55	50.72	-0.17	-0.34
2,2',3,3',4,5,6,6'-Octachlorobiphenyl	53.69	54.37	-0.68	-1.27
2,2',3,3',4,5',6,6'-Octachlorobiphenyl	52.69	54.37	-1.68	-3.19
2,2',3,3',4,5,6'-Heptachlorobiphenyl	50.97	50.72	0.25	0.49
2,2',3,3',4,5',6'-Heptachlorobiphenyl	50.61	50.72	-0.11	-0.22
2,2',3,3',4,5,6-Heptachlorobiphenyl	52.87	50.72	2.15	4.07
2,2',3,3',4,5',6-Heptachlorobiphenyl	47.98	50.72	-2.74	-5.71
2,2',3,3',4,5'-Hexachlorobiphenyl	47.25	47.07	0.18	0.38
2,2',3,3',4,5-Hexachlorobiphenyl	47.07	47.07	0.00	0.00
2,2',3,3',4,6,6'-Heptachlorobiphenyl	47.87	50.72	-2.85	-5.95
2,2',3,3',4,6'-Hexachlorobiphenyl	47.12	47.07	0.05	0.11
2,2',3,3',4,6-Hexachlorobiphenyl	46.61	47.07	-0.46	-0.99
2,2',3,3',4-Pentabromodiphenyl ether	53.23	53.74	-0.51	-0.96
2,2',3,3',4-Pentachlorobiphenyl	43.98	43.41	0.57	1.30
2,2',3,3',5,5',6,6'-Octachlorobiphenyl	54.48	54.37	0.11	0.20
2,2',3,3',5,5',6-Heptachlorobiphenyl	47.64	50.72	-3.08	-6.47
2,2',3,3',5,5'-Hexachlorobiphenyl	46.26	47.07	-0.81	-1.75
2,2',3,3',5,6,6'-Heptachlorobiphenyl	47.41	50.72	-3.31	-6.98
2,2',3,3',5,6'-Hexachlorobiphenyl	46.44	47.07	-0.63	-1.36
2,2',3,3',5,6-Hexachlorobiphenyl	47.5	47.07	0.43	0.91
2,2',3,3',5-Pentachlorobiphenyl	42.98	43.41	-0.43	-1.00
2,2',3,3',6,6'-Hexachlorobiphenyl	47.27	47.07	0.20	0.42
2,2',3,3',6-Pentachlorobiphenyl	42.44	43.41	-0.97	-2.29
2,2',3,3'-Tetrachlorobiphenyl	40.04	39.76	0.28	0.70
2,2,3,3-Tetramethylhexane	12.99	11.79	1.20	9.24
2,2,3,3-Tetramethylpentane	10.79	9.11	1.68	15.57
2,2',3,4,4',5,5',6-Octachlorobiphenyl	54.03	54.37	-0.34	-0.63
2,2',3,4,4',5,5'-Heptachlorobiphenyl	51.51	50.72	0.79	1.53
2,2',3,4,4',5,6,6'-Octachlorobiphenyl	50.61	54.37	-3.76	-7.43
2,2',3,4,4',5,6-Heptachlorobiphenyl	48.75	50.72	-1.97	-4.04
2,2',3,4,4',5',6-Heptachlorobiphenyl	49.69	50.72	-1.03	-2.07
2,2',3,4,4',5,6'-Heptachlorobiphenyl	49.52	50.72	-1.20	-2.42
2,2',3,4,4',5,6'-Heptachlorodiphenyl ether	51.4	52.77	-1.37	-2.67
2,2',3,4,4',5'-Hexachlorobiphenyl	47.61	47.07	0.54	1.13
2,2',3,4,4',5-Hexachlorobiphenyl	48.09	47.07	1.02	2.12

2,2',3,4,4',5'-Hexachlorodiphenyl ether	50.09	49.12	0.97	1.94
2,2',3,4,4',5-Hexachlorodiphenyl ether	49.69	49.12	0.57	1.15
2,2',3,4,4',6,6'-Heptachlorodiphenyl ether	50.71	52.77	-2.06	-4.06
2,2',3,4,4',6'-Hexachlorobiphenyl	45.13	47.07	-1.94	-4.30
2,2',3,4,4',6-Hexachlorobiphenyl	45.13	47.07	-1.94	-4.30
2,2',3,4,4',6'-Hexachlorodiphenyl ether	48.95	49.12	-0.17	-0.35
2,2',3,4,4'-Pentabromodiphenyl ether	54.55	53.74	0.81	1.48
2,2',3,4,4'-Pentachlorobiphenyl	43.6	43.41	0.19	0.44
2,2',3,4,4'-Pentachlorodiphenyl ether	46.61	45.47	1.14	2.45
2,2',3,4,5,5',6-Heptabromodiphenyl ether	64.7	64.36	0.34	0.53
2,2',3,4,5,5',6-Heptachlorobiphenyl	53.25	50.72	2.53	4.75
2,2',3,4',5,5',6-Heptachlorobiphenyl	48.64	50.72	-2.08	-4.28
2,2',3,4,5,5'-Hexachlorobiphenyl	47.41	47.07	0.34	0.72
2,2',3,4',5,5'-Hexachlorobiphenyl	46.48	47.07	-0.59	-1.27
2,2',3,4,5,6,6'-Heptachlorobiphenyl	47.58	50.72	-3.14	-6.60
2,2',3,4',5,6'-Hexachlorobiphenyl	44.1	47.07	-2.97	-6.73
2,2',3,4,5,6-Hexachlorobiphenyl	48.74	47.07	1.67	3.43
2,2',3,4',5,6-Hexachlorobiphenyl	51.1	47.07	4.03	7.89
2,2',3,4,5',6-Hexachlorobiphenyl	44.44	47.07	-2.63	-5.92
2,2',3,4',5',6-Hexachlorobiphenyl	46.1	47.07	-0.97	-2.10
2,2',3,4,5,6'-Hexachlorobiphenyl	46.44	47.07	-0.63	-1.36
2,2',3,4,5-Pentachlorobiphenyl	43.08	43.41	-0.33	-0.77
2,2',3,4',5-Pentachlorobiphenyl	43.41	43.41	0.00	0.00
2,2',3,4,5'-Pentachlorobiphenyl	43.68	43.41	0.27	0.62
2,2',3,4,6'-Pentachlorobiphenyl	41.6	43.41	-1.81	-4.35
2,2',3,4',6'-Pentachlorobiphenyl	41.21	43.41	-2.20	-5.34
2,2',3,4,6-Pentachlorobiphenyl	42.9	43.41	-0.51	-1.19
2,2',3,4',6-Pentachlorobiphenyl	42.39	43.41	-1.02	-2.41
2,2',3,4'-Tetrachlorobiphenyl	39.32	39.76	-0.44	-1.12
2,2',3,4-Tetrachlorobiphenyl	39.46	39.76	-0.30	-0.76
2,2,3,4-Tetramethylpentane	10.1	8.48	1.62	16.04
2,2',3,5,5',6-Hexachlorobiphenyl	44.1	47.07	-2.97	-6.73
2,2',3,5,5'-Pentachlorobiphenyl	42.3	43.41	-1.11	-2.62
2,2',3,5',6-Pentachlorobiphenyl	41.55	43.41	-1.86	-4.48
2,2',3,5'-Tetrachlorobiphenyl	39.4	39.76	-0.36	-0.91
2,2',3,5-Tetrachlorobiphenyl	38.49	39.76	-1.27	-3.30
2,2',3,6'-Tetrachlorobiphenyl	38.9	39.76	-0.86	-2.21
2,2',3,6-Tetrachlorobiphenyl	37.97	39.76	-1.79	-4.71
2,2',3-Trichlorobiphenyl	36.39	36.11	0.28	0.77
2,2,3-Trimethylpentane	7.85	7.28	0.57	7.26
2,2',4,4',5,5'-Hexabromodiphenyl ether	58.46	59.05	-0.59	-1.01
2,2',4,4',5,5'-Hexachlorobiphenyl	47	47.07	-0.07	-0.15

2,2',4,4',5,5'-Hexachlorodiphenyl ether	48.32	49.12	-0.80	-1.66
2,2',4,4',5,6'-Hexabromodiphenyl ether	59.51	59.05	0.46	0.77
2,2',4,4',5,6'-Hexachlorobiphenyl	44.37	47.07	-2.70	-6.09
2,2',4,4',5,6'-Hexachlorodiphenyl ether	46.78	49.12	-2.34	-5.00
2,2',4,4',5-Pentabromodiphenyl ether	53.12	53.74	-0.62	-1.17
2,2',4,4',5-Pentachlorobiphenyl	43.06	43.41	-0.35	-0.81
2,2',4,4',5-Pentachlorodiphenyl ether	44.95	45.47	-0.52	-1.16
2,2',4,4',6,6'-Hexabromobiphenyl	57.65	57	0.65	1.13
2,2',4,4',6,6'-Hexachlorobiphenyl	46.09	47.07	-0.98	-2.13
2,2',4,4',6-Pentachlorodiphenyl ether	43.76	45.47	-1.71	-3.91
2,2',4,4'-Tetrabromodiphenyl ether	49.13	48.44	0.69	1.40
2,2',4,4'-Tetrachlorobiphenyl	39.65	39.76	-0.11	-0.28
2,2',4,4'-Tetrachlorodiphenyl ether	41.59	41.82	-0.23	-0.55
2,2,4,4-Tetramethylpentane	9.07	9.11	-0.04	-0.44
2,2',4,5,5'-PCB	44.53	43.41	1.12	2.52
2,2',4,5,5'-Pentachlorodiphenyl ether	44.32	45.47	-1.15	-2.59
2,2',4,5',6-Pentachlorobiphenyl	41.19	43.41	-2.22	-5.39
2,2',4,5,6'-Pentachlorobiphenyl	41.02	43.41	-2.39	-5.83
2,2',4,5'-Tetrachlorobiphenyl	39.47	39.76	-0.29	-0.73
2,2',4,5-Tetrachlorobiphenyl	39.64	39.76	-0.12	-0.30
2,2',4,6,6'-Pentachlorobiphenyl	42.06	43.41	-1.35	-3.21
2,2',4,6'-Tetrachlorobiphenyl	37.59	39.76	-2.17	-5.77
2,2',4,6-Tetrachlorobiphenyl	36.36	39.76	-3.40	-9.35
2,2',4-Tribromodiphenyl ether	43.75	43.13	0.62	1.42
2,2',4-Trichlorobiphenyl	36.99	36.11	0.88	2.38
2,2,4-Trimethyl-4-methoxypentane	12.21	11.45	0.76	6.22
2,2,4-Trimethylhexane	9.64	9.96	-0.32	-3.32
2,2',5,5'-Tetrachlorobiphenyl	39.44	39.76	-0.32	-0.81
2,2,5,5-Tetramethylhexane	11.04	11.79	-0.75	-6.79
2,2',5,6'-Tetrachlorobiphenyl	41.74	39.76	1.98	4.74
2,2',5-Trichlorobiphenyl	35.53	36.11	-0.58	-1.63
2,2,5-Trimethylhexane	9.53	9.96	-0.43	-4.51
2,2',6-Trichlorobiphenyl	35.86	36.11	-0.25	-0.70
2,2’-Diaminodiethylamine	21.5	19.04	2.46	11.44
2,2-Dichloro-1,1,1-trifluoroethane	0.34	3.23	-2.89	-850.00
2,2'-Dichlorobiphenyl	31.35	32.45	-1.10	-3.51
2,2'-Dichlorodiisopropyl ether	17.06	16.99	0.07	0.41
2,2-Dimethyl-3-ethylpentane	10.44	9.96	0.48	4.60
2,2-Dimethylheptane	10.33	11.45	-1.12	-10.84
2,2-Dimethylhexane	7.7	8.77	-1.07	-13.90
2,2-Dimethyloctane	13.24	14.13	-0.89	-6.72
2,2-Dimethylpentane	4.96	6.08	-1.12	-22.58

2,2-Dimethylpropane	-1.3	0.72	-2.02	155.38
2,3,3',4,4',5,5',6-Octachlorobiphenyl	54.85	54.37	0.48	0.88
2,3,3',4,4',5,5'-Heptachlorobiphenyl	53.21	50.72	2.49	4.68
2',3,3',4,4',5,6-Heptabromodiphenyl ether	64.21	64.36	-0.15	-0.23
2,3,3',4,4',5,6-Heptachlorobiphenyl	53.06	50.72	2.34	4.41
2,3,3',4,4',5',6-Heptachlorobiphenyl	51.28	50.72	0.56	1.09
2,3,3',4,4',5-Hexabromodiphenyl ether	57.26	59.05	-1.79	-3.13
2,3,3',4,4',5'-Hexachlorobiphenyl	49.7	47.07	2.63	5.29
2,3,3',4,4',5-Hexachlorobiphenyl	49.57	47.07	2.50	5.04
2,3,3',4,4',6-Hexachlorobiphenyl	46.88	47.07	-0.19	-0.41
2,3,3',4,4'-Pentachlorobiphenyl	46.03	43.41	2.62	5.69
2,3,3',4,4'-Pentachlorodiphenyl ether	47.35	45.47	1.88	3.97
2,3,3',4,5,5',6-Heptachlorobiphenyl	51.63	50.72	0.91	1.76
2,3,3',4',5,5',6-Heptachlorobiphenyl	49.8	50.72	-0.92	-1.85
2,3,3',4,5,5'-Hexachlorobiphenyl	45.26	47.07	-1.81	-4.00
2,3,3',4',5,5'-Hexachlorobiphenyl	44.91	47.07	-2.16	-4.81
2,3,3',4,5,6-Hexachlorobiphenyl	48.94	47.07	1.87	3.82
2,3,3',4',5,6-Hexachlorobiphenyl	48.44	47.07	1.37	2.83
2,3,3',4,5',6-Hexachlorobiphenyl	44.91	47.07	-2.16	-4.81
2,3,3',4',5',6-Hexachlorobiphenyl	47.69	47.07	0.62	1.30
2,3,3',4',5'-Pentachlorobiphenyl	46.32	43.41	2.91	6.28
2,3,3',4,5-Pentachlorobiphenyl	44.9	43.41	1.49	3.32
2,3,3',4',5-Pentachlorobiphenyl	45.58	43.41	2.17	4.76
2,3,3',4,5'-Pentachlorobiphenyl	45.62	43.41	2.21	4.84
2,3,3',4',6-Pentachlorobiphenyl	44.2	43.41	0.79	1.79
2,3,3',4'-Tetrachlorobiphenyl	41.63	39.76	1.87	4.49
2,3,3,4-Tetramethylpentane	11.03	8.48	2.55	23.12
2,3,3',5,5',6-Hexachlorobiphenyl	45.06	47.07	-2.01	-4.46
2,3,3',5,5'-Pentachlorobiphenyl	44.6	43.41	1.19	2.67
2,3,3',5,6-Pentachlorobiphenyl	42.84	43.41	-0.57	-1.33
2,3,3',5',6-Pentachlorobiphenyl	43.53	43.41	0.12	0.28
2,3,3',5-Tetrachlorobiphenyl	40.65	39.76	0.89	2.19
2,3,3',6-Tetrachlorobiphenyl	38.9	39.76	-0.86	-2.21
2,3,3'-Trichlorobiphenyl	37.03	36.11	0.92	2.48
2,3,3-Trimethyl-1-butene	4.68	3.97	0.71	15.17
2,3,3-Trimethylhexane	10.44	9.96	0.48	4.60
2,3,3-Trimethylpentane	8.27	7.28	0.99	11.97
2,3',4,4',5,5'-Hexachlorobiphenyl	48.85	47.07	1.78	3.64
2,3',4,4',5,5'-Hexachlorodiphenyl ether	49.35	49.12	0.23	0.47
2,3,4,4',5,6-Hexachlorobiphenyl	45.3	47.07	-1.77	-3.91
2,3',4,4',5',6-Hexachlorobiphenyl	47.23	47.07	0.16	0.34
2,3',4,4',5'-Pentachlorobiphenyl	45.03	43.41	1.62	3.60

2,3,4,4',5-Pentachlorobiphenyl	45.15	43.41	1.74	3.85
2,3',4,4',5-Pentachlorobiphenyl	44.38	43.41	0.97	2.19
2,3',4,4',6-Pentabromodiphenyl ether	55.15	53.74	1.41	2.56
2,3,4,4',6-Pentachlorobiphenyl	43.7	43.41	0.29	0.66
2,3',4,4',6-Pentachlorobiphenyl	43.08	43.41	-0.33	-0.77
2,3',4,4'-Tetrabromodiphenyl ether	49.81	48.44	1.37	2.75
2,3',4,4'-Tetrachlorobiphenyl	41.03	39.76	1.27	3.10
2,3',4,4'-Tetrachlorodiphenyl ether	42.21	41.82	0.39	0.92
2,3',4,5,5'-Pentachlorobiphenyl	44.83	43.41	1.42	3.17
2,3',4',5,5'-Pentachlorobiphenyl	45.42	43.41	2.01	4.43
2,3,4,5,6-Pentabromodiphenyl ether	53.13	53.74	-0.61	-1.15
2,3,4,5,6-Pentachlorobiphenyl	43.53	43.41	0.12	0.28
2,3',4,5',6-Pentachlorobiphenyl	42.47	43.41	-0.94	-2.21
2,3,4',5,6-Pentachlorobiphenyl	43.36	43.41	-0.05	-0.12
2,3',4',5',6-Pentachlorobiphenyl	44.14	43.41	0.73	1.65
2,3,4,5,6-Pentafluorotoluene	9.31	7.22	2.09	22.45
2,3,4,5-Tetrachloroanisole	28.12	25.78	2.34	8.32
2,3',4,5'-Tetrachlorobiphenyl	40.95	39.76	1.19	2.91
2,3',4',5'-Tetrachlorobiphenyl	41.24	39.76	1.48	3.59
2,3,4,5-Tetrachlorobiphenyl	41.3	39.76	1.54	3.73
2,3',4,5-Tetrachlorobiphenyl	41.04	39.76	1.28	3.12
2,3,4',5-Tetrachlorobiphenyl	40.94	39.76	1.18	2.88
2,3',4',5-Tetrachlorobiphenyl	41.38	39.76	1.62	3.91
2,3,4,6,7,8-Hexachlorodibenzofuran	58.84	58.83	0.01	0.02
2,3',4,6-Tetrabromodiphenyl ether	48.52	48.44	0.08	0.16
2,3',4',6-Tetrabromodiphenyl ether	47.91	48.44	-0.53	-1.11
2,3,4,6-Tetrachlorobiphenyl	38.72	39.76	-1.04	-2.69
2,3,4',6-Tetrachlorobiphenyl	40.62	39.76	0.86	2.12
2,3',4',6-Tetrachlorobiphenyl	40.09	39.76	0.33	0.82
2,3,4,6-Tetrachlorophenol	29.99	33.2	-3.21	-10.70
2,3,4,7,8-Pentachlorodibenzofuran	55.19	55.17	0.02	0.04
2',3,4-Tribromodiphenyl ether	44.71	43.13	1.58	3.53
2,3',4-Tribromodiphenyl ether	43.97	43.13	0.84	1.91
2,3,4'-Trichlorobiphenyl	37.41	36.11	1.30	3.48
2,3',4'-Trichlorobiphenyl	37.3	36.11	1.19	3.19
2,3,4-Trichlorobiphenyl	37.31	36.11	1.20	3.22
2,3',4-Trichlorobiphenyl	36.72	36.11	0.61	1.66
2,3,4-Trichlorophenol	28.57	29.54	-0.97	-3.40
2,3,4-Trimethylpentane	8.16	6.65	1.51	18.50
2,3',5,5'-Tetrachlorobiphenyl	40.65	39.76	0.89	2.19
2,3,5,6-Tetrachlorobiphenyl	41.88	39.76	2.12	5.06
2,3',5'-Trichlorobiphenyl	36.28	36.11	0.17	0.47

2,3,5-Trichlorobiphenyl	36.54	36.11	0.43	1.18
2,3',5-Trichlorobiphenyl	36.79	36.11	0.68	1.85
2,3,6-Trichlorobiphenyl	35.14	36.11	-0.97	-2.76
2,3',6-Trichlorobiphenyl	36.05	36.11	-0.06	-0.17
2,3,7,8-Tetrachlorodibenzofuran	50.89	51.52	-0.63	-1.24
2,3,7-Trichlorodibenzo-p-dioxin	49.52	50.61	-1.09	-2.20
2,3,8-Trichlorodibenzofuran	45.93	47.87	-1.94	-4.22
2,3-Butandione	6.45	9.28	-2.83	-43.88
2,3-Butanediol dinitrate	16.99	18.98	-1.99	-11.71
2,3-Butyleneglycol	20.67	21.04	-0.37	-1.79
2,3'-Dichlorobiphenyl	33.04	32.45	0.59	1.79
2,3-Dichlorobiphenyl	33.08	32.45	0.63	1.90
2,3-Dichlorobutane	8.54	8.94	-0.40	-4.68
2,3-Dichlorodibenzo-p-dioxin	48.03	46.95	1.08	2.25
2,3-Dichloronaphthalene	31.3	30.17	1.13	3.61
2,3-Dichloropropene	6.25	7.45	-1.20	-19.20
2,3-Dichlorotoluene	18.61	17.38	1.23	6.61
2,3-Dimethyl-1,3-butadiene	4	1.52	2.48	62.00
2,3-Dimethyl-1-butene	2.73	2.14	0.59	21.61
2,3-Dimethyl-1-hexene	8.21	7.51	0.70	8.53
2,3-Dimethyl-1-pentene	5.59	4.83	0.76	13.60
2,3-Dimethyl-2-butanol	13.58	10.93	2.65	19.51
2,3-Dimethyl-2-pentene	6.79	5	1.79	26.36
2,3-Dimethylbutane	2.85	2.77	0.08	2.81
2,3-Dimethylhexane	8.39	8.14	0.25	2.98
2,3-Dimethylpentane	5.97	5.46	0.51	8.54
2,3-Lutidine	14.02	12.82	1.20	8.56
2,3-Pentadiene	2.17	2.09	0.08	3.69
2,3-Xylenol	25.41	21.55	3.86	15.19
2,4,4',5-Tetrachlorobiphenyl	40.76	39.76	1.00	2.45
2,4,4',5-Tetrachlorodiphenyl ether	41.82	41.82	0.00	0.00
2,4,4',6-Tetrabromodiphenyl ether	48.03	48.44	-0.41	-0.85
2,4,4'-Tribromodiphenyl ether	44.53	43.36	1.17	2.63
2,4,4'-Trichlorobiphenyl	36.95	36.11	0.84	2.27
2,4,4'-Trichlorodiphenyl ether	38.22	38.16	0.06	0.16
2,4,4-Trimethyl-2-pentene	7.48	6.48	1.00	13.37
2,4,4-Trimethylhexane	10.04	9.96	0.08	0.80
2,4,5-T	41.14	40.05	1.09	2.65
2,4,5-Trichlorobiphenyl	36.3	36.11	0.19	0.52
2,4',5-Trichlorobiphenyl	36.91	36.11	0.80	2.17
2,4,5-Trichlorodiphenyl ether	37.37	38.16	-0.79	-2.11
2,4',5-Trichlorodiphenyl ether	37.93	38.16	-0.23	-0.61

2,4,5-Trichlorophenol	26.15	29.54	-3.39	-12.96
2,4,6-Collidine	14.73	14.02	0.71	4.82
2,4,6-Tribromodiphenyl ether	42.28	43.13	-0.85	-2.01
2,4',6-Tribromodiphenyl ether	44.1	43.13	0.97	2.20
2,4,6-Tribromophenol	32.81	34.51	-1.70	-5.18
2,4,6-Trichloroanisole	23.87	22.12	1.75	7.33
2,4,6-Trichlorobiphenyl	36.43	36.11	0.32	0.88
2,4',6-Trichlorobiphenyl	35.64	36.11	-0.47	-1.32
2,4,6-Trichlorodibenzofuran	45.01	47.87	-2.86	-6.35
2,4,6-Trichlorophenol	28.46	29.54	-1.08	-3.79
2,4,6-Tri-t-butylnitrobenzene	43	41.82	1.18	2.74
2,4,8-Trichlorodibenzofuran	44.9	47.87	-2.97	-6.61
2,4'-Dibromodiphenyl ether	39.46	37.82	1.64	4.16
2,4-Dibromodiphenyl ether	38.93	37.82	1.11	2.85
2,4'-Dichlorobiphenyl	33.04	32.45	0.59	1.79
2,4-Dichlorobiphenyl	32.89	32.45	0.44	1.34
2,4-Dichlorodiphenyl ether	33.77	34.51	-0.74	-2.19
2,4-Dichlorophenoxyacetic acid	39.75	36.39	3.36	8.45
2,4-Dichlorotoluene	17.98	17.38	0.60	3.34
2,4-Dimethyl-1-pentene	5.19	4.83	0.36	6.94
2,4-Dimethyl-2-pentene	5.48	4.66	0.82	14.96
2,4-Dimethyl-3-ethylpentane	10.73	9.34	1.39	12.95
2,4-Dimethyl-3-pentanone	9.99	9.91	0.08	0.80
2,4-Dimethylhexane	7.98	8.14	-0.16	-2.01
2,4-Dimethylpentane	5.07	5.46	-0.39	-7.69
2,4-Dinitrophenol	41.46	41.87	-0.41	-0.99
2,4-Di-t-butylphenol	30.35	32.97	-2.62	-8.63
2,4-Lutidine	13.64	12.82	0.82	6.01
2,4-Xylenol	22.24	21.55	0.69	3.10
2,5-Aldehydine	15.47	15.5	-0.03	-0.19
2,5-Dichlorobiphenyl	32.83	32.45	0.38	1.16
2,5-Dichlorotoluene	18.04	17.38	0.66	3.66
2,5-Dimethyl-1,5-hexadiene	9.53	6.88	2.65	27.81
2,5-Dimethylhexane	7.99	8.14	-0.15	-1.88
2,5-Dimethyltetrahydrofuran	6.17	5.11	1.06	17.18
2,5-Dimethylthiophene	11.07	10.88	0.19	1.72
2,5-Lutidine	13.45	12.82	0.63	4.68
2,5-Xylenol	20.89	21.55	-0.66	-3.16
2,5-Xylidine	21.69	22.69	-1.00	-4.61
2,6-Dibromodiphenyl ether	37.66	37.82	-0.16	-0.42
2,6-Dichlorobiphenyl	31.66	32.45	-0.79	-2.50
2,6-Dichlorodiphenyl ether	32.91	34.51	-1.60	-4.86

2,6-Dichloronaphthalene	31.22	30.17	1.05	3.36
2,6-Dichlorophenol	24.92	25.89	-0.97	-3.89
2,6-Dichlorosyringaldehyde	37.72	38.45	-0.73	-1.94
2,6-Dichlorotoluene	18.04	17.38	0.66	3.66
2,6-Diethylaniline	30.29	28.06	2.23	7.36
2,6-Dimethyl-4-heptanol	19.41	19.04	0.37	1.91
2,6-Dimethylheptan-4-one	15.18	15.27	-0.09	-0.59
2,6-Dimethylheptane	10.91	10.82	0.09	0.82
2,6-Dimethylnaphthalene	26.81	25.83	0.98	3.66
2,6-Lutidine	12.16	12.53	-0.37	-3.04
2,6-Xylenol	21	21.55	-0.55	-2.62
2,6-Xylidine	21.55	22.69	-1.14	-5.29
2,7-Dibromofluorene	45.46	48.15	-2.69	-5.92
2,7-Dichloronaphthalene	31.22	30.17	1.05	3.36
2,7-Diiodofluorene	52.84	52.72	0.12	0.23
2,7-Dimethyloctane	13.18	13.5	-0.32	-2.43
2,8-Dichlorodibenzo-p-dioxin	50.57	46.95	3.62	7.16
2-Adamantanone	27.31	28.23	-0.92	-3.37
2-Azidoacetonitrile	14.51	11.62	2.89	19.92
2-Azidoethanol	18.01	15.96	2.05	11.38
2-Azidoethoxyethane	10.78	11.91	-1.13	-10.48
2-Bromo-7-chlorofluorene	42.73	46.5	-3.77	-8.82
2-Bromo-7-iodofluorene	49.13	50.43	-1.30	-2.65
2-Bromoadamantane	25.62	26.35	-0.73	-2.85
2-Bromobutane	6.33	6.83	-0.50	-7.90
2-Bromodiphenyl ether	33.12	32.74	0.38	1.15
2-Bromofluorene	38.53	42.84	-4.31	-11.19
2-Bromonaphthalene	30.29	28.17	2.12	7.00
2-Bromopropane	3.11	4.14	-1.03	-33.12
2-Bromotoluene	15.62	15.39	0.23	1.47
2-Butanethiol	5.56	6.31	-0.75	-13.49
2-Butanol	9.13	8.59	0.54	5.91
2-Butanone	5.08	4.83	0.25	4.92
2-Butyne	0.18	0.6	-0.42	-233.33
2-Chloro-1,1,1,2-tetrafluoroethane	-3.48	-2.08	-1.40	40.23
2-Chloro-1,1,1-trifluoroethane	-1.71	0.6	-2.31	135.09
2-Chloro-1,1,2-trifluoroethyl difluoromethyl ether	3.13	4.09	-0.96	-30.67
2-Chloro-1,1,2-trifluoroethyl ethyl ether	6.37	4.88	1.49	23.39
2-Chloro-1,1,2-trifluoroethyl propyl ether	8.59	7.57	1.02	11.87
2-Chloro-1,1-difluoroethylene	-3.88	-2.99	-0.89	22.94
2-Chloroadamantane	24.04	24.18	-0.14	-0.58
2-Chloroaniline	20.41	23.38	-2.97	-14.55

2-Chlorobiphenyl	28.29	28.8	-0.51	-1.80
2-Chlorobutane	3.82	4.66	-0.84	-21.99
2-Chlorodiphenyl ether	30.11	30.86	-0.75	-2.49
2-Chloroethanol	11.56	12.3	-0.74	-6.40
2-Chloronaphthalene	25.24	26.52	-1.28	-5.07
2-Chloronitrobenzene	24.35	23.89	0.46	1.89
2-Chloropentane	6.83	7.34	-0.51	-7.47
2-Chloropropane	1	1.97	-0.97	-97.00
2-Chloropropanoic acid	19.71	18.3	1.41	7.15
2-Chloropropene	-0.06	1.35	-1.41	2350.00
2-Chloropyridine	14.5	15.22	-0.72	-4.97
2-Chlorosyringaldehyde	34.86	34.79	0.07	0.20
2-Chlorotoluene	13.32	13.73	-0.41	-3.08
2-cis-Decene	15.87	15.33	0.54	3.40
2-cis-Dodecene	21.69	20.7	0.99	4.56
2-Decanol	25.51	24.69	0.82	3.21
2-Diethylaminoethanethiol	14.49	14.76	-0.27	-1.86
2-Difluoromethoxy-1,1,1,3,3,3-hexafluoropropane	4.02	2.6	1.42	35.32
2-Difluoromethoxy-1,1-difluoroethane	3	2.6	0.40	13.33
2-Diisopropylaminoethanethiol	18.34	16.59	1.75	9.54
2-Dimethylaminoethanethiol	10.27	10.08	0.19	1.85
2-Dodecanol	31.06	30.06	1.00	3.22
2-Ethoxy-1,1,1,3,3,3-hexafluoro-2-trifluoromethylpropane	4.02	6.65	-2.63	-65.42
2-Ethoxyethanol	12.21	13.33	-1.12	-9.17
2-Ethoxyethylacetate	14.27	12.65	1.62	11.35
2-Ethyl-1-butanol	15.41	15.44	-0.03	-0.19
2-Ethyl-1-butene	3.63	3.63	0.00	0.00
2-Ethyl-1-hexanol	21.29	20.81	0.48	2.25
2-Ethyl-1-pentene	6.41	6.31	0.10	1.56
2-Ethylfuran	6.39	9.45	-3.06	-47.89
2-Ethylhexyl acetate	19.98	20.12	-0.14	-0.70
2-Ethylhexyl acrylate	20.66	21.84	-1.18	-5.71
2-Ethyl-m-xylene	16.44	15.73	0.71	4.32
2-Ethylnaphthalene	26.24	27.03	-0.79	-3.01
2-Ethylnitrobenzene	23.37	24.41	-1.04	-4.45
2-Ethyl-p-xylene	16.58	15.73	0.85	5.13
2-Ethylpyridine	12.5	14.02	-1.52	-12.16
2-Ethylthiophene	11.11	11.28	-0.17	-1.53
2-Ethyltoluene	14.13	14.25	-0.12	-0.85
2-Fluorenylaldehyde	46.17	44.95	1.22	2.64
2-Fluoroiodobenzene	16.61	15.62	0.99	5.96

2-Fluoronitrobenzene	21.07	19.67	1.40	6.64
2-Heptanol	17.18	16.64	0.54	3.14
2-Heptanone	13.13	12.88	0.25	1.90
2-Hexanol	14.19	13.96	0.23	1.62
2-Hexyne	5.58	5.97	-0.39	-6.99
2-Hydroxy-4-methoxybenzaldehyde	28.43	28.57	-0.14	-0.49
2-Hydroxy-5-methoxybenzaldehyde	26.79	28.74	-1.95	-7.28
2-Hydroxy-6-methoxybenzaldehyde	28.92	28.57	0.35	1.21
2-Imidazolidinone	42.63	41.87	0.76	1.78
2-Iodofluorene	42.2	45.13	-2.93	-6.94
2-Isopropylnaphthalene	29.39	28.23	1.16	3.95
2-Isopropyltoluene	15.35	15.44	-0.09	-0.59
2-Methoxyaniline	22.6	22.29	0.31	1.37
2-Methoxyethylamine	6.71	7.45	-0.74	-11.03
2-Methoxy-p-cresol	25.72	22.64	3.08	11.98
2-Methoxyphenol	22.09	21.15	0.94	4.26
2-Methyl-1,1-diphenylpropane	31.73	32.91	-1.18	-3.72
2-Methyl-1,2-propanediamine	10.78	10.59	0.19	1.76
2-Methyl-1-butanol	13.64	12.76	0.88	6.45
2-Methyl-1-heptene	8.94	8.99	-0.05	-0.56
2-Methyl-1-hexene	6.26	6.31	-0.05	-0.80
2-Methyl-1-octene	11.81	11.68	0.13	1.10
2-Methyl-1-pentanol	15.01	15.44	-0.43	-2.86
2-Methyl-1-pentene	3.42	3.63	-0.21	-6.14
2-Methyl-1-propanethiol	5.93	6.6	-0.67	-11.30
2-Methyl-1-propanol	10.27	10.08	0.19	1.85
2-Methyl-2-butanol	9.47	9.74	-0.27	-2.85
2-Methyl-2-butenenitrile	8.42	7.62	0.80	9.50
2-Methyl-2-pentanol	11.87	12.42	-0.55	-4.63
2-Methyl-2-pentene	3.9	3.46	0.44	11.28
2-Methyl-2-propanol	7.22	7.05	0.17	2.35
2-Methyl-3-butenenitrile	8.94	7.62	1.32	14.77
2-Methyl-3-pentanol	12.27	12.48	-0.21	-1.71
2-Methylacrylonitrile	5.87	5.11	0.76	12.95
2-Methylaminoethanol	16.64	14.07	2.57	15.44
2-Methylanthracene	38.73	38.62	0.11	0.28
2-Methylaziridine	4.68	4.31	0.37	7.91
2-Methylbenzaldehyde	18.29	17.5	0.79	4.32
2-Methyldecane	17.64	17.67	-0.03	-0.17
2-Methylglutaric acid	45.7	46.95	-1.25	-2.74
2-Methylheptane	8.94	9.62	-0.68	-7.61
2-Methylhexane	6.28	6.94	-0.66	-10.51

2-Methylnaphthalene	23.66	24.35	-0.69	-2.92
2-Methylnonane	14.87	14.99	-0.12	-0.81
2-Methyloctane	11.87	12.3	-0.43	-3.62
2-Methylpentane	3.19	4.26	-1.07	-33.54
2-Methylpropanal	3.69	4.83	-1.14	-30.89
2-Methylpropane	-2.11	-1.11	-1.00	47.39
2-Methylpropanoic acid	14.82	15.22	-0.40	-2.70
2-Methylpropylamine	4.2	5.8	-1.60	-38.10
2-Methylpyrazine	11.24	12.88	-1.64	-14.59
2-Methylstyrene	15.01	13.28	1.73	11.53
2-Methyltetrahydrofuran	3.65	3.8	-0.15	-4.11
2-Methylthiophene	8.49	8.59	-0.10	-1.18
2-Nitroaniline	35.09	31.37	3.72	10.60
2-Nitrobutane	11.36	12.02	-0.66	-5.81
2-Nitro-m-xylene	21.48	23.21	-1.73	-8.05
2-Nitropropane	9.45	9.34	0.11	1.16
2-Nitro-t-butylbenzene	24.69	27.43	-2.74	-11.10
2-Nitrotoluene	21.2	21.72	-0.52	-2.45
2-Nonanol	22.7	22.01	0.69	3.04
2-Nonanone	18.43	18.24	0.19	1.03
2-n-Propylphenol	22.49	25.43	-2.94	-13.07
2-n-Propylthiophene	13.62	13.96	-0.34	-2.50
2-Octanol	19.86	19.33	0.53	2.67
2-Octanone	15.7	15.56	0.14	0.89
2-Pentanol	11.93	11.28	0.65	5.45
2-Pentyne	2.91	3.29	-0.38	-13.06
2-Phenylethylamine	19.25	19.67	-0.42	-2.18
2-Picoline	10.44	11.33	-0.89	-8.52
2-Propanethiol	2.5	3.63	-1.13	-45.20
2-Propanol	6.91	5.91	1.00	14.47
2-Propyltoluene	16.49	16.93	-0.44	-2.67
2-Propyn-1-ol	9.53	7.74	1.79	18.78
2-Pyrrolidone	26.03	26.46	-0.43	-1.65
2-t-Butyl-4-methylphenol	26.44	27.26	-0.82	-3.10
2-t-Butyl-5-methylphenol	25.38	27.26	-1.88	-7.41
2-t-Butylanthracene	44.88	44.33	0.55	1.23
2-t-Butylphenol	22.62	25.78	-3.16	-13.97
2-Tetradecanol	37.25	35.42	1.83	4.91
2-t-Pentylphenol	24.65	28.46	-3.81	-15.46
2-Undecanol	28.03	27.37	0.66	2.35
2-Undecanone	24.31	23.61	0.70	2.88
3-(Difluoromethoxy)-1,1,1,2,2-pentafluoropropane	2.02	2.94	-0.92	-45.54

3-(Perfluorobutyl)propanol	17.44	19.5	-2.06	-11.81
3,3,3-Trifluoropropene	-4.29	-3.79	-0.50	11.66
3,3',4,4',5-Pentachlorobiphenyl	47.48	43.41	4.07	8.57
3,3',4,4',5-Pentachlorodiphenyl ether	47.12	45.47	1.65	3.50
3,3',4,4'-Tetrabromodiphenyl ether	50.95	48.44	2.51	4.93
3,3',4,4'-Tetrachlorobiphenyl	43.74	39.76	3.98	9.10
3,3',4,4'-Tetrachlorodiphenyl ether	43.36	41.82	1.54	3.55
3,3',4,5,5'-Pentachlorobiphenyl	46.31	43.41	2.90	6.26
3,3',4,5'-Tetrachlorobiphenyl	42.65	39.76	2.89	6.78
3,3',4,5-Tetrachlorobiphenyl	42.82	39.76	3.06	7.15
3,3',4-Tribromodiphenyl ether	45.35	43.13	2.22	4.90
3,3',4-Trichlorobiphenyl	39.36	36.11	3.25	8.26
3,3',5,5'-Tetrachlorobiphenyl	41.48	39.76	1.72	4.15
3,3',5-Trichlorobiphenyl	38.38	36.11	2.27	5.91
3,3,5-Trimethylheptane	12.9	12.65	0.25	1.94
3,3'-Bitolyl	30.82	28.12	2.70	8.76
3,3'-Dichlorobiphenyl	34.54	32.45	2.09	6.05
3,3-Dimethyl-1-butene	1.43	2.43	-1.00	-69.93
3,3-Dimethyl-1-pentene	4.96	5.11	-0.15	-3.02
3,3-Dimethylhexane	8.16	8.77	-0.61	-7.48
3,3-Dimethylpentane	5.54	6.08	-0.54	-9.75
3,4,4',5-Tetrachlorobiphenyl	43.08	39.76	3.32	7.71
3,4,4'-Tribromodiphenyl ether	46.25	43.13	3.12	6.75
3,4,4'-Trichlorobiphenyl	39.4	36.11	3.29	8.35
3,4,5,6-Tetrachloroguaiacol	33.48	35.76	-2.28	-6.81
3,4,5-Trichlorobiphenyl	39.89	36.11	3.78	9.48
3,4',5-Trichlorobiphenyl	38.24	36.11	2.13	5.57
3,4,5-Trichloroguaiacol	29.68	32.11	-2.43	-8.19
3,4'-Dibromodiphenyl ether	39.97	37.82	2.15	5.38
3,4-Dibromodiphenyl ether	39.82	37.82	2.00	5.02
3,4-Dichloroaniline	27.28	27.03	0.25	0.92
3,4'-Dichlorobiphenyl	35.57	32.45	3.12	8.77
3,4-Dichlorobiphenyl	35.84	32.45	3.39	9.46
3,4-Dichloronitrobenzene	27.72	27.55	0.17	0.61
3,4-Dichlorophenol	26.43	25.89	0.54	2.04
3,4-Dichlorotoluene	18.61	17.38	1.23	6.61
3,4-Dimethylhexane	8.82	8.14	0.68	7.71
3,4-Lutidine	15.18	13.1	2.08	13.70
3,4-Xylidine	25.38	22.69	2.69	10.60
3,5,5-Trimethyl-2-cyclohexenone	18.89	22.52	-3.63	-19.22
3,5-Dichlorobiphenyl	33.71	32.45	1.26	3.74
3,5-Dichlorosyringol	30.47	31.03	-0.56	-1.84

3,5-Lutidine	15.1	13.1	2.00	13.25
3-Bromochlorobenzene	17.17	17.56	-0.39	-2.27
3-Bromodiphenyl ether	33.73	32.51	1.22	3.62
3-Bromopyridine	14.95	15.44	-0.49	-3.28
3-Bromotoluene	16.21	15.39	0.82	5.06
3-Chloroaniline	22.32	23.38	-1.06	-4.75
3-Chlorobiphenyl	28.58	28.8	-0.22	-0.77
3-Chloronitrobenzene	24.03	23.89	0.14	0.58
3-Chlorophenol	21.61	22.24	-0.63	-2.92
3-Chlorostyrene	16.44	15.44	1.00	6.08
3-Chlorosyringol	29.05	27.37	1.68	5.78
3-Chlorotoluene	13.22	13.73	-0.51	-3.86
3-Difluoromethoxy-1,1,2,2-tetrafluoropropane	5.03	4.71	0.32	6.36
3-Ethyl-1-pentene	5.54	5.97	-0.43	-7.76
3-Ethyl-2-methylpentane	8.58	8.14	0.44	5.13
3-Ethyl-3-methylpentane	8.67	8.77	-0.10	-1.15
3-Ethylheptane	9.64	12.3	-2.66	-27.59
3-Ethylhexane	9.01	9.62	-0.61	-6.77
3-Ethyl-o-xylene	17.52	15.73	1.79	10.22
3-Ethylpentane	6.39	6.94	-0.55	-8.61
3-Ethylphenol	23.76	22.75	1.01	4.25
3-Ethylpyridine	14.19	14.3	-0.11	-0.78
3-Ethyltoluene	13.57	14.25	-0.68	-5.01
3-Fluorobenzotrifluoride	7.4	8.99	-1.59	-21.49
3-Fluoroiodobenzene	16.09	15.62	0.47	2.92
3-Fluoronitrobenzene	18.52	19.67	-1.15	-6.21
3-Heptanol	17.18	16.64	0.54	3.14
3-Heptanone	13.98	12.88	1.10	7.87
3-Hexanol	13.93	13.96	-0.03	-0.22
3-Hexanone	9.93	10.19	-0.26	-2.62
3-Hexyne	5.36	5.97	-0.61	-11.38
3-Hydroxybenzaldehyde	27.09	26	1.09	4.02
3-Isopropyltoluene	15.41	15.44	-0.03	-0.19
3-Methoxysalicylaldehyde	28.78	28.57	0.21	0.73
3-Methyl-1,2-butadiene	1.43	1.12	0.31	21.68
3-Methyl-1-butene	-0.37	0.6	-0.97	262.16
3-Methyl-1-butyne	0.36	0.43	-0.07	-19.44
3-Methyl-1-hexene	5.51	5.97	-0.46	-8.35
3-Methyl-1-pentene	2.62	3.29	-0.67	-25.57
3-Methyl-2-butanol	10.96	9.79	1.17	10.68
3-Methyl-2-butanone	6.62	6.03	0.59	8.91
3-Methyl-2-butenyl acetate	13.33	11.28	2.05	15.38

3-Methyl-2-pentanol	13.53	12.48	1.05	7.76
3-Methyl-3-pentanol	12.21	12.42	-0.21	-1.72
3-Methylbenzaldehyde	19.14	17.5	1.64	8.57
3-Methylbutanoic acid	18.38	17.9	0.48	2.61
3-Methylcholanthrene	61.85	65.33	-3.48	-5.63
3-Methyl-cis-2-pentene	3.99	3.46	0.53	13.28
3-Methylcyclopentene	9.13	9.96	-0.83	-9.09
3-Methyleneheptane	9.02	8.99	0.03	0.33
3-Methylglutaric acid	45.9	46.95	-1.05	-2.29
3-Methylheptane	9.13	9.62	-0.49	-5.37
3-Methylhexane	6.28	6.94	-0.66	-10.51
3-Methylnonane	14.75	14.99	-0.24	-1.63
3-Methyloctane	11.81	12.3	-0.49	-4.15
3-Methylpentane	3.45	4.26	-0.81	-23.48
3-Methylpyridine	12.02	11.62	0.40	3.33
3-Methylstyrene	14.86	13.28	1.58	10.63
3-Methylthiophene	8.73	7.85	0.88	10.08
3-Methyl-trans-2-pentene	4.2	3.46	0.74	17.62
3-Methylundecane	20.52	20.35	0.17	0.83
3-Nitrobenzotrifluoride	19.81	21.21	-1.40	-7.07
3-Nitrotoluene	21.99	21.72	0.27	1.23
3-Nonanol	21.85	22.01	-0.16	-0.73
3-Octanol	19.22	19.33	-0.11	-0.57
3-Octanone	14.55	15.56	-1.01	-6.94
3-Pentanol	11.11	11.28	-0.17	-1.53
3-Propyltoluene	16.11	16.93	-0.82	-5.09
3-t-Butylphenol	28.2	25.78	2.42	8.58
4,4,6-Trimethyl-1,3-dioxane	11.97	12.7	-0.73	-6.10
4,4'-Dibromodiphenyl ether	40.31	37.82	2.49	6.18
4,4'-Dichlorobiphenyl	35.07	32.45	2.62	7.47
4,4-Dimethyl-1-pentene	4.28	5.11	-0.83	-19.39
4,5,6-Trichloroguaiacol	32.02	32.11	-0.09	-0.28
4,5-Dichloroguaiacol	27.5	28.46	-0.96	-3.49
4,5-Dimethylphenanthrene	39.47	40.1	-0.63	-1.60
4-Acetoxyacetophenone	29.29	28.57	0.72	2.46
4-Allyl-2-methoxyphenol	27.27	27.03	0.24	0.88
4-Amino-3,5,6-trichloro-2-pyridinecarboxlic acid	51.88	51.18	0.70	1.35
4-Bromodiphenyl ether	31.92	32.51	-0.59	-1.85
4-Bromophenol	25.4	23.89	1.51	5.94
4-Bromotoluene	16.13	15.39	0.74	4.59
4-Chloroaniline	25.43	23.38	2.05	8.06
4-Chlorobiphenyl	28.81	28.8	0.01	0.03

4-Chloroiodobenzene	21.66	19.84	1.82	8.40
4-Chloro-m-cresol	23.87	23.72	0.15	0.63
4-Chloronitrobenzene	25.95	23.89	2.06	7.94
4-Chlorophenol	22.47	22.24	0.23	1.02
4-Chlorotoluene	13.93	13.73	0.20	1.44
4-Cumylphenyl diphenyl phosphate	78.59	78.8	-0.21	-0.27
4-Ethyl-m-xylene	16.73	15.73	1.00	5.98
4-Ethyl-o-xylene	16.38	15.73	0.65	3.97
4-Ethylphenol	24.61	22.75	1.86	7.56
4-Ethylpyridine	14.42	14.3	0.12	0.83
4-Ethyltoluene	13.75	14.25	-0.50	-3.64
4-Fluoroiodobenzene	15.92	15.62	0.30	1.88
4-Fluoronitrobenzene	19.17	19.67	-0.50	-2.61
4-Fluorophenol	18.83	18.01	0.82	4.35
4-Heptanol	16.55	16.64	-0.09	-0.54
4-Heptanone	15.92	12.88	3.04	19.10
4-Iodophenol	26.71	26.18	0.53	1.98
4-Methyl-1-hexene	5.76	5.97	-0.21	-3.65
4-Methyl-1-pentanol	16.89	15.44	1.45	8.58
4-Methyl-1-pentene	2.57	3.29	-0.72	-28.02
4-Methyl-2-pentanol	12.36	12.48	-0.12	-0.97
4-Methylbenzaldehyde	19.81	17.5	2.31	11.66
4-Methyl-cis-2-pentene	2.82	3.11	-0.29	-10.28
4-Methylcyclopentene	9.02	9.96	-0.94	-10.42
4-Methylheptane	8.96	9.62	-0.66	-7.37
4-Methylnonane	14.36	14.99	-0.63	-4.39
4-Methyloctane	11.68	12.3	-0.62	-5.31
4-Methylpent-3-en-2-one	10.5	7.91	2.59	24.67
4-Methylpyridine	12.04	11.62	0.42	3.49
4-Methylstyrene	14.95	13.28	1.67	11.17
4-Methyl-trans-2-pentene	3.08	3.11	-0.03	-0.97
4-Nitrotoluene	20.82	21.72	-0.90	-4.32
4-Octylphenol	35.13	38.85	-3.72	-10.59
4-Propylphenol	23.57	25.43	-1.86	-7.89
4-Propyltoluene	16.2	16.93	-0.73	-4.51
4-t-Butylphenol	25.38	25.78	-0.40	-1.58
5-Ethyl-m-xylene	17.92	15.73	2.19	12.22
5-Isopropyl-2-methylphenol	25.46	25.43	0.03	0.12
5-Methyl-1-hexene	5.65	5.97	-0.32	-5.66
5-Methylnonane	14.49	14.99	-0.50	-3.45
5-Nonanone	17.76	18.24	-0.48	-2.70
6-Methyl-1-heptene	10.9	8.65	2.25	20.64

7,12-Dimethylbenz[a]anthracene	58.23	54.37	3.86	6.63
9,10-Benzophenanthrene	50.75	51.4	-0.65	-1.28
9,10-Dihydroanthracene	37.04	40.9	-3.86	-10.42
9,10-Diphenylanthracene	72.67	70.24	2.43	3.34
Acenaphthene	31.38	35.31	-3.93	-12.52
Acenaphthylene	33.71	37.48	-3.77	-11.18
Acetaldehyde	-0.4	0.95	-1.35	337.50
Acetamide	17.21	15.44	1.77	10.28
Acetic acid	10.27	11.33	-1.06	-10.32
Acetol	12.89	13.33	-0.44	-3.41
Acetonitrile	5.31	1.86	3.45	64.97
Acetophenone	18.75	17.21	1.54	8.21
Acetylacetone	11.41	11.96	-0.55	-4.82
Acetylchloride	0.85	1.46	-0.61	-71.76
Acrylic acid	12.84	13.05	-0.21	-1.64
Adamantane	21.4	19.9	1.50	7.01
Adiponitrile	16.61	19.44	-2.83	-17.04
Allyl acetate	7.62	7.22	0.40	5.25
Allyl alcohol	8.37	7.91	0.46	5.50
Allyl chloride	1.82	2.83	-1.01	-55.49
Allyl cyanide	9.22	6.25	2.97	32.21
Allyl hexanoate	17.65	17.96	-0.31	-1.76
Allyl sulfide	10.79	10.54	0.25	2.32
Allylamine	2.74	3.63	-0.89	-32.48
alpha,alpha-Dimethylphenethylamine	19.98	21.44	-1.46	-7.31
alpha-Curcumene	28.34	26.57	1.77	6.25
alpha-Ionone	26.72	28.69	-1.97	-7.37
alpha-Methyl benzylamine	17.64	17.9	-0.26	-1.47
alpha-Methylstyrene	14.5	13.33	1.17	8.07
alpha-Pinene	12.79	14.59	-1.80	-14.07
Alverine	44.86	47.92	-3.06	-6.82
Ametryn	48.2	48.78	-0.58	-1.20
Amyl acetate	13.39	13.56	-0.17	-1.27
Amyl propionate	13.28	16.24	-2.96	-22.29
Amylamine	9.61	9.96	-0.35	-3.64
Amylbenzene	19.18	20.81	-1.63	-8.50
Aniline	17.41	19.73	-2.32	-13.33
Anthracene	35.31	37.13	-1.82	-5.15
Arachic alcohol	53.56	54.49	-0.93	-1.74
Atrazine	53.68	53.23	0.45	0.84
Azepane	10.98	9.79	1.19	10.84
Azetidine	3.64	3.69	-0.05	-1.37

Azidocyclopentane	10.58	10.71	-0.13	-1.23
Aziridine	3.08	3.4	-0.32	-10.39
Azocane	13.67	10.54	3.13	22.90
Barban	53.1	53.23	-0.13	-0.24
Benz[a]anthracene	49.86	51.4	-1.54	-3.09
Benz[e]acephenanthrylene	62.82	64.08	-1.26	-2.01
Benzalchloride	18.21	18.81	-0.60	-3.29
Benzaldehyde	15.87	16.02	-0.15	-0.95
Benzene	5.17	8.59	-3.42	-66.15
Benzo[a]fluorene	51.15	51.8	-0.65	-1.27
Benzo[a]phenanthrene	49.98	51.4	-1.42	-2.84
Benzo[a]pyrene	63.59	63.62	-0.03	-0.05
Benzo[b]fluorene	48.58	51.8	-3.22	-6.63
Benzo[b]naphtho[2,3-d]thiophene	47.92	47.75	0.17	0.35
Benzo[b]triphenylene	65.46	65.67	-0.21	-0.32
Benzo[e]pyrene	63.41	63.62	-0.21	-0.33
Benzo[f]quinoline	40.71	39.59	1.12	2.75
Benzo[ghi]perylene	74.24	75.83	-1.59	-2.14
Benzonitrile	17.16	16.93	0.23	1.34
Benzophenone	33.57	32.28	1.29	3.84
Benzotrifluoride	7.42	9.57	-2.15	-28.98
Benzoyl chloride	17.61	16.53	1.08	6.13
Benzphetamine	36.55	38.45	-1.90	-5.20
Benzyl acetate	20.75	20.58	0.17	0.82
Benzyl alcohol	21.86	21.27	0.59	2.70
Benzyl benzoate	37.21	35.65	1.56	4.19
Benzyl butyl phthalate	52.63	53	-0.37	-0.70
Benzyl ethyl ether	16.64	17.21	-0.57	-3.43
Benzylamine	17.29	16.99	0.30	1.74
beta-Citronellol	26.83	23.89	2.94	10.96
beta-HCH	31.85	32.57	-0.72	-2.26
beta-Phellandrene	15.35	19.15	-3.80	-24.76
beta-Sesquiphellandrene	27.57	30.29	-2.72	-9.87
Bicifadine	27.94	26.29	1.65	5.91
Bicyclohexyl	21.97	21.15	0.82	3.73
Bifenox	58.64	56.54	2.10	3.58
Biphenyl	24.95	25.15	-0.20	-0.80
Biphenylene	31	31.66	-0.66	-2.13
Bis(2-chloroethyl) ether	15.35	14.36	0.99	6.45
Bis(3-aminopropyl)amine	25.1	24.41	0.69	2.75
Bis(perfluorobutyl)disulfide	13.96	14.87	-0.91	-6.52
Bistrifluoromethyl disulfide	0.9	1.17	-0.27	-30.00

Bromobenzene	13.01	13.9	-0.89	-6.84
Bromocyclohexane	13.66	13.33	0.33	2.42
Bromoethane	2.3	2.66	-0.36	-15.65
Bromoethylene	-0.69	1.17	-1.86	269.57
Butanal	4.79	6.31	-1.52	-31.73
Butane	-2	0.38	-2.38	119.00
Butanedioic acid	42.9	43.07	-0.17	-0.40
Butanoic acid	16.49	16.7	-0.21	-1.27
Butyl 1,1-dimethylpropyl ether	12.66	12.7	-0.04	-0.32
Butyl 4-oxopentanoate	25.18	23.38	1.80	7.15
Butyl butanoate	14.99	16.24	-1.25	-8.34
Butyl glycolate	19.15	22.07	-2.92	-15.25
Butyl isocyanate	8.84	8.88	-0.04	-0.45
Butyl lactate	19.02	21.78	-2.76	-14.51
Butyl propanoate	12.82	13.56	-0.74	-5.77
Butyl t-octyl ether	18.37	18.41	-0.04	-0.22
Butyl-1,2-dinitrate	18.3	18.41	-0.11	-0.60
Butylacetamide	24.11	26.92	-2.81	-11.65
Butylbenzene	16.36	18.13	-1.77	-10.82
Butylcellosolve	16.67	18.7	-2.03	-12.18
Butylcyclohexane	15.77	16.13	-0.36	-2.28
Butylcyclopentane	13.24	11.96	1.28	9.67
Butylmethylketone	10.27	10.19	0.08	0.78
Butylsilane	2.84	2.89	-0.05	-1.76
Butyric acid methylester	7.85	9.28	-1.43	-18.22
Butyronitrile	9.08	7.22	1.86	20.48
Camphene	13.15	13.28	-0.13	-0.99
Camphor	19.31	18.98	0.33	1.71
Capraldehyde	22.12	22.41	-0.29	-1.31
Caproic aldehyde	10.48	11.68	-1.20	-11.45
Caprylene	9.42	10.14	-0.72	-7.64
Caprylic aldehyde	16.07	17.04	-0.97	-6.04
Carbitol	21.52	20.47	1.05	4.88
Carbofuran	52.22	52.03	0.19	0.36
Carvone	22.66	23.95	-1.29	-5.69
Cedrol	35.17	33.6	1.57	4.46
Cetane	32.69	32.57	0.12	0.37
Cetene	31.11	31.6	-0.49	-1.58
Chloral	6.68	9.91	-3.23	-48.35
Chlorfenvinphos	45.7	43.58	2.12	4.64
Chloroacetic acid	19.45	17.44	2.01	10.33
Chloroacetic acid ethylester	12.56	11.62	0.94	7.48

Chloroacetone	10.27	8.25	2.02	19.67
Chloroacetyl chloride	8.47	7.57	0.90	10.63
Chlorobenzene	10.31	12.25	-1.94	-18.82
Chlorocyclohexane	11.27	11.16	0.11	0.98
Chloroethane	-0.42	1.12	-1.54	366.67
Chloroethylene	-3.03	-1.28	-1.75	57.76
Chloropentafluorobenzene	9.31	9.39	-0.08	-0.86
Chloroprene	3.02	3.06	-0.04	-1.32
Chloropropham	44.99	42.84	2.15	4.78
Chlorothalonil	40.51	39.87	0.64	1.58
Chlorpyrifos-methyl	41.43	41.47	-0.04	-0.10
Chlorpyriphos	43.4	44.67	-1.27	-2.93
Cinnamaldehyde	23.34	20.24	3.10	13.28
cis 3-Hexenyl butyrate	20.52	20.47	0.05	0.24
cis- Cyclooctene	11.41	13.05	-1.64	-14.37
cis-1,1,1,4,4,4-Hexafluoro-2-butene	0.8	-1.79	2.59	323.75
cis-1,2-Dibromoethylene	9.2	8.14	1.06	11.52
cis-1,2-Dichloroethylene	3.26	3.23	0.03	0.92
cis-1,2-Dimethylcyclohexane	9.82	9.28	0.54	5.50
cis-1,2-Dimethylcyclopentane	6.85	5.11	1.74	25.40
cis-1,3-Dichloropropene	7.42	5.85	1.57	21.16
cis-1,3-Dimethylcyclohexane	8.84	9.28	-0.44	-4.98
cis-1,3-Dimethylcyclopentane	5.36	5.11	0.25	4.66
cis-1,3-Pentadiene	1.72	0.95	0.77	44.77
cis-1,4-Dichloro-2-butene	12.96	11.45	1.51	11.65
cis-1,4-Dimethylcyclohexane	9.29	9.28	0.01	0.11
cis-1,4-Hexadiene	3.65	3.63	0.02	0.55
cis-1-Phenyl-1-propene	15.41	14.3	1.11	7.20
cis-2-Butene	-1.86	-0.77	-1.09	58.60
cis-2-Heptene	6.83	7.28	-0.45	-6.59
cis-2-Hexene	4.03	4.6	-0.57	-14.14
cis-2-Methylcyclohexanol	15.47	16.3	-0.83	-5.37
cis-2-Octene	9.27	9.96	-0.69	-7.44
cis-2-Pentene	1.06	1.92	-0.86	-81.13
cis-3-Heptene	6.51	7.28	-0.77	-11.83
cis-3-Hexene	4.05	4.6	-0.55	-13.58
cis-3-Hexenyl 2-methylbutyrate	21.81	21.67	0.14	0.64
cis-3-Hexenyl propionate	18.18	17.78	0.40	2.20
cis-3-Octene	9.36	9.96	-0.60	-6.41
cis-4,6-Dimethyl-1,3-dioxane	12.01	11.51	0.50	4.16
cis-4-Octene	9.27	9.96	-0.69	-7.44
cis-5-Octenyl propionate	23.75	23.15	0.60	2.53

cis-Crotononitrile	7.86	6.08	1.78	22.65
cis-Cyclohexane-1,2-dinitrate	24.37	25.49	-1.12	-4.60
cis-Cyclohexane-1,3-dinitrate	27.15	25.49	1.66	6.11
cis-Decalin	16.95	14.87	2.08	12.27
cis-Stilbene	28.71	29.37	-0.66	-2.30
cis-Verbenol	24.45	22.81	1.64	6.71
Citronellal	20.43	18.64	1.79	8.76
Citronellyl acetate	25.14	23.21	1.93	7.68
Coumarin	30.83	33.08	-2.25	-7.30
Crotonaldehyde	7.36	5.17	2.19	29.76
Cryofluorane	-1.71	-1.68	-0.03	1.75
Cumene	12.76	13.96	-1.20	-9.40
Cyanazine	58.73	60.94	-2.21	-3.76
Cyanoethylene	4.56	3.57	0.99	21.71
Cyclobutane	-1.08	1.52	-2.60	240.74
Cyclobutanone	7.19	10.14	-2.95	-41.03
Cyclogeraniolane	10.48	11.11	-0.63	-6.01
Cycloheptane	8.8	7.62	1.18	13.41
Cycloheptene	9.01	12.3	-3.29	-36.51
Cyclohexane	5.11	6.88	-1.77	-34.64
Cyclohexanethiol	12.9	12.82	0.08	0.62
Cyclohexanol	16.89	15.1	1.79	10.60
Cyclohexanone	12.82	15.22	-2.40	-18.72
Cyclohexyl azide	14.19	14.87	-0.68	-4.79
Cyclohexylamine	10.62	12.02	-1.40	-13.18
Cyclohexylbenzene	24.37	23.15	1.22	5.01
Cyclooctane	12.16	8.37	3.79	31.17
Cyclopentane	2.2	2.72	-0.52	-23.64
Cyclopentanol	14.5	10.93	3.57	24.62
Cyclopentanone	10.33	13.1	-2.77	-26.82
Cymperator	66.94	67.5	-0.56	-0.84
DBCP	17.84	17.9	-0.06	-0.34
DDD	45.77	45.07	0.70	1.53
DDE	43.37	42.5	0.87	2.01
DDT	47.07	45.3	1.77	3.76
Decabromodiphenyl ether	77.26	80.29	-3.03	-3.92
Decachlorodiphenyl ether	61.68	63.73	-2.05	-3.32
Decafluorotetrahydrothiophene	5.84	5.34	0.50	8.56
Decamethylcyclopentasiloxane	21.03	21.27	-0.24	-1.14
Decamethyltetrasiloxane	18.87	20.64	-1.77	-9.38
Decane	15.81	16.47	-0.66	-4.17
Decanedioic acid	59.5	59.17	0.33	0.55

Decyl-1,2-dinitrate	34.05	34.51	-0.46	-1.35
Decylbenzene	32.88	34.22	-1.34	-4.08
Decylcyclohexane	32.71	32.23	0.48	1.47
delta-HCH	33.28	32.57	0.71	2.13
Demeton	33.57	32.51	1.06	3.16
Deprenyl	26.3	24.92	1.38	5.25
Devrinol	47.27	49.81	-2.54	-5.37
Diallate	38.27	37.65	0.62	1.62
Diamylamine	19.53	21.32	-1.79	-9.17
Dibenz[ah]anthracene	66.25	65.67	0.58	0.88
Dibenzo[b,d]thiophene	33.25	33.48	-0.23	-0.69
Dibenzo[b,e]pyridine	41.06	40.5	0.56	1.36
Dibenzo-p-dioxin	35.76	39.65	-3.89	-10.88
Dibenzylsulfide	36.07	37.25	-1.18	-3.27
Dibutyl carbonate	20.55	20.7	-0.15	-0.73
Dibutyl decanedioate	46.8	48.21	-1.41	-3.01
Dibutyl maleate	34.65	30.97	3.68	10.62
Dibutyl oxalate	27.09	26.75	0.34	1.26
Dibutyl phthalate	42.08	43.3	-1.22	-2.90
Dibutylacetamide	26.43	27.2	-0.77	-2.91
Dibutylamine	14.14	15.96	-1.82	-12.87
Dibutylsulfide	15.98	17.84	-1.86	-11.64
Dibutylsulfoxide	26.51	29.43	-2.92	-11.01
Dichlone	48.44	46.72	1.72	3.55
Dichloroacetic acid	20.72	20.07	0.65	3.14
Diclofop-methyl	52.71	48.89	3.82	7.25
Dicofol	52.96	53.46	-0.50	-0.94
Diethion	50.26	50.49	-0.23	-0.46
Diethyl disulfide	12.85	12.59	0.26	2.02
Diethyl glutarate	25.45	24.06	1.39	5.46
Diethyl malonate	19.63	18.7	0.93	4.74
Diethyl methylphosphonate	18.6	18.41	0.19	1.02
Diethyl oxalate	18.55	16.02	2.53	13.64
Diethyl phthalate	32.53	32.57	-0.04	-0.12
Diethyl succinate	22.8	21.38	1.42	6.23
Diethyl sulphide	6.37	7.11	-0.74	-11.62
Diethylacetamide	17.74	16.47	1.27	7.16
Diethylacetic acid	20.6	20.58	0.02	0.10
Diethylamine	2.94	5.23	-2.29	-77.89
Diethylcarbonate	10.41	9.96	0.45	4.32
Diethyleneglycol monobutyl ether	25.86	25.83	0.03	0.12
Diethylketone	7.45	7.51	-0.06	-0.81

Diethylsulfoxide	21.12	18.7	2.42	11.46
Diheptylamine	30.24	32.05	-1.81	-5.99
Dihexyl phthalate	51.09	54.03	-2.94	-5.75
Dihexyl sulfide	28.48	28.57	-0.09	-0.32
Dihexylacetamide	36.53	37.93	-1.40	-3.83
Dihexyladipate	48	48.21	-0.21	-0.44
Dihexylamine	24.89	26.69	-1.80	-7.23
Dihydropinene	13.87	10.25	3.62	26.10
Diisobutyl ether	9.52	9.91	-0.39	-4.10
Diisobutyl phthalate	39.99	40.33	-0.34	-0.85
Diisobutylamine	11.53	12.76	-1.23	-10.67
Diisobutylene	6.96	6.65	0.31	4.45
Diisopropyl ether	4.05	4.77	-0.72	-17.78
Diisopropyl methylphosphonate	19.13	21.04	-1.91	-9.98
Diisopropyl sulfide	9.13	8.94	0.19	2.08
Diisopropylamine	5.63	7.05	-1.42	-25.22
Diisopropylsulfoxide	21.35	20.52	0.83	3.89
Dimethoate	45.35	45.53	-0.18	-0.40
Dimethyl carbonate	6.57	6.77	-0.20	-3.04
Dimethyl disulfide	8.05	7	1.05	13.04
Dimethyl hexanedioate	25.07	23.55	1.52	6.06
Dimethyl oxalate	15.3	12.82	2.48	16.21
Dimethyl phthalate	30.76	29.37	1.39	4.52
Dimethyl sulfide	1.06	1.52	-0.46	-43.40
Dimethylacetal	3.59	2.77	0.82	22.84
Dimethylacetamide	14.83	11.79	3.04	20.50
Dimethylamine	-1.68	0.55	-2.23	132.74
Dimethylether	-4.36	-1.05	-3.31	75.92
Di-n-butylether	11.93	12.88	-0.95	-7.96
Di-n-propyl phthalate	38.6	37.93	0.67	1.74
Dioctyl sulfide	37.27	39.3	-2.03	-5.45
Dioctylamine	35.55	37.42	-1.87	-5.26
Dipentyl phthalate	47.1	48.66	-1.56	-3.31
Diphenyl ether	26.14	27.2	-1.06	-4.06
Diphenylmethane	26.94	27.83	-0.89	-3.30
Dipropyl carbonate	15.31	15.33	-0.02	-0.13
Dipropyl ether	6.2	7.51	-1.31	-21.13
Dipropyl malonate	24.21	24.06	0.15	0.62
Dipropyl oxalate	22.05	21.38	0.67	3.04
Dipropyl sulfide	11.74	12.48	-0.74	-6.30
Dipropylamine	9.05	10.59	-1.54	-17.02
Dipropylsulfoxide	23.53	24.06	-0.53	-2.25

Di-s-butylsulfide	14.21	14.3	-0.09	-0.63
Disulfoton	37.82	36.62	1.20	3.17
Diuron	49.13	50.15	-1.02	-2.08
Docosane	49.52	48.66	0.86	1.74
Dodecafluorotetrahydrothiophene	3.45	5.97	-2.52	-73.04
Dodecahydrosqualene	61.82	61.22	0.60	0.97
Dodecamethylcyclohexasiloxane	25.85	25.03	0.82	3.17
Dodecane	21.7	21.84	-0.14	-0.65
Dodecyl acetate	31.45	32.34	-0.89	-2.83
Dopentacontane	129.64	129.15	0.49	0.38
Dotetracontane	103.87	102.32	1.55	1.49
Dotriacontane	76.75	75.49	1.26	1.64
d-Verbenone	21.79	21.67	0.12	0.55
Eicosane	43.87	43.3	0.57	1.30
Endrin	41.61	40.16	1.45	3.48
Enilconazole	50.24	50.95	-0.71	-1.41
Epichlorohydrin	9.28	7.74	1.54	16.59
Ethalfluralin	49.43	48.27	1.16	2.35
Ethanediol diacetate	20.15	16.02	4.13	20.50
Ethanethiol	2.43	2.72	-0.29	-11.93
Ethanol	6.32	6.2	0.12	1.90
Ethene	-7.95	-5.79	-2.16	27.17
Ethyl 2-methylbutanoate	8.33	12.08	-3.75	-45.02
Ethyl 2-methylpentanoate	13.66	14.76	-1.10	-8.05
Ethyl acetate	5.23	5.51	-0.28	-5.35
Ethyl acetoacetate	17.29	15.33	1.96	11.34
Ethyl acrylate	7.3	7.22	0.08	1.10
Ethyl benzoate	20.76	20.58	0.18	0.87
Ethyl butanoate	10.14	10.88	-0.74	-7.30
Ethyl decanoate	26.4	26.97	-0.57	-2.16
Ethyl ether	0.89	2.14	-1.25	-140.45
Ethyl formate	2.74	2.72	0.02	0.73
Ethyl glycolate	15.18	16.7	-1.52	-10.01
Ethyl hexanoate	16.04	16.24	-0.20	-1.25
Ethyl iodide	5.51	5.34	0.17	3.09
Ethyl isobutanoate	8.43	9.39	-0.96	-11.39
Ethyl isovalerate	11.13	12.08	-0.95	-8.54
Ethyl lactate	14.66	16.41	-1.75	-11.94
Ethyl levulinate	20.56	18.01	2.55	12.40
Ethyl perfluorooctanoate	16.52	17.67	-1.15	-6.96
Ethyl propanoate	7.62	8.2	-0.58	-7.61
Ethyl t-amyl ether	7.07	7.34	-0.27	-3.82

Ethyl t-butyl ether	4.54	4.66	-0.12	-2.64
Ethyl t-octyl ether	13.11	13.05	0.06	0.46
Ethyl trans-cinnamate	27.57	24.81	2.76	10.01
Ethylacetamide	22.88	21.55	1.33	5.81
Ethylamine	0.32	1.92	-1.60	-500.00
Ethylbenzene	10.88	12.76	-1.88	-17.28
Ethylcarbamate	21.1	20.81	0.29	1.37
Ethylcyclohexane	10.14	10.76	-0.62	-6.11
Ethylcyclopentane	7.3	9.34	-2.04	-27.95
Ethylenedichloride	5.63	7.22	-1.59	-28.24
Ethylepoxide	3.48	4.31	-0.83	-23.85
Ethylisopropylketone	9.28	8.71	0.57	6.14
Ethylmethylether	-1.68	0.55	-2.23	132.74
Ethylnitrate	6.11	5.68	0.43	7.04
Ethylnitrite	-0.72	-0.31	-0.41	56.94
Ethyl-n-propylether	3.54	4.83	-1.29	-36.44
Ethyl-t-butylsulfide	9.08	9.45	-0.37	-4.07
Ethyne	-9.05	-7.5	-1.55	17.13
Ethynylbenzene	11.27	11.62	-0.35	-3.11
Fenchlorphos	40.84	40.22	0.62	1.52
Fenchone	17.12	18.7	-1.58	-9.23
Fenitrothion	42.84	42.39	0.45	1.05
Fenoprop	43.63	41.36	2.27	5.20
Fenoxycarb	61.51	63.28	-1.77	-2.88
Fenpropidin	38.82	39.7	-0.88	-2.27
Fluorene	35.32	37.53	-2.21	-6.26
Fluorobenzene	5.68	8.02	-2.34	-41.20
Fluoroethane	-5.31	-3.91	-1.40	26.37
Fluoroethylene	-8.16	-5.9	-2.26	27.70
Formic acid	7.42	8.54	-1.12	-15.09
Freon 113	2.04	2.14	-0.10	-4.90
Furfural	14.38	12.7	1.68	11.68
Furfuranol	17.24	17.96	-0.72	-4.18
gamma-Bisabolene	29.39	32.74	-3.35	-11.40
Glycerol formal	19.87	18.58	1.29	6.49
Glyceryltriacetate	31.22	27.83	3.39	10.86
Guajen	27.03	25.83	1.20	4.44
Hemimellitene	15.12	13.05	2.07	13.69
Heneicosane	46.7	45.98	0.72	1.54
Heptachlor epoxide	48.32	49.24	-0.92	-1.90
Heptacosane	62.54	62.08	0.46	0.74
Heptadecane	35.44	35.25	0.19	0.54

Heptafluoropropyl trifluoromethyl ether	-1.65	-1.91	0.26	-15.76
Heptane	7.13	8.42	-1.29	-18.09
Heptanedioic acid	51.1	51.12	-0.02	-0.04
Heptanonitrile	15.7	15.27	0.43	2.74
Heptatriacontane	90.3	88.91	1.39	1.54
Heptyl bromide	15.92	16.07	-0.15	-0.94
Heptyl butyrate	23.48	24.29	-0.81	-3.45
Heptylbenzene	24.37	26.18	-1.81	-7.43
Hexachlorobenzene	31.54	30.51	1.03	3.27
Hexachlorobutadiene	20.09	21.27	-1.18	-5.87
Hexacosane	60.25	59.4	0.85	1.41
Hexadecylamine	39.75	39.47	0.28	0.70
Hexafluorobenzene	5.36	5.17	0.19	3.54
Hexamethyldisiloxane	7.14	5.34	1.80	25.21
Hexamethyleneglycol	35.22	32.34	2.88	8.18
Hexane	4.03	5.74	-1.71	-42.43
Hexane-1,5-dinitrate	24.46	25.72	-1.26	-5.15
Hexane-2,5-dinitrate	23.8	24.35	-0.55	-2.31
Hexanenitrile	13.75	12.59	1.16	8.44
Hexapentacontane	139.08	139.88	-0.80	-0.58
Hexatetracontane	114.52	113.05	1.47	1.28
Hexatriacontane	87.71	86.22	1.49	1.70
Hexyl bromide	13.07	13.39	-0.32	-2.45
Hexyl glycolate	24.79	27.43	-2.64	-10.65
Hexyl hexanoate	25.74	26.97	-1.23	-4.78
Hexyl t-octyl ether	23.79	23.78	0.01	0.04
Hexyl-1,2-dinitrate	23.35	23.78	-0.43	-1.84
Hexylbenzene	21.92	23.49	-1.57	-7.16
Hydrogenperoxide	14.44	14.87	-0.43	-2.98
Icosafluorononane	10.82	8.77	2.05	18.95
Indeno[1,2,3-cd]pyrene	74.16	76.29	-2.13	-2.87
Iodobenzene	16.36	16.19	0.17	1.04
Isoamyl acetate	12.16	12.08	0.08	0.66
Isoamyl isobutyrate	15.7	15.96	-0.26	-1.66
Isobutyl acetate	9.3	9.39	-0.09	-0.97
Isobutyl acrylate	11.19	11.11	0.08	0.71
Isobutyl butanoate	13.18	14.76	-1.58	-11.99
Isobutyl chloride	4.17	5	-0.83	-19.90
Isobutyl formate	7.3	6.6	0.70	9.59
Isobutyl isobutyrate	12.9	13.28	-0.38	-2.95
Isobutyl t-butylether	8.58	8.54	0.04	0.47
Isobutylbenzene	14.9	16.64	-1.74	-11.68

Isobutylene	-2.68	-1.74	-0.94	35.07
Isobutylmethylether	3.23	4.43	-1.20	-37.15
iso-Butylsilane	2.13	1.4	0.73	34.27
Isobutyronitrile	7.7	5.74	1.96	25.45
Isocrotonic acid	17.69	15.56	2.13	12.04
Isocumene	13.45	15.44	-1.99	-14.80
Isooctane	6.78	7.28	-0.50	-7.37
Isopentane	0.4	1.57	-1.17	-292.50
Isopentene	0.57	0.95	-0.38	-66.67
Isopentyl alcohol	14.33	12.76	1.57	10.96
Isopentyl bromide	7.68	9.22	-1.54	-20.05
Isopentyl formate	9.74	9.28	0.46	4.72
Isophytol	45.69	44.56	1.13	2.47
Isoprene	0.74	-0.02	0.76	102.70
Isopropyl formate	4.26	4.03	0.23	5.40
Isopropyl lactate	14.99	17.73	-2.74	-18.28
Isopropyl methyl ether	0.6	1.86	-1.26	-210.00
iso-Propyl nitrite	1.27	1	0.27	21.26
Isopropyl propyl ether	5.42	6.14	-0.72	-13.28
Isopropylacetate	6.31	6.83	-0.52	-8.24
Isopropylamine	0.72	2.83	-2.11	-293.06
Isopropylcyclohexane	12.61	11.96	0.65	5.15
Isopropylcyclopentane	9.55	7.8	1.75	18.32
Isopulegol	20.88	20.75	0.13	0.62
Isoquinoline	23.86	24.41	-0.55	-2.31
Linalool	21	19.9	1.10	5.24
Linalyl acetate	22.37	20.87	1.50	6.71
Lindane	33.02	32.57	0.45	1.36
L-Menthone	19.57	21.49	-1.92	-9.81
Malathion	44.9	45.93	-1.03	-2.29
Malonic acid	40.8	40.39	0.41	1.00
m-Cresol	21.29	20.07	1.22	5.73
Menthol	23.69	21.38	2.31	9.75
Methacrolein	3.94	4.2	-0.26	-6.60
Methanethiol	-0.42	-0.08	-0.34	80.95
Methanol	4.45	4.6	-0.15	-3.37
Methoxybenzene	13.19	11.16	2.03	15.39
Methyl acetate	3.17	3.91	-0.74	-23.34
Methyl acrylate	5.37	5.63	-0.26	-4.84
Methyl arachidate	50.4	52.2	-1.80	-3.57
Methyl benzoate	18.86	18.98	-0.12	-0.64
Methyl cellosolve	10.84	11.79	-0.95	-8.76

Methyl decanoate	24.31	25.38	-1.07	-4.40
Methyl docosanoate	57.4	57.57	-0.17	-0.30
Methyl formate	0.66	1.12	-0.46	-69.70
Methyl heneicosanoate	54.24	54.89	-0.65	-1.20
Methyl heptacosanoate	70.67	70.98	-0.31	-0.44
Methyl heptadecanoate	42.98	44.16	-1.18	-2.75
Methyl hexacosanoate	67.98	68.3	-0.32	-0.47
Methyl hexadecanoate	40.3	41.47	-1.17	-2.90
Methyl hexanoate	13.22	14.65	-1.43	-10.82
Methyl hydroxyacetate	14.4	15.16	-0.76	-5.28
Methyl isoamyl ketone	12.33	11.39	0.94	7.62
Methyl isobutyl ketone	9.02	8.71	0.31	3.44
Methyl laurate	29.45	30.74	-1.29	-4.38
Methyl levulinate	19.18	16.41	2.77	14.44
Methyl methacrylate	7.42	7.17	0.25	3.37
Methyl nonadecanoate	47.77	49.52	-1.75	-3.66
Methyl nonanoate	21.57	22.69	-1.12	-5.19
Methyl octacosanoate	73.43	73.67	-0.24	-0.33
Methyl octadecanoate	46.12	46.84	-0.72	-1.56
Methyl octanoate	18.86	20.01	-1.15	-6.10
Methyl pentacosanoate	65.3	65.62	-0.32	-0.49
Methyl pentadecanoate	37.56	38.79	-1.23	-3.27
Methyl propanoate	5.51	6.6	-1.09	-19.78
Methyl propyl sulfide	6.7	7	-0.30	-4.48
Methyl t-butyl sulfide	6.79	6.65	0.14	2.06
Methyl tetracosanoate	62.47	62.93	-0.46	-0.74
Methyl tetradecanoate	35.33	36.11	-0.78	-2.21
Methyl t-pentyl ether	5.74	5.74	0.00	0.00
Methyl tridecanoate	32.25	33.42	-1.17	-3.63
Methyl undecanoate	26.94	28.06	-1.12	-4.16
Methyl valerate	10.62	11.96	-1.34	-12.62
Methylacetamide	21.73	19.21	2.52	11.60
Methylacetoacetate	14.95	13.73	1.22	8.16
Methylamine	-2.03	-0.42	-1.61	79.31
Methylbenzene	8.33	10.08	-1.75	-21.01
Methylchloroacetate	11.43	10.02	1.41	12.34
Methylcyclohexane	6.96	8.08	-1.12	-16.09
Methylcyclopentane	4.26	7.34	-3.08	-72.30
Methylenanthate	15.87	17.33	-1.46	-9.20
Methylenecyclohexane	7.3	11.33	-4.03	-55.21
Methylisocyanate	1.28	1.17	0.11	8.59
Methyllactate	13.4	14.87	-1.47	-10.97

Methylmalonic acid	45.46	41.59	3.87	8.51
Methylnitrite	-3.57	-1.91	-1.66	46.50
Methyl-n-propylketone	7.53	7.51	0.02	0.27
Methyloxiran	0.8	1.63	-0.83	-103.75
Methylparathion	41.87	40.9	0.97	2.32
Methylphosphonic acid dimethyl ester	16.89	15.22	1.67	9.89
Methylpropylether	1.23	3.23	-2.00	-162.60
Methylthio bis(trifluoromethyl)phosphine	6.37	6.54	-0.17	-2.67
Metolachlor	42.14	42.04	0.10	0.24
Molinate	29.3	29.54	-0.24	-0.82
Monocrotophos	40.16	40.22	-0.06	-0.15
Morpholine	10.75	8.14	2.61	24.28
MTBE	2.68	3.06	-0.38	-14.18
m-Toluidine	19.44	21.21	-1.77	-9.10
Mustard gas	21.86	19.33	2.53	11.57
Myristyl alcohol	38.98	38.39	0.59	1.51
N,N-Dibutylformamide	25.11	26.63	-1.52	-6.05
N,N-Diethylaniline	21.4	20.75	0.65	3.04
N,N-Diethylformamide	16.21	15.9	0.31	1.91
N,N-Dimethyl-1-naphthylamine	28.45	30.34	-1.89	-6.64
N,N-Dimethyl-2,3-dimethyl-3-phenyl-2-butanamine	27.1	24.92	2.18	8.04
N,N-Dimethyl-3-methyl-3-phenyl-2-butaneamine	23.93	24.06	-0.13	-0.54
N,N-Dimethylaniline	17.24	16.07	1.17	6.79
N,N-Dimethylbenzylamine	15.72	17.44	-1.72	-10.94
N,N-Dimethyldodecylamine	28.98	29.2	-0.22	-0.76
N,N-Dimethylethanolamine	11.85	13.5	-1.65	-13.92
N,N-Dimethylformamide	12.94	11.22	1.72	13.29
N,N-Dimethylhexadecylamine	39.91	39.93	-0.02	-0.05
N,N-Dimethyloctylamine	18.25	18.47	-0.22	-1.21
N,N-Dimethyltetradecylamine	34.53	34.57	-0.04	-0.12
N,N-Dipropylformamide	20.03	21.27	-1.24	-6.19
Naphthalene	22.7	22.86	-0.16	-0.70
N-Benzylformamide	36.3	36.05	0.25	0.69
n-Butyl acetate	10.42	10.88	-0.46	-4.41
n-Butyl acrylate	12.24	12.59	-0.35	-2.86
n-Butyl methyl ether	4.26	5.91	-1.65	-38.73
n-Butylbenzoate	24.75	25.95	-1.20	-4.85
n-Butylethylether	6.56	7.51	-0.95	-14.48
N-Butylformamide	25.68	26.35	-0.67	-2.61
n-Butylformate	8.14	8.08	0.06	0.74
n-Butylmethacrylate	14.5	14.13	0.37	2.55
n-Butylmethylsulfide	9.63	9.68	-0.05	-0.52

n-Decyl alcohol	28.25	27.66	0.59	2.09
n-Decylacetate	25.8	26.97	-1.17	-4.53
n-Decylamine	22.6	23.38	-0.78	-3.45
n-Dodecylamine	26.83	28.74	-1.91	-7.12
Neburon	58.25	57.85	0.40	0.69
Neohexane	2.17	3.4	-1.23	-56.68
N-Ethylmorpholine	12.38	9.96	2.42	19.55
n-Heptaldehyde	13.33	14.36	-1.03	-7.73
n-Heptylamine	14.55	15.33	-0.78	-5.36
n-Hexanoic acid	24.14	22.07	2.07	8.57
n-Hexylacetate	15.79	16.24	-0.45	-2.85
n-Hexylamine	12.33	12.65	-0.32	-2.60
Nitrobenzene	19.95	20.24	-0.29	-1.45
Nitrocyclohexane	19.06	18.53	0.53	2.78
Nitroethane	8.94	7.8	1.14	12.75
Nitroisobutane	11.79	11.68	0.11	0.93
N-Methyl-2,3-dimethyl-3-phenyl-2-butaneamine	26.68	25.43	1.25	4.69
N-Methyl-2-pyrrolidone	19.09	19.04	0.05	0.26
N-Methyl-3-methyl-3-phenyl-2-butaneamine	22.8	24.58	-1.78	-7.81
N-Methylformamide	19.75	18.64	1.11	5.62
N-Methylmorpholine	8.71	7.62	1.09	12.51
N-Methylpiperidine	9.11	8.54	0.57	6.26
N-Methylpropionamide	19.63	21.89	-2.26	-11.51
N-Methylpyrrolidine	5	4.37	0.63	12.60
N-Methylsuccinimide	26.75	26.69	0.06	0.22
N-Nitrosodiethylamine	16.03	18.01	-1.98	-12.35
N-Nitrosodimethylamine	13.18	13.33	-0.15	-1.14
N-Nitrosopiperidine	21.32	21.84	-0.52	-2.44
N-Nitrosopyrrolidine	22.44	19.73	2.71	12.08
n-Nonanal	17.58	19.73	-2.15	-12.23
n-Nonylamine	19.52	20.7	-1.18	-6.05
n-Nonylmercaptan	20.14	21.49	-1.35	-6.70
n-Octanoic acid	30.51	27.43	3.08	10.10
n-Octylacetate	20.66	21.61	-0.95	-4.60
n-Octylamine	17.77	18.01	-0.24	-1.35
Nonacosane	68.48	67.44	1.04	1.52
Nonadecane	41.04	40.62	0.42	1.02
Nonafluorocyclopentane	1.29	1.17	0.12	9.30
Nonane	12.8	13.79	-0.99	-7.73
Nonanedinitrile	24.51	27.49	-2.98	-12.16
Nonanedioic acid	57.5	56.48	1.02	1.77
Nonylbenzene	30.02	31.54	-1.52	-5.06

Nopinene	13.7	13.5	0.20	1.46
n-Propyl acetate	7.74	8.2	-0.46	-5.94
n-Propyl benzoate	22.51	23.26	-0.75	-3.33
n-Propyl iodide	7.12	8.02	-0.90	-12.64
n-Propylformate	5.48	5.4	0.08	1.46
n-Propylnitrate	8.62	8.37	0.25	2.90
n-Undecylamine	25.94	26.06	-0.12	-0.46
n-Valeraldehyde	7.65	8.99	-1.34	-17.52
O,O-Diethyl O-2-diethylaminoethyl phosphate	31.93	32.45	-0.52	-1.63
O,O-Diethyl O-2-diethylaminoethyl thiophosphate	31.75	32.45	-0.70	-2.20
O,O-Diethyl O-2-dimethylaminoethyl thiophosphate	28.6	27.77	0.83	2.90
O,O-Diethyl O-3-(1-dimethylamino)prop-2-yl thiophosphate	29.69	29.09	0.60	2.02
O,O-Diethyl O-3-diethylaminopropyl thiophosphate	34.28	35.14	-0.86	-2.51
O,O-Diethyl S-2-diethylaminoethyl dithiophosphate	36.26	36.56	-0.30	-0.83
o-Cresol	19.5	20.07	-0.57	-2.92
Octachlorodibenzofuran	66.16	66.13	0.03	0.05
Octachlorodibenzo-p-dioxin	69.56	68.87	0.69	0.99
Octachloronaphthalene	52.84	52.09	0.75	1.42
Octacosane	65.66	64.76	0.90	1.37
Octadecane	38.26	37.93	0.33	0.86
Octamethylcyclotetrasiloxane	16.68	16.13	0.55	3.30
Octamethyltrisiloxane	13.07	12.99	0.08	0.61
Octane	9.99	11.11	-1.12	-11.21
Octanedioic acid	55.6	53.8	1.80	3.24
Octatetracontane	119.73	118.42	1.31	1.09
Octatriacontane	93.01	91.59	1.42	1.53
Octyl glycolate	29.82	32.8	-2.98	-9.99
Octyl-1,2-dinitrate	28.81	29.14	-0.33	-1.15
Octylbenzene	27.23	28.86	-1.63	-5.99
o-Cumenol	21.69	23.95	-2.26	-10.42
o-Toluidine	19.84	21.21	-1.37	-6.91
Oxetane	2.05	0.6	1.45	70.73
Oxirane	-1.34	0.32	-1.66	123.88
Parathion	44.98	44.1	0.88	1.96
PCNB	39.04	38.5	0.54	1.38
p-Cresol	21.52	20.07	1.45	6.74
p-Cymene	15.27	15.44	-0.17	-1.11
Pelargonic acid	33.1	30.11	2.99	9.03
Pentachlorobenzene	27.23	26.86	0.37	1.36
Pentachloroethane	13.36	12.7	0.66	4.94

Pentachlorophenol	39.04	36.85	2.19	5.61
Pentacontane	124.69	123.78	0.91	0.73
Pentacosane	57.61	56.71	0.90	1.56
Pentadecane	29.97	29.89	0.08	0.27
Pentadecylamine	36.82	36.79	0.03	0.08
Pentaethylbenzene	30.54	29.43	1.11	3.63
Pentafluoro(fluorosulfonato)sulfur	1.35	1.46	-0.11	-8.15
Pentafluorobenzene	6	5.74	0.26	4.33
Pentafluoroethane	-6.34	-4.25	-2.09	32.97
Pentafluorophenol	14.45	15.73	-1.28	-8.86
Pentane	1.2	3.06	-1.86	-155.00
Pentane-1,4-dinitrate	23.03	23.04	-0.01	-0.04
Pentanedinitrile	15.84	16.76	-0.92	-5.81
Pentanedioic acid	45.2	45.75	-0.55	-1.22
Pentanenitrile	11.52	9.91	1.61	13.98
Pentanoic acid	19.86	19.38	0.48	2.42
Pentatriacontane	85.05	83.54	1.51	1.78
Pentyl butyrate	16.95	18.93	-1.98	-11.68
Pentyl glycolate	21.95	24.75	-2.80	-12.76
Pentyl t-octyl ether	21.12	21.1	0.02	0.09
Pentyl-1,2-dinitrate	20.72	21.1	-0.38	-1.83
Pentylcyclopentane	16.12	14.65	1.47	9.12
Perchlorobiphenyl	62	61.68	0.32	0.52
Perfluoro-1,4-dithiane	4.35	4.71	-0.36	-8.28
Perfluoro-1,4-dithiane S,S'-bis(tetrafluoride)	10.86	8.82	2.04	18.78
Perfluoro-2-(fluorosulfato)butane	4.35	4.31	0.04	0.92
Perfluoro-2,3-dimethylbutane	3.29	2.2	1.09	33.13
Perfluoro-2-methylbutane	0.39	0.2	0.19	48.72
Perfluoro-2-methylpentane	3.01	2.32	0.69	22.92
Perfluoro-3-methylpentane	3.01	2.32	0.69	22.92
Perfluorobutane	-2.46	-1.79	-0.67	27.24
Perfluorobutylhexane	13.49	14.25	-0.76	-5.63
Perfluorobutyloctane	18.5	19.61	-1.11	-6.00
Perfluorobutylpentane	11.05	11.56	-0.51	-4.62
Perfluorocyclobutane	-2.8	-0.77	-2.03	72.50
Perfluorocyclohexane	3.09	3.46	-0.37	-11.97
Perfluorocyclopentane	-0.23	-0.14	-0.09	39.13
Perfluoro-dibutylmethylamine	11.88	11.62	0.26	2.19
Perfluorodiethylsulfur disulfide	4.18	4.77	-0.59	-14.11
Perfluorodiglyme	4.13	3.74	0.39	9.44
Perfluorodimethylsulfur difluoride	-0.37	-0.48	0.11	-29.73
Perfluoroethane	-8.51	-6.02	-2.49	29.26

Perfluoroethylcyclohexane	7.46	7.57	-0.11	-1.47
Perfluoroethylmethylsulfide	-1.6	-1.68	0.08	-5.00
Perfluoroethylmethylsulfur difluoride	1.75	2.14	-0.39	-22.29
Perfluorohexane	3.08	2.43	0.65	21.10
Perfluorohexanoic acid	18.05	19.27	-1.22	-6.76
Perfluorohexylhexane	17.96	18.47	-0.51	-2.84
Perfluoromethylcyclohexane	4.89	5.46	-0.57	-11.66
Perfluoromethylpropylsulfide	1.19	0.43	0.76	63.87
Perfluoromethylpropylsulfur difluoride	4.24	4.26	-0.02	-0.47
Perfluorooctylethylene	13.28	10.99	2.29	17.24
Perfluoropentane	0.39	0.32	0.07	17.95
Perfluoropropane	-5.37	-3.91	-1.46	27.19
Perfluoropropene	-4.9	-6.19	1.29	-26.33
Perfluorotetrahydrofuran	-2.3	-0.02	-2.28	99.13
Perfluorotetrahydrothiophene	1.24	1.17	0.07	5.65
Perfluorotoluene	8.09	6.71	1.38	17.06
Perfluoro-trans-1,2-dimethylcyclobutane	1.77	3.23	-1.46	-82.49
Perfluoro-trans-decalin	11.68	11.79	-0.11	-0.94
Perfluorotributylamine	18.12	17.5	0.62	3.42
Perfluorotriethylamine	4.39	4.83	-0.44	-10.02
Perfluorotriglyme	8.61	9.51	-0.90	-10.45
Perfluorotripropylamine	11.17	11.16	0.01	0.09
Permethrin	60.02	59.34	0.68	1.13
Perylene	64.28	63.62	0.66	1.03
Phenanthrene	37.65	37.13	0.52	1.38
Phencyclidine	37.44	36.91	0.53	1.42
Phenethyl acetate	22.19	23.26	-1.07	-4.82
Phenethyl alcohol	22.43	23.95	-1.52	-6.78
Phenethyl butyrate	26.98	28.63	-1.65	-6.12
Phenethyl hexanoate	32.21	34	-1.79	-5.56
Phenethyl propionate	24.69	25.95	-1.26	-5.10
Phenol	19.06	18.58	0.48	2.52
Phenthoate	48.3	47.81	0.49	1.01
Phenylacetic acid	30.25	29.09	1.16	3.83
Phenylisocyanate	14.04	13.96	0.08	0.57
Phlorol	21	22.75	-1.75	-8.33
Phorate	32.01	33.08	-1.07	-3.34
Phosmet	55.4	55.34	0.06	0.11
Phytane	38.52	37.36	1.16	3.01
Phytol	49.04	47.75	1.29	2.63
Pimelonitrile	21.23	22.12	-0.89	-4.19
Pinacolone	7.82	7.85	-0.03	-0.38

Piperidine	7.99	9.05	-1.06	-13.27
p-Menthadiene	14.65	17.56	-2.91	-19.86
Pristane	35.7	34.68	1.02	2.86
Prometryn	50.09	49.69	0.40	0.80
Propanal	2.16	3.63	-1.47	-68.06
Propane	-5.5	-2.31	-3.19	58.00
Propane-1,3-dithiol	13.12	13.1	0.02	0.15
Propanenitrile	6.88	4.54	2.34	34.01
Propanil	41.55	42.5	-0.95	-2.29
Propanoic acid	13.33	14.02	-0.69	-5.18
Propanolamine	15.41	15.79	-0.38	-2.47
Propanone	2.85	2.14	0.71	24.91
Propazine	55.73	54.14	1.59	2.85
Propenal	2.45	2.66	-0.21	-8.57
Propoxur	44.4	44.5	-0.10	-0.23
Propyl 2-methylbutanoate	13.41	14.76	-1.35	-10.07
Propyl butyrate	12.61	13.56	-0.95	-7.53
Propyl glycolate	17.31	19.38	-2.07	-11.96
Propyl lactate	16.72	19.1	-2.38	-14.23
Propyl nitrite	2.14	2.37	-0.23	-10.75
Propyl propanoate	9.96	10.88	-0.92	-9.24
Propyl tert-amyl ether	9.75	10.02	-0.27	-2.77
Propyl t-octyl ether	15.2	15.73	-0.53	-3.49
Propyl-1,2-dinitrate	16.15	15.73	0.42	2.60
Propylacetamide	25.25	24.23	1.02	4.04
Propylcyclohexane	12.96	13.45	-0.49	-3.78
Propylcyclopentane	10.25	12.02	-1.77	-17.27
Propylene	-4.37	-3.28	-1.09	24.94
Propylenediamine	10.5	9.74	0.76	7.24
Propylsilane	-0.33	0.2	-0.53	160.61
Propyne	-2.85	-3.45	0.60	-21.05
p-Toluidine	19.81	21.21	-1.40	-7.07
Pulegone	21.51	22.98	-1.47	-6.83
Pyrene	47.85	49.35	-1.50	-3.13
Pyridine	8.94	10.14	-1.20	-13.42
Pyrrolidine	6.16	4.88	1.28	20.78
Pyruvaldehyde	8.31	8.08	0.23	2.77
Quinoline	23.34	25.32	-1.98	-8.48
R-1234yf	-4.73	-4.48	-0.25	5.29
R134a	-4.67	-4.42	-0.25	5.35
Raspberry ketone	31.12	32.57	-1.45	-4.66
Refrigerant 115	-5.44	-3.85	-1.59	29.23

s-Butylacetate	9.45	9.51	-0.06	-0.63
s-Butylamine	3.63	5.51	-1.88	-51.79
s-Butylbenzene	15.16	16.64	-1.48	-9.76
s-Butylnitrite	4.18	3.69	0.49	11.72
Sebaconitrile	26.48	30.17	-3.69	-13.94
sec-Hexyl acetate	13.05	13.39	-0.34	-2.61
s-Phenethyl alcohol	20.72	20.98	-0.26	-1.25
s-trans-1,3-Butadiene	-2.53	-1.57	-0.96	37.94
Styrene	11.76	11.79	-0.03	-0.26
Styrene oxide	19.43	16.7	2.73	14.05
Suberonitrile	22.68	24.81	-2.13	-9.39
Sulcatone	15.93	13.28	2.65	16.64
t-Butyl lactate	15.77	18.93	-3.16	-20.04
t-Butylacetamide	24.14	23.32	0.82	3.40
t-Butylamine	1.8	3.69	-1.89	-105.00
t-Butylbenzene	14.53	15.79	-1.26	-8.67
t-Butylhydroperoxide	12.27	11.91	0.36	2.93
t-Butylmercaptan	3.55	5.06	-1.51	-42.54
t-Butylnitrite	3.54	2.2	1.34	37.85
t-Butylthio bis(trifluoromethyl)phosphine	11.71	11.68	0.03	0.26
Terbufos	36.91	35.42	1.49	4.04
Terbutryn	49.45	49.64	-0.19	-0.38
Tetrachloroethylene	9.19	7.8	1.39	15.13
Tetracontane	98.56	96.95	1.61	1.63
Tetracosane	54.97	54.03	0.94	1.71
Tetradecane	27.92	27.2	0.72	2.58
Tetradecylbenzene	43.98	44.95	-0.97	-2.21
Tetraethylmethane	11.41	11.45	-0.04	-0.35
Tetrafluoroethene	-8.68	-9.21	0.53	-6.11
Tetrahydrofuran	3.86	5.23	-1.37	-35.49
Tetrahydropyran	5.88	5.97	-0.09	-1.53
Tetramethylene glycol	27.68	26.97	0.71	2.57
Tetramethylethene	4.47	2.32	2.15	48.10
Tetrapentacontane	134.36	134.51	-0.15	-0.11
Tetratetracontane	109.17	107.69	1.48	1.36
Thianaphthene	20.28	19.9	0.38	1.87
Thietane	6.63	6.54	0.09	1.36
Thiobencarb	44.27	43.7	0.57	1.29
Thiolane	9.13	7.45	1.68	18.40
Thiophene	5.63	6.31	-0.68	-12.08
TNT	44.34	45.01	-0.67	-1.51
Tolclofos-methyl	35.66	38.05	-2.39	-6.70

trans,trans-2,4-Hexadienyl acetate	17.45	13.96	3.49	20.00
trans-1,2-Dibromocycloheptane	20.96	20.52	0.44	2.10
trans-1,2-Dibromocyclohexane	20.04	19.78	0.26	1.30
trans-1,2-Dibromocyclooctane	22.62	21.27	1.35	5.97
trans-1,2-Dibromocyclopentane	16.28	15.62	0.66	4.05
trans-1,2-Dibromoethene	7.98	8.14	-0.16	-2.01
trans-1,2-Dichloroethylene	3.34	3.23	0.11	3.29
trans-1,2-Dimethylcyclohexane	9.82	9.28	0.54	5.50
trans-1,2-Dimethylcyclopentane	6.13	5.11	1.02	16.64
trans-1,3,5-Hexatriene	5.31	2.66	2.65	49.91
trans-1,3-Dichloropropene	7.65	7.34	0.31	4.05
trans-1,3-Dimethylcyclohexane	9.36	9.28	0.08	0.85
trans-1,3-Dimethylcyclopentane	6.12	5.11	1.01	16.50
trans-1,3-Pentadiene	1.43	0.95	0.48	33.57
trans-1,4-Dichloro-2-butene	13.41	11.45	1.96	14.62
trans-1,4-Dimethylcyclohexane	8.79	9.28	-0.49	-5.57
trans-1,4-Hexadiene	3.65	3.63	0.02	0.55
trans-1-Propenylbenzene	15.64	14.3	1.34	8.57
trans-2-Butene	-2.08	-0.77	-1.31	62.98
trans-2-Decene	15.64	15.33	0.31	1.98
trans-2-Dodecene	21.69	20.7	0.99	4.56
trans-2-Heptene	6.6	7.28	-0.68	-10.30
trans-2-Hexene	3.94	4.6	-0.66	-16.75
trans-2-Methoxy-4-(1-propenyI)phenol	29.85	26.86	2.99	10.02
trans-2-Methylcyclohexanol	15.92	16.3	-0.38	-2.39
trans-2-Octene	9.31	9.96	-0.65	-6.98
trans-2-Pentene	0.95	1.92	-0.97	-102.11
trans-2-Phenylcyclopropylamine	21.76	22.64	-0.88	-4.04
trans-3-Heptene	6.73	7.28	-0.55	-8.17
trans-3-Hexene	3.94	4.6	-0.66	-16.75
trans-3-Methylcyclohexanol	17.75	16.3	1.45	8.17
trans-3-Octene	9.38	9.96	-0.58	-6.18
trans-4,4-Dimethyl-2-pentene	4.79	4.94	-0.15	-3.13
trans-4,6-Dimethyl-1,3-dioxane	13.02	11.51	1.51	11.60
trans-4-Octene	9.25	9.96	-0.71	-7.68
trans-Crotononitrile	9.39	6.08	3.31	35.25
trans-Cycloheptane-1,2-dinitrate	28.32	26.23	2.09	7.38
trans-Cyclohexane-1,2-dinitrate	24.29	25.49	-1.20	-4.94
trans-Cyclohexane-1,3-dinitrate	24.53	25.49	-0.96	-3.91
trans-Decalin	15.91	14.87	1.04	6.54
Triacontane	71.26	70.13	1.13	1.59
Triallate	38.82	39.93	-1.11	-2.86

Tributyl borate	22.65	24.06	-1.41	-6.23
Tributyl phosphate	33.28	36.51	-3.23	-9.71
Tributylamine	21.06	23.15	-2.09	-9.92
Trichloroacetic acid	20.92	20.3	0.62	2.96
Trichloroacetylchloride	8.92	10.42	-1.50	-16.82
Trichloroethylene	5.88	5.51	0.37	6.29
Trichlorosyringol	34.93	34.68	0.25	0.72
Trichlorotoluene	19.78	19.04	0.74	3.74
Triclopyr	43.05	41.3	1.75	4.07
Tricosane	53.71	51.35	2.36	4.39
Tridecane	24.26	24.52	-0.26	-1.07
Tridecylamine	31.4	31.43	-0.03	-0.10
Tridecylbenzene	44.29	42.27	2.02	4.56
Triethanolamine	47.52	49.06	-1.54	-3.24
Triethyl borate	9.46	7.97	1.49	15.75
Triethyl phosphate	21.45	20.41	1.04	4.85
Triethylamine	6.43	7.05	-0.62	-9.64
Triisobutyl borate	18.96	19.61	-0.65	-3.43
Triisopropyl borate	11.09	11.91	-0.82	-7.39
Trimethoxyborine	4.24	3.17	1.07	25.24
Trimethyl phosphate	16.78	15.62	1.16	6.91
Trimethylamine	-1.85	0.03	-1.88	101.62
Trimethylethylene	1.2	0.77	0.43	35.83
Tripropyl borate	15.98	16.02	-0.04	-0.25
Tripropyl phosphate	28.34	28.46	-0.12	-0.42
Tripropylamine	15.44	15.1	0.34	2.20
Triptane	4.97	4.6	0.37	7.44
Undecane	18.72	19.15	-0.43	-2.30
Undecanedioic acid	58.7	61.85	-3.15	-5.37
Undecylbenzene	36.21	36.91	-0.70	-1.93
Vinclozolin	50.78	51.06	-0.28	-0.55
Vinyl acetate	4.68	3.51	1.17	25.00
Vinylidenechloride	0.74	1	-0.26	-35.14
Vinylpropionate	7.09	6.2	0.89	12.55
Z-1-Chloro-2,3,3,3-tetrafluoropropene	-0.93	0.03	-0.96	103.23
Zingiberene	28.73	32.05	-3.32	-11.56
