# Supplementary material for: Calculation of the Vapour Pressure of Organic Molecules by Means of a Group-Additivity Method and Their Resultant Gibbs Free Energy and Entropy of Vaporization at 298.15 K
Source: Molecules. 2021 Feb 17;26(4):1045. doi: 10.3390/molecules26041045 (PMC7922249; doi:10.3390/molecules26041045)
Supplement: Supplementary file 1 [file molecules-26-01045-s001.zip › molecules-1089923-SM-proofed/S04. Experimental vs. calculated deltaS_vap) Data Table.pdf]

Molecule name	deltaS °(vap) exp	deltaS °(vap) calc	Deviation	Dev in %
(-)-Methyl jasmonate	152.47	168	-15.53	-10.19
(+)-trans-Myrtanol	156	165.65	-9.65	-6.19
(Chloromethyl)benzene	115.08	115.75	-0.67	-0.58
1-(2-Aminoethyl)-4-methylpiperazine	146.03	129.67	16.36	11.20
1,1,1,2,2-Pentafluoro-3-methoxypropane	98.41	94.78	3.63	3.69
1,1,1,2,3,3-Hexafluoro-3-(2,2,3,3-tetrafluoropropoxy)propane	116.02	118.93	-2.91	-2.51
1,1,1,2,4,4-Hexafluoro-2-(trifluoromethoxy)butane	102.16	107.36	-5.2	-5.09
1,1,1,2-Tetrachloroethane	103.57	107.4	-3.83	-3.70
1,1,1,2-Tetrafluoro-2-(trifluoromethoxy)butane	95.05	93.48	1.57	1.65
1,1,1,3,3,3-Hexafluoro-2-methoxypropane	100.75	92.13	8.62	8.56
1,1,1-Trichloroethane	93.78	88.98	4.8	5.12
1,1,1-Trichlorotrifluoroethane	91.73	90.36	1.37	1.49
1,1,1-Trifluoro-2-(1,1,2-trifluoroethoxy)ethane	105.08	101.53	3.55	3.38
1,1,1-Trifluoro-2-(2,2,2-trifluoroethoxy)ethane	104.21	90.49	13.72	13.17
1,1,2,2-Tetrachloroethane	112.02	106.72	5.3	4.73
1,1,2,2-Tetrafluoro-1-(2,2,2-trifluoroethoxy)ethane	103.47	98.61	4.86	4.70
1,1,2-Trichloroethane	105.58	103.47	2.11	2.00
1,1,3-Trimethylcyclopentane	96.26	98.81	-2.55	-2.65
1,1-Bis(4-methylphenyl)ethane	144.22	159.65	-15.43	-10.70
1,1-Dichlorobutane	103.27	97.9	5.37	5.20
1,1-Dichloroethane	92.5	85.06	7.44	8.04
1,1-Dichloropropane	94.15	91.46	2.69	2.86
1,1-Dimethylcyclohexane	100.86	99.58	1.28	1.27
1,1-Dimethylcyclopentane	95.66	95.46	0.2	0.21
1,1-Diphenylbutane	150.96	153.65	-2.69	-1.78
1,1-Diphenylethane	131.34	140.43	-9.09	-6.92
1,1-Diphenylpropane	143.28	147.21	-3.93	-2.74
1,2,3,4,7-Pentachlorodibenzo-p-dioxin	113.2	127.75	-14.55	-12.85
1,2,3,4-Tetrachlorobenzene	111.79	120.38	-8.59	-7.68
1,2,3,4-Tetrachlorodibenzofuran	111.96	129.4	-17.44	-15.58
1,2,3,4-Tetrafluorobenzene	103	98.24	4.76	4.62
1,2,3,5-Tetrachlorobenzene	131.41	120.38	11.03	8.39
1,2,3,5-Tetrafluorobenzene	101.29	98.24	3.05	3.01
1,2,3,5-Tetramethylbenzene	113.16	126.35	-13.19	-11.66
1,2,3,7,8-Pentachlorodibenzo-p-dioxin	98.68	127.75	-29.07	-29.46
1,2,3,7-Tetrachlorodibenzofuran	113.53	129.4	-15.87	-13.98
1,2,3,7-Tetrachlorodibenzo-p-dioxin	121.55	119.54	2.01	1.65
1,2,3-Trichlorobenzene	116.42	112.19	4.23	3.63
1,2,3-Trichloropropane	116.28	112.73	3.55	3.05

1,2,4,5-Tetrachlorobenzene	133.89	120.38	13.51	10.09
1,2,4,5-Tetrafluorobenzene	103.17	98.24	4.93	4.78
1,2,4,7,8-Pentachlorodibenzo-p-dioxin	112.12	127.75	-15.63	-13.94
1,2,4-Trichlorobenzene	117.93	112.19	5.74	4.87
1,2,4-Trichlorodibenzo-p-dioxin	109.01	111.32	-2.31	-2.12
1,2,4-Trimethylbenzene	111.79	116.55	-4.76	-4.26
1,2-Bis(dimethylamino)ethane	106.76	106.19	0.57	0.53
1,2-Butanediamine	111.86	123.7	-11.84	-10.58
1,2-Butanediol	159.05	163.98	-4.93	-3.10
1,2-Dibromobenzene	113.37	105.28	8.09	7.14
1,2-Dibromobutane	108.07	106.09	1.98	1.83
1,2-Dibromoethane	104.71	109.31	-4.6	-4.39
1,2-Dibromoheptane	112.02	125.37	-13.35	-11.92
1,2-Dibromopropane	103.44	99.65	3.79	3.66
1,2-Dibromotetrafluoroethane	90.06	85.06	5	5.55
1,2-Dichloro-1,1,2-trifluoroethane	88.55	91.6	-3.05	-3.44
1,2-Dichlorobenzene	110.05	103.97	6.08	5.52
1,2-Dichlorobutane	102.73	100.75	1.98	1.93
1,2-Diethylbenzene	122.22	119.97	2.25	1.84
1,2-Difluorobenzene	95.05	92.74	2.31	2.43
1,2-Dimethylbenzene	104.44	107.13	-2.69	-2.58
1,2-Ethanediamine	105.65	116.99	-11.34	-10.73
1,2-Ethanediol	146.1	149.22	-3.12	-2.14
1,2-Ethanedithiol	112.12	112.63	-0.51	-0.45
1,2-Hexanediol	170.55	176.86	-6.31	-3.70
1,2-Pentadiene	90.16	88.68	1.48	1.64
1,2-Pentanediol	164.78	170.42	-5.64	-3.42
1,2-Propanediol	154.32	157.57	-3.25	-2.11
1,3,5-Trichlorobenzene	136.41	112.19	24.22	17.76
1,3,5-Trifluorobenzene	97.13	95.66	1.47	1.51
1,3,5-Trimethylbenzene	112.19	116.55	-4.36	-3.89
1,3,6,8-Tetrachlorodibenzofuran	117.66	129.4	-11.74	-9.98
1,3,6,8-Tetrachlorodibenzo-p-dioxin	114.44	119.54	-5.1	-4.46
1,3,7,8-Tetrachlorodibenzo-p-dioxin	118.5	119.54	-1.04	-0.88
1,3,7,9-Tetrachlorodibenzofuran	111.76	129.4	-17.64	-15.78
1,3,7-Trichlorodibenzo-p-dioxin	108.87	111.32	-2.45	-2.25
1,3-Butyleneglycol	155.26	163.98	-8.72	-5.62
1,3-Dibromobenzene	117.96	105.28	12.68	10.75
1,3-Dibromopropane	106.99	115.75	-8.76	-8.19
1,3-Dichlorobenzene	107.5	103.97	3.53	3.28
1,3-Dichloropropane	106.49	106.62	-0.13	-0.12
1,3-Diethylbenzene	121.78	119.97	1.81	1.49
1,3-Dihydroisobenzofuran	120.98	99.38	21.6	17.85

1,3-Diisopropylbenzene	125.91	125	0.91	0.72
1,3-Dimethylbenzene	105.58	107.13	-1.55	-1.47
1,3-Dimethylnaphthalene	143.08	135.74	7.34	5.13
1,3-Dioxane	96.9	91.63	5.27	5.44
1,3-Dioxolane	99.58	84.92	14.66	14.72
1,3-Pentanediamine	132.72	130.14	2.58	1.94
1,3-Propanediamine	125.64	123.39	2.25	1.79
1,3-Propanediol	162.03	155.66	6.37	3.93
1,4-Butanediamine	132.89	129.83	3.06	2.30
1,4-Butanedithiol	126.25	125.47	0.78	0.62
1,4-Dichlorobenzene	109.98	103.97	6.01	5.46
1,4-Dichlorobutane	112.56	113.06	-0.5	-0.44
1,4-Diethylbenzene	121.21	119.97	1.24	1.02
1,4-Diisopropylbenzene	122.69	125	-2.31	-1.88
1,4-Dimethylbenzene	104.95	107.13	-2.18	-2.08
1,4-Dimethylpiperazine	102.5	100.32	2.18	2.13
1,4-Dioxane	103.27	101.09	2.18	2.11
1,4-Di-t-butylbenzene	127.39	123.16	4.23	3.32
1,4-Pentadiene	84.25	88.81	-4.56	-5.41
1,5-Diamino-2-methylpentane	139.76	138.62	1.14	0.82
1,5-Dichloropentane	115.75	119.5	-3.75	-3.24
1,5-Pentanediamine	135.03	136.27	-1.24	-0.92
1,5-Pentanediol	177.63	168.51	9.12	5.13
1,5-Pentanedithiol	133.19	131.91	1.28	0.96
1,6-Hexanediamine	147.27	142.71	4.56	3.10
1,6-Hexanedioic acid	192.52	192.05	0.47	0.24
1,6-Hexanedithiol	142.28	138.35	3.93	2.76
1,7-Heptanediamine	153.01	149.12	3.89	2.54
1,7-Heptanediol	204.09	181.39	22.7	11.12
1,7-Heptanedithiol	152.98	144.76	8.22	5.37
1,8-Cineole	104.95	99.61	5.34	5.09
1,8-Octanediamine	158.81	155.56	3.25	2.05
1-Adamantanol	160.59	152.74	7.85	4.89
1-Amino-3-methoxypropane	114.57	119.6	-5.03	-4.39
1-Azidooctane	132.92	132.25	0.67	0.50
1-Benzylpyrazole	154.85	168.81	-13.96	-9.02
1-Bromo-2-chloro-1,1,2-trifluoroethane	92.1	95.25	-3.15	-3.42
1-Bromo-2-fluorobenzene	113.03	99.18	13.85	12.25
1-Bromo-3-fluorobenzene	107.23	99.18	8.05	7.51
1-Bromo-4-chlorobenzene	114.54	104.44	10.1	8.82
1-Bromo-4-fluorobenzene	104.88	99.18	5.7	5.43
1-Bromobutane	97.87	99.21	-1.34	-1.37
1-Bromodecane	142.98	137.78	5.2	3.64

1-Bromododecane	149.62	150.63	-1.01	-0.68
1-Bromonaphthalene	120.91	125.21	-4.3	-3.56
1-Bromooctane	124	124.94	-0.94	-0.76
1-Bromopentane	103.1	105.62	-2.52	-2.44
1-Bromoperfluorooctane	111.45	118.3	-6.85	-6.15
1-Bromopropane	88.51	92.77	-4.26	-4.81
1-Bromoundecane	143.42	144.22	-0.8	-0.56
1-Butanethiol	99.08	100.69	-1.61	-1.62
1-Butanol	131.38	136.98	-5.6	-4.26
1-Butene	74.39	79.15	-4.76	-6.40
1-Butylamine	97.5	103.04	-5.54	-5.68
1-Butyne	84.66	83.75	0.91	1.07
1-Chloro-2-fluorobenzene	111.22	98.34	12.88	11.58
1-Chloro-3-fluorobenzene	110.55	98.34	12.21	11.04
1-Chloro-4-fluorobenzene	113.3	98.34	14.96	13.20
1-Chlorobutane	91.33	94.31	-2.98	-3.26
1-Chlorodecane	138.29	133.22	5.07	3.67
1-Chlorodibenzo-p-dioxin	122.96	94.92	28.04	22.80
1-Chlorododecane	137.95	146.1	-8.15	-5.91
1-Chloroheptane	114.14	113.94	0.2	0.18
1-Chlorohexane	106.99	107.5	-0.51	-0.48
1-Chloronaphthalene	123.23	124.37	-1.14	-0.93
1-Chlorononane	121.05	126.78	-5.73	-4.73
1-Chlorooctane	120.24	120.38	-0.14	-0.12
1-Chloropentadecane	198.39	165.72	32.67	16.47
1-Chloropentane	103.54	101.06	2.48	2.40
1-Chloropropane	88.18	87.88	0.3	0.34
1-Chlorotridecane	169.68	152.51	17.17	10.12
1-Chloroundecane	134.13	139.66	-5.53	-4.12
1-Decene	118.5	118.06	0.44	0.37
1-Decylazide	143.69	145.13	-1.44	-1.00
1-Difluoromethoxy-1,1,2-trifluoroethane	100.35	95.09	5.26	5.24
1-Dodecanol	188.16	188.76	-0.6	-0.32
1-Dodecene	135.33	131.24	4.09	3.02
1-Ethoxy-1,1,2,2-tetrafluoroethane	100.29	97.2	3.09	3.08
1-Ethyl-1-methylcyclopentane	99.85	102.9	-3.05	-3.05
1-Ethylnaphthalene	138.05	132.72	5.33	3.86
1-Ethylpyrazole	139.9	134.83	5.07	3.62
1-Ethylthiooctane	139.23	135.8	3.43	2.46
1-Fluorononane	120.61	125.21	-4.6	-3.81
1-Fluorooctane	126.31	118.77	7.54	5.97
1-Heneicosanol	252.52	246.96	5.56	2.20
1-Heptadecanol	220.93	220.9	0.03	0.01

1-Heptanethiol	116.89	119.97	-3.08	-2.63
1-Heptanol	157.1	156.26	0.84	0.53
1-Heptene	97.9	98.78	-0.88	-0.90
1-Heptyne	102.3	103.37	-1.07	-1.05
1-Hexadecanol	215.26	214.46	0.8	0.37
1-Hexanethiol	112.93	113.53	-0.6	-0.53
1-Hexanol	144.09	149.82	-5.73	-3.98
1-Hexene	86.73	92.34	-5.61	-6.47
1-Hexyne	98.34	96.93	1.41	1.43
1H-Perfluorooctane	113.63	121.01	-7.38	-6.49
1-Iodobutane	101.76	104.95	-3.19	-3.13
1-Iododecane	143.55	143.89	-0.34	-0.24
1-Iodododecane	156.36	156.73	-0.37	-0.24
1-Iodoheptane	122.62	124.6	-1.98	-1.61
1-Iodohexane	115.11	117.83	-2.72	-2.36
1-Iodononane	135.37	137.45	-2.08	-1.54
1-Iodooctane	128.83	131.01	-2.18	-1.69
1-Iodopentane	106.05	111.39	-5.34	-5.04
1-Iodoundecane	148.42	150.29	-1.87	-1.26
1-Isobutylimidazole	131.54	131.61	-0.07	-0.05
1-Isopropylimidazole	123.6	122.76	0.84	0.68
1-Methyl-1H-pyrazole	125.98	132.28	-6.3	-5.00
1-Methyl-3-phenoxybenzene	140.1	132.85	7.25	5.17
1-Methyl-4-benzylbenzene	134.7	147.54	-12.84	-9.53
1-Methyl-4-isopropylcyclohexene	112.69	97.33	15.36	13.63
1-Methylfluorene	141.07	121.01	20.06	14.22
1-Methylnaphthalene	131.95	126.28	5.67	4.30
1-Methylphenanthrene	154.18	155.22	-1.04	-0.67
1-Methylpiperazine	114.57	110.31	4.26	3.72
1-Naphthylamine	135.27	140.53	-5.26	-3.89
1-Nitro-2-isopropylbenzene	139.59	134.83	4.76	3.41
1-Nitrobutane	117.29	121.55	-4.26	-3.63
1-Nitropentane	118.73	127.99	-9.26	-7.80
1-Nitropropane	115.85	115.11	0.74	0.64
1-Nonanol	164.21	169.11	-4.9	-2.98
1-Nonene	108.97	111.62	-2.65	-2.43
1-Octadecanol	227.91	227.34	0.57	0.25
1-Octanol	156.8	162.7	-5.9	-3.76
1-Octyne	108	109.81	-1.81	-1.68
1-Pentadecanol	205.27	208.05	-2.78	-1.35
1-Pentadecene	153.98	150.86	3.12	2.03
1-Pentanethiol	104.51	107.13	-2.62	-2.51
1-Pentanol	138.25	143.38	-5.13	-3.71

1-Pentene	79.99	85.9	-5.91	-7.39
1-Pentyne	90.63	90.16	0.47	0.52
1-Phenyldodecane	171.46	168.74	2.72	1.59
1-Phenylimidazole	145.97	144.93	1.04	0.71
1-Phenylnaphthalene	153.58	160.25	-6.67	-4.34
1-Piperazineethanamine	152.07	139.69	12.38	8.14
1-Propanethiol	89.92	94.25	-4.33	-4.82
1-Propanol	129.13	130.54	-1.41	-1.09
1-Propylamine	91.9	96.26	-4.36	-4.74
1-s-Butylimidazole	139.09	129.2	9.89	7.11
1-t-Butylimidazole	133.36	129.3	4.06	3.04
1-Tetradecene	146.74	144.12	2.62	1.79
1-Tetradecylamine	180.92	167.67	13.25	7.32
1-trans-5-trans-9-cis-Cyclododecatriene	152.64	132.22	20.42	13.38
1-Tridecanol	195.61	195.17	0.44	0.22
1-Tridecene	141.3	137.68	3.62	2.56
1-Undecanol	176.89	182.32	-5.43	-3.07
1-Undecene	124.7	124.84	-0.14	-0.11
2,2,2-Trifluoroethanol	127.75	125.51	2.24	1.75
2,2',3,3',4,4',6-Heptachlorobiphenyl	182.09	188.43	-6.34	-3.48
2,2',3,3',4-Pentabromodiphenyl ether	153.85	167.9	-14.05	-9.13
2,2,3,3-Tetramethylhexane	108.03	100.99	7.04	6.52
2,2,3,3-Tetramethylpentane	102	93.54	8.46	8.29
2,2',3,4,5,5',6-Heptabromodiphenyl ether	178.77	185.61	-6.84	-3.83
2,2,3,4-Tetramethylpentane	102.97	97	5.97	5.80
2,2',3,5',6-Pentachlorobiphenyl	170.22	172.03	-1.81	-1.06
2,2,3-Trimethylpentane	100.12	93.64	6.48	6.47
2,2',4,4',5,5'-Hexabromodiphenyl ether	172.87	176.92	-4.05	-2.34
2,2',4,4',5,5'-Hexachlorobiphenyl	175.42	180.21	-4.79	-2.73
2,2',4,4',5,6'-Hexabromodiphenyl ether	179.41	176.92	2.49	1.39
2,2',4,4',5-Pentabromodiphenyl ether	184.07	167.9	16.17	8.78
2,2',4,4'-Tetrabromodiphenyl ether	152.51	158.85	-6.34	-4.16
2,2,4,4-Tetramethylpentane	98.71	93.54	5.17	5.24
2,2',4,5',6-Pentachlorobiphenyl	169.08	172.03	-2.95	-1.74
2,2',4,5'-Tetrachlorobiphenyl	160.76	163.81	-3.05	-1.90
2,2,4-Trimethyl-4-methoxypentane	110.98	103.47	7.51	6.77
2,2,4-Trimethylhexane	104.18	101.09	3.09	2.97
2,2,5,5-Tetramethylhexane	108.87	100.99	7.88	7.24
2,2',5,6'-Tetrachlorobiphenyl	144.76	163.81	-19.05	-13.16
2,2',5-Trichlorobiphenyl	149.82	155.59	-5.77	-3.85
2,2,5-Trimethylhexane	102.87	101.09	1.78	1.73
2,2’-Diaminodiethylamine	140.53	151.13	-10.6	-7.54
2,2-Dichloro-1,1,1-trifluoroethane	88.08	86.43	1.65	1.87

2,2-Dimethyl-3-ethylpentane	104.85	101.09	3.76	3.59
2,2-Dimethylheptane	107.23	105.15	2.08	1.94
2,2-Dimethylhexane	99.28	97.7	1.58	1.59
2,2-Dimethyloctane	119.94	112.59	7.35	6.13
2,2-Dimethylpentane	92.03	90.29	1.74	1.89
2,2-Dimethylpropane	77.48	75.4	2.08	2.68
2',3,3',4,4',5,6-Heptabromodiphenyl ether	190.98	185.61	5.37	2.81
2,3,3',4,4',5-Hexachlorobiphenyl	178.94	180.21	-1.27	-0.71
2,3,3,4-Tetramethylpentane	103.2	97	6.2	6.01
2,3,3-Trimethyl-1-butene	99.35	86.97	12.38	12.46
2,3,3-Trimethylhexane	106.19	101.09	5.1	4.80
2,3,3-Trimethylpentane	98.37	93.64	4.73	4.81
2,3',4,4'-Tetrabromodiphenyl ether	146.54	158.85	-12.31	-8.40
2,3,4,5,6-Pentafluorotoluene	106.96	110.62	-3.66	-3.42
2,3',4,6-Tetrabromodiphenyl ether	142.81	158.85	-16.04	-11.23
2',3,4-Tribromodiphenyl ether	121.72	149.82	-28.1	-23.09
2,3,4-Trimethylpentane	99.08	97.1	1.98	2.00
2,3,7-Trichlorodibenzo-p-dioxin	129.73	111.32	18.41	14.19
2,3-Butandione	108.17	118.13	-9.96	-9.21
2,3-Butyleneglycol	154.05	165.89	-11.84	-7.69
2,3-Dichlorobutane	100.15	88.75	11.4	11.38
2,3-Dichlorodibenzo-p-dioxin	99.85	103.14	-3.29	-3.29
2,3-Dimethyl-1,3-butadiene	90.56	94.52	-3.96	-4.37
2,3-Dimethyl-1-butene	88.78	87.74	1.04	1.17
2,3-Dimethyl-1-hexene	101.59	100.92	0.67	0.66
2,3-Dimethyl-2-butanol	135.57	141.44	-5.87	-4.33
2,3-Dimethyl-2-pentene	96.63	101.63	-5	-5.17
2,3-Dimethylbutane	88.04	86.3	1.74	1.98
2,3-Dimethylhexane	102	101.16	0.84	0.82
2,3-Dimethylpentane	95.02	93.71	1.31	1.38
2,3-Lutidine	127.39	111.62	15.77	12.38
2,3-Pentadiene	91.67	92.6	-0.93	-1.01
2,4,6-Collidine	118.97	114.64	4.33	3.64
2,4,6-Tribromodiphenyl ether	143.62	149.82	-6.2	-4.32
2,4',6-Tribromodiphenyl ether	131.48	149.82	-18.34	-13.95
2,4,6-Trichloroanisole	127.89	139.8	-11.91	-9.31
2,4,6-Trichlorodibenzofuran	131.44	121.18	10.26	7.81
2,4,8-Trichlorodibenzofuran	113.37	121.18	-7.81	-6.89
2,4'-Dibromodiphenyl ether	123.9	141.14	-17.24	-13.91
2,4-Dibromodiphenyl ether	122.32	141.14	-18.82	-15.39
2,4-Dichlorobiphenyl	142.24	147.41	-5.17	-3.63
2,4-Dimethyl-3-ethylpentane	109.24	104.51	4.73	4.33
2,4-Dimethyl-3-pentanone	112.06	108.3	3.76	3.36

2,4-Dimethylhexane	100.02	101.16	-1.14	-1.14
2,4-Dimethylpentane	92.67	93.71	-1.04	-1.12
2,4-Lutidine	113.57	111.62	1.95	1.72
2,4-Xylenol	142.08	163.51	-21.43	-15.08
2,5-Aldehydine	103.4	118.06	-14.66	-14.18
2,5-Dichlorobiphenyl	147.48	147.41	0.07	0.05
2,5-Dimethylhexane	100.32	101.16	-0.84	-0.84
2,5-Dimethyltetrahydrofuran	96.96	96.9	0.06	0.06
2,5-Dimethylthiophene	97.7	109.41	-11.71	-11.99
2,6-Dibromodiphenyl ether	118.87	141.14	-22.27	-18.73
2,6-Dichlorotoluene	112.56	113.43	-0.87	-0.77
2,6-Diethylaniline	119.44	144.02	-24.58	-20.58
2,6-Dimethyl-4-heptanol	153.58	163.21	-9.63	-6.27
2,6-Dimethylheptan-4-one	120.14	121.52	-1.38	-1.15
2,6-Dimethylheptane	108.64	108.6	0.04	0.04
2,6-Lutidine	111.15	105.22	5.93	5.34
2,6-Xylenol	182.16	163.51	18.65	10.24
2,6-Xylidine	127.62	131.18	-3.56	-2.79
2,7-Dibromofluorene	159.79	128.96	30.83	19.29
2,7-Diiodofluorene	168.57	164.62	3.95	2.34
2,7-Dimethyloctane	115.78	116.05	-0.27	-0.23
2-Adamantanone	111.99	101.19	10.8	9.64
2-Azidoacetonitrile	127.75	124.37	3.38	2.65
2-Azidoethanol	148.21	154.42	-6.21	-4.19
2-Azidoethoxyethane	120.17	118.03	2.14	1.78
2-Bromo-7-chlorofluorene	157.2	128.12	29.08	18.50
2-Bromo-7-iodofluorene	164.25	146.81	17.44	10.62
2-Bromobutane	94.48	83.08	11.4	12.07
2-Bromofluorene	144.79	119.94	24.85	17.16
2-Bromonaphthalene	120.11	125.21	-5.1	-4.25
2-Bromopropane	90.86	76.67	14.19	15.62
2-Bromotoluene	107.6	106.02	1.58	1.47
2-Butanethiol	95.72	96.56	-0.84	-0.88
2-Butanol	130.71	138.89	-8.18	-6.26
2-Butanone	99.68	96.83	2.85	2.86
2-Butyne	88.95	86.2	2.75	3.09
2-Chloro-1,1,2-trifluoroethyl difluoromethyl ether	98.84	99.65	-0.81	-0.82
2-Chloro-1,1,2-trifluoroethyl ethyl ether	104.41	104.38	0.03	0.03
2-Chloro-1,1,2-trifluoroethyl propyl ether	108.7	110.78	-2.08	-1.91
2-Chloroaniline	122.92	120.14	2.78	2.26
2-Chlorobiphenyl	146.94	139.19	7.75	5.27
2-Chlorobutane	92.84	82.31	10.53	11.34
2-Chloroethanol	123.23	142.55	-19.32	-15.68

2-Chloronaphthalene	124.3	124.37	-0.07	-0.06
2-Chloronitrobenzene	120.91	124.47	-3.56	-2.94
2-Chloropentane	97.84	88.75	9.09	9.29
2-Chloropropane	89.22	75.9	13.32	14.93
2-Chloropropanoic acid	151.57	134.16	17.41	11.49
2-Chloropyridine	122.42	95.52	26.9	21.97
2-Chlorotoluene	110.95	105.22	5.73	5.16
2-Difluoromethoxy-1,1,1,3,3,3-hexafluoropropane	90.83	94.58	-3.75	-4.13
2-Dimethylaminoethanethiol	103.4	109.41	-6.01	-5.81
2-Ethoxyethylacetate	128.89	118.23	10.66	8.27
2-Ethyl-1-butanol	150.56	145.77	4.79	3.18
2-Ethyl-1-butene	91.8	91.8	0	0.00
2-Ethyl-1-hexanol	158.34	158.95	-0.61	-0.39
2-Ethyl-1-pentene	96.23	98.24	-2.01	-2.09
2-Ethyl-m-xylene	125.64	122.99	2.65	2.11
2-Ethylnaphthalene	129	132.72	-3.72	-2.88
2-Ethylnitrobenzene	132.92	132.11	0.81	0.61
2-Ethyl-p-xylene	120.81	122.99	-2.18	-1.80
2-Ethylpyridine	108	108.6	-0.6	-0.56
2-Ethylthiophene	95.89	108.4	-12.51	-13.05
2-Ethyltoluene	109.91	113.53	-3.62	-3.29
2-Fluoroiodobenzene	117.02	116.99	0.03	0.03
2-Fluoronitrobenzene	128.89	118.83	10.06	7.81
2-Heptanol	150.66	158.18	-7.52	-4.99
2-Heptanone	110.58	116.45	-5.87	-5.31
2-Hexanol	143.59	151.74	-8.15	-5.68
2-Hexyne	101.69	99.04	2.65	2.61
2-Iodofluorene	151.64	137.75	13.89	9.16
2-Isopropyltoluene	118.23	115.91	2.32	1.96
2-Methoxyethylamine	105.95	113.2	-7.25	-6.84
2-Methoxy-p-cresol	151.53	181.65	-30.12	-19.88
2-Methyl-1,1-diphenylpropane	139.09	149.56	-10.47	-7.53
2-Methyl-1,2-propanediamine	122.15	123.8	-1.65	-1.35
2-Methyl-1-butanol	135.7	139.33	-3.63	-2.68
2-Methyl-1-heptene	101.83	105.01	-3.18	-3.12
2-Methyl-1-hexene	96.73	98.24	-1.51	-1.56
2-Methyl-1-pentanol	148.88	145.77	3.11	2.09
2-Methyl-1-pentene	90.83	91.8	-0.97	-1.07
2-Methyl-1-propanethiol	99.18	96.6	2.58	2.60
2-Methyl-1-propanol	129.23	132.89	-3.66	-2.83
2-Methyl-2-butanol	140.97	138.72	2.25	1.60
2-Methyl-2-pentanol	143.65	145.16	-1.51	-1.05
2-Methyl-2-pentene	92.91	95.72	-2.81	-3.02

2-Methyl-2-propanol	126.38	132.32	-5.94	-4.70
2-Methyl-3-pentanol	146.67	147.98	-1.31	-0.89
2-Methylaminoethanol	138.05	155.73	-17.68	-12.81
2-Methylanthracene	153.51	155.22	-1.71	-1.11
2-Methylbenzaldehyde	114.74	120.41	-5.67	-4.94
2-Methylheptane	103.17	105.25	-2.08	-2.02
2-Methylhexane	95.99	97.8	-1.81	-1.89
2-Methylnonane	121.18	119.77	1.41	1.16
2-Methyloctane	110.78	112.36	-1.58	-1.43
2-Methylpentane	89.59	90.36	-0.77	-0.86
2-Methylpropanal	93.28	85.76	7.52	8.06
2-Methylpropane	71.21	75.5	-4.29	-6.02
2-Methylpropanoic acid	139.12	130.4	8.72	6.27
2-Methylpropylamine	99.61	98.94	0.67	0.67
2-Methylpyrazine	108.87	113.43	-4.56	-4.19
2-Methyltetrahydrofuran	100.55	93.91	6.64	6.60
2-Methylthiophene	101.32	101.66	-0.34	-0.34
2-Nitrobutane	117.52	114.64	2.88	2.45
2-Nitro-m-xylene	127.18	135.47	-8.29	-6.52
2-Nitropropane	115.55	108.2	7.35	6.36
2-Nitro-t-butylbenzene	135.2	133.72	1.48	1.09
2-Nitrotoluene	126.11	125.71	0.4	0.32
2-Nonanol	168.37	171.36	-2.99	-1.78
2-Nonanone	128.02	129.33	-1.31	-1.02
2-n-Propylthiophene	100.89	114.84	-13.95	-13.83
2-Octanol	161.13	164.92	-3.79	-2.35
2-Octanone	123.76	122.89	0.87	0.70
2-Pentanol	139.76	145.3	-5.54	-3.96
2-Pentyne	93.54	92.6	0.94	1.00
2-Phenylethylamine	125.27	130.57	-5.3	-4.23
2-Picoline	111.22	102.2	9.02	8.11
2-Propanethiol	90.56	90.12	0.44	0.49
2-Propanol	124.4	132.45	-8.05	-6.47
2-Propyltoluene	121.45	119.97	1.48	1.22
2-Pyrrolidone	143.12	130.61	12.51	8.74
2-t-Butylphenol	182.49	162.07	20.42	11.19
3-(Difluoromethoxy)-1,1,1,2,2-pentafluoropropane	97.87	91.1	6.77	6.92
3,3,3-Trifluoropropene	88.18	74.09	14.09	15.98
3,3',4,4'-Tetrabromodiphenyl ether	160.15	158.85	1.3	0.81
3,3',4-Tribromodiphenyl ether	137.68	149.82	-12.14	-8.82
3,3,5-Trimethylheptane	111.02	108.5	2.52	2.27
3,3'-Bitolyl	137.78	150.86	-13.08	-9.49
3,3'-Dichlorobiphenyl	155.83	147.41	8.42	5.40

3,3-Dimethyl-1-butene	84.42	80.73	3.69	4.37
3,3-Dimethyl-1-pentene	95.72	87.51	8.21	8.58
3,3-Dimethylhexane	98.41	97.7	0.71	0.72
3,3-Dimethylpentane	92.1	90.29	1.81	1.97
3,4,4'-Tribromodiphenyl ether	135.67	149.82	-14.15	-10.43
3,4'-Dibromodiphenyl ether	124.2	141.14	-16.94	-13.64
3,4-Dibromodiphenyl ether	126.04	141.14	-15.1	-11.98
3,4-Dichloroaniline	144.73	128.36	16.37	11.31
3,4-Dichloronitrobenzene	125.71	132.65	-6.94	-5.52
3,4-Dichlorophenol	148.82	160.69	-11.87	-7.98
3,4-Dimethylhexane	101.22	101.16	0.06	0.06
3,5-Lutidine	112.02	118.06	-6.04	-5.39
3-Bromochlorobenzene	114.47	104.44	10.03	8.76
3-Bromodiphenyl ether	106.22	132.11	-25.89	-24.37
3-Bromotoluene	107.97	106.02	1.95	1.81
3-Chloroaniline	127.08	120.14	6.94	5.46
3-Chlorobiphenyl	146.6	139.19	7.41	5.05
3-Chloronitrobenzene	121.31	124.47	-3.16	-2.60
3-Chlorophenol	140.5	152.47	-11.97	-8.52
3-Chlorotoluene	112.63	105.22	7.41	6.58
3-Difluoromethoxy-1,1,2,2-tetrafluoropropane	103.54	103.61	-0.07	-0.07
3-Ethyl-1-pentene	96.46	94.68	1.78	1.85
3-Ethyl-2-methylpentane	100.35	101.16	-0.81	-0.81
3-Ethyl-3-methylpentane	98.37	97.7	0.67	0.68
3-Ethylheptane	116.92	112.36	4.56	3.90
3-Ethylhexane	102.93	105.25	-2.32	-2.25
3-Ethyl-o-xylene	125.37	122.99	2.38	1.90
3-Ethylpentane	96.29	97.8	-1.51	-1.57
3-Ethyltoluene	110.78	113.53	-2.75	-2.48
3-Fluorobenzotrifluoride	102.3	101.32	0.98	0.96
3-Fluoronitrobenzene	121.68	118.83	2.85	2.34
3-Hexanol	149.82	151.74	-1.92	-1.28
3-Hexanone	102.87	110.05	-7.18	-6.98
3-Hexyne	102.77	99.04	3.73	3.63
3-Isopropyltoluene	116.02	115.91	0.11	0.09
3-Methoxysalicylaldehyde	142.95	129.57	13.38	9.36
3-Methyl-1,2-butadiene	89.12	88.48	0.64	0.72
3-Methyl-1-butene	81.4	81.84	-0.44	-0.54
3-Methyl-1-butyne	85.33	86.43	-1.1	-1.29
3-Methyl-1-hexene	96.56	94.68	1.88	1.95
3-Methyl-1-pentene	87.14	88.24	-1.1	-1.26
3-Methyl-2-butanol	136.31	141.57	-5.26	-3.86
3-Methyl-2-butanone	101.22	99.51	1.71	1.69

3-Methyl-2-butenyl acetate	115.61	119.47	-3.86	-3.34
3-Methyl-2-pentanol	149.82	147.98	1.84	1.23
3-Methyl-3-pentanol	145.87	145.16	0.71	0.49
3-Methylbenzaldehyde	115.91	120.41	-4.5	-3.88
3-Methylbutanoic acid	143.62	136.84	6.78	4.72
3-Methyl-cis-2-pentene	91.6	95.72	-4.12	-4.50
3-Methylcyclopentene	73.35	74.93	-1.58	-2.15
3-Methyleneheptane	102.9	105.01	-2.11	-2.05
3-Methylheptane	102.87	105.25	-2.38	-2.31
3-Methylhexane	96.66	97.8	-1.14	-1.18
3-Methylnonane	118.9	119.77	-0.87	-0.73
3-Methyloctane	110.98	112.36	-1.38	-1.24
3-Methylpentane	90.06	90.36	-0.3	-0.33
3-Methylpyridine	108.94	108.6	0.34	0.31
3-Methylthiophene	103.2	100.12	3.08	2.98
3-Methyl-trans-2-pentene	93.58	95.72	-2.14	-2.29
3-Nitrotoluene	125.47	125.71	-0.24	-0.19
3-Nonanol	164.51	171.36	-6.85	-4.16
3-Octanol	163.27	164.92	-1.65	-1.01
3-Pentanol	141.17	145.3	-4.13	-2.93
3-Propyltoluene	120.71	119.97	0.74	0.61
3-t-Butylphenol	144.56	162.07	-17.51	-12.11
4,4,6-Trimethyl-1,3-dioxane	112.8	97.6	15.2	13.48
4,4'-Dichlorobiphenyl	155.39	147.41	7.98	5.14
4,4-Dimethyl-1-pentene	90.29	87.51	2.78	3.08
4-Bromophenol	145.23	153.31	-8.08	-5.56
4-Bromotoluene	104.54	106.02	-1.48	-1.42
4-Chloroaniline	123.66	120.14	3.52	2.85
4-Chlorobiphenyl	146.67	139.19	7.48	5.10
4-Chloronitrobenzene	121.88	124.47	-2.59	-2.13
4-Chlorophenol	144.99	152.47	-7.48	-5.16
4-Chlorotoluene	109.24	105.22	4.02	3.68
4-Ethyl-m-xylene	122.66	122.99	-0.33	-0.27
4-Ethyl-o-xylene	125.84	122.99	2.85	2.26
4-Ethylpyridine	106.93	115.04	-8.11	-7.58
4-Ethyltoluene	109.84	113.53	-3.69	-3.36
4-Fluoronitrobenzene	123.53	118.83	4.7	3.80
4-Fluorophenol	138.08	147.21	-9.13	-6.61
4-Heptanol	154.12	158.18	-4.06	-2.63
4-Heptanone	106.93	116.45	-9.52	-8.90
4-Iodophenol	155.26	171.12	-15.86	-10.22
4-Methyl-1-hexene	97.07	94.68	2.39	2.46
4-Methyl-2-pentanol	150.73	147.98	2.75	1.82

4-Methylbenzaldehyde	113	120.41	-7.41	-6.56
4-Methyl-cis-2-pentene	89.49	91.87	-2.38	-2.66
4-Methylcyclopentene	77.08	74.93	2.15	2.79
4-Methylheptane	103.1	105.25	-2.15	-2.09
4-Methylnonane	117.86	119.77	-1.91	-1.62
4-Methyloctane	110.08	112.36	-2.28	-2.07
4-Methylpent-3-en-2-one	108	116.35	-8.35	-7.73
4-Methylpyridine	109.54	108.6	0.94	0.86
4-Methyl-trans-2-pentene	90.29	91.87	-1.58	-1.75
4-Nitrotoluene	132.75	125.71	7.04	5.30
4-Propyltoluene	119.74	119.97	-0.23	-0.19
4-t-Butylphenol	142.61	162.07	-19.46	-13.65
5-Ethyl-m-xylene	115.65	122.99	-7.34	-6.35
5-Methyl-1-hexene	96.09	94.68	1.41	1.47
5-Methylnonane	118.43	119.77	-1.34	-1.13
5-Nonanone	124.57	129.33	-4.76	-3.82
6-Methyl-1-heptene	93.91	101.12	-7.21	-7.68
9,10-Benzophenanthrene	185.64	174.41	11.23	6.05
Acenaphthene	120.14	110.98	9.16	7.62
Acenaphthylene	118.7	108.07	10.63	8.96
Acetaldehyde	91.56	76.97	14.59	15.93
Acetic acid	134.26	121.65	12.61	9.39
Acetonitrile	92.87	94.05	-1.18	-1.27
Acetophenone	122.92	124.74	-1.82	-1.48
Acetylacetone	106.62	124.57	-17.95	-16.84
Acetylchloride	76.97	90.02	-13.05	-16.95
Acrylic acid	135.03	130.97	4.06	3.01
Adamantane	101.63	101.63	0	0.00
Adiponitrile	182.42	150.8	31.62	17.33
Allyl alcohol	122.19	133.46	-11.27	-9.22
Allyl chloride	94.18	91.13	3.05	3.24
Allyl cyanide	103.24	110.18	-6.94	-6.72
Allyl hexanoate	125.94	129.26	-3.32	-2.64
alpha,alpha-Dimethylphenethylamine	134.9	137.38	-2.48	-1.84
alpha-Curcumene	148.45	155.06	-6.61	-4.45
alpha-Methyl benzylamine	126.31	124.1	2.21	1.75
alpha-Methylstyrene	115.38	113.27	2.11	1.83
alpha-Pinene	106.69	102.67	4.02	3.77
Amyl acetate	117.42	113.16	4.26	3.63
Amyl propionate	130.54	119.6	10.94	8.38
Amylamine	104.95	109.48	-4.53	-4.32
Amylbenzene	120.48	123.39	-2.91	-2.42
Aniline	128.76	111.92	16.84	13.08

Anthracene	149.22	145.46	3.76	2.52
Arachic alcohol	242.63	240.52	2.11	0.87
Azepane	119.81	118.43	1.38	1.15
Azetidine	102.23	98.31	3.92	3.83
Azidocyclopentane	103.04	111.66	-8.62	-8.37
Aziridine	102.97	84.86	18.11	17.59
Azocane	113.13	131.01	-17.88	-15.80
Benz[a]anthracene	187.62	174.41	13.21	7.04
Benz[e]acephenanthrylene	181.05	160.72	20.33	11.23
Benzaldehyde	111.12	110.95	0.17	0.15
Benzene	96.03	87.57	8.46	8.81
Benzo[a]phenanthrene	188.56	174.41	14.15	7.50
Benzo[a]pyrene	178.13	179.04	-0.91	-0.51
Benzo[b]fluorene	164.08	140.2	23.88	14.55
Benzo[b]triphenylene	224.18	203.35	20.83	9.29
Benzo[e]pyrene	183.77	179.04	4.73	2.57
Benzo[ghi]perylene	183.33	184.03	-0.7	-0.38
Benzonitrile	128.53	128.02	0.51	0.40
Benzotrifluoride	99.55	98.71	0.84	0.84
Benzoyl chloride	124.74	124	0.74	0.59
Benzyl acetate	116.55	127.86	-11.31	-9.70
Benzyl alcohol	146.37	158.07	-11.7	-7.99
Benzyl benzoate	136.14	161.83	-25.69	-18.87
Benzyl butyl phthalate	205.5	214.99	-9.49	-4.62
Benzyl ethyl ether	123.63	121.72	1.91	1.54
Benzylamine	123.8	124.13	-0.33	-0.27
beta-Sesquiphellandrene	156.4	133.19	23.21	14.84
Bicifadine	152.14	148.28	3.86	2.54
Bicyclohexyl	120.85	134.66	-13.81	-11.43
Biphenyl	133.99	131.65	2.34	1.75
Biphenylene	118.73	120.88	-2.15	-1.81
Bromobenzene	104.95	96.6	8.35	7.96
Bromocyclohexane	106.12	97.84	8.28	7.80
Butanal	95.29	89.85	5.44	5.71
Butane	81.84	79.56	2.28	2.79
Butanedioic acid	172.73	179.21	-6.48	-3.75
Butanoic acid	140.9	134.5	6.4	4.54
Butyl 1,1-dimethylpropyl ether	110.88	109.68	1.2	1.08
Butyl 4-oxopentanoate	131.88	146.97	-15.09	-11.44
Butyl butanoate	129.5	119.6	9.9	7.64
Butyl propanoate	120.68	113.16	7.52	6.23
Butyl t-octyl ether	111.45	117.69	-6.24	-5.60
Butylacetamide	170.69	154.22	16.47	9.65

Butylbenzene	115.51	116.95	-1.44	-1.25
Butylcyclohexane	114.81	121.65	-6.84	-5.96
Butylcyclopentane	109.88	117.52	-7.64	-6.95
Butylmethylketone	110.11	110.05	0.06	0.05
Butyric acid methylester	109.84	105.38	4.46	4.06
Butyronitrile	101.02	106.93	-5.91	-5.85
Camphene	105.82	96.66	9.16	8.66
Camphor	112.8	107.06	5.74	5.09
Capraldehyde	128.39	128.76	-0.37	-0.29
Caproic aldehyde	107.4	102.7	4.7	4.38
Caprylene	104.58	105.18	-0.6	-0.57
Caprylic aldehyde	117.16	115.58	1.58	1.35
Carvone	120.54	118.56	1.98	1.64
Cedrol	178.2	167.37	10.83	6.08
Cetane	163.37	168.14	-4.77	-2.92
Cetene	164.98	157.3	7.68	4.66
Chloral	115.11	102.6	12.51	10.87
Chlorfenvinphos	169.85	179.17	-9.32	-5.49
Chloroacetic acid ethylester	123.9	112.29	11.61	9.37
Chloroacetyl chloride	119.5	108.44	11.06	9.26
Chlorobenzene	103.61	95.76	7.85	7.58
Chlorocyclohexane	103.4	97.4	6	5.80
Chloroethane	84.59	81.44	3.15	3.72
Chloroethylene	77.58	75.4	2.18	2.81
Chloropentafluorobenzene	107.29	109.04	-1.75	-1.63
cis 3-Hexenyl butyrate	132.08	139.29	-7.21	-5.46
cis- Cyclooctene	101.16	104.81	-3.65	-3.61
cis-1,2-Dichloroethylene	92.71	88.45	4.26	4.59
cis-1,2-Dimethylcyclohexane	99.21	103.04	-3.83	-3.86
cis-1,2-Dimethylcyclopentane	97.1	98.91	-1.81	-1.86
cis-1,3-Dimethylcyclohexane	98.14	103.04	-4.9	-4.99
cis-1,3-Dimethylcyclopentane	97.07	98.91	-1.84	-1.90
cis-1,3-Pentadiene	89.15	92.4	-3.25	-3.65
cis-1,4-Dimethylcyclohexane	99.65	103.04	-3.39	-3.40
cis-2-Butene	80.36	83.08	-2.72	-3.38
cis-2-Heptene	106.56	102.36	4.2	3.94
cis-2-Hexene	92.13	95.92	-3.79	-4.11
cis-2-Methylcyclohexanol	160.42	156.3	4.12	2.57
cis-2-Octene	103.74	109.14	-5.4	-5.21
cis-2-Pentene	86.33	89.49	-3.16	-3.66
cis-3-Heptene	97.57	102.36	-4.79	-4.91
cis-3-Hexene	91.4	95.92	-4.52	-4.95
cis-3-Hexenyl 2-methylbutyrate	133.12	141.64	-8.52	-6.40

cis-3-Hexenyl propionate	125.84	132.89	-7.05	-5.60
cis-3-Octene	93.71	109.14	-15.43	-16.47
cis-4,6-Dimethyl-1,3-dioxane	99.58	97.9	1.68	1.69
cis-4-Octene	102.06	109.14	-7.08	-6.94
cis-5-Octenyl propionate	139.36	145.73	-6.37	-4.57
cis-Crotononitrile	104.11	113.77	-9.66	-9.28
cis-Stilbene	142.51	151.37	-8.86	-6.22
Citronellyl acetate	143.08	147.88	-4.8	-3.35
Crotonaldehyde	100.42	96.7	3.72	3.70
Cryofluorane	83.55	78.08	5.47	6.55
Cumene	108.47	106.46	2.01	1.85
Cyanoethylene	90.69	103.74	-13.05	-14.39
Cyclobutane	83.85	87.14	-3.29	-3.92
Cyclobutanone	104.68	89.75	14.93	14.26
Cycloheptane	99.61	109.61	-10	-10.04
Cycloheptene	92.87	92.24	0.63	0.68
Cyclohexane	93.88	96.33	-2.45	-2.61
Cyclohexanethiol	107.33	111.29	-3.96	-3.69
Cyclohexanol	151.3	153.95	-2.65	-1.75
Cyclohexanone	113.3	99.55	13.75	12.14
Cyclohexyl azide	117.42	115.14	2.28	1.94
Cyclohexylamine	111.62	111.62	0	0.00
Cyclohexylbenzene	122.19	127.96	-5.77	-4.72
Cyclooctane	104.44	123.19	-18.75	-17.95
Cyclopentane	88.21	92.17	-3.96	-4.49
Cyclopentanol	141.54	150.49	-8.95	-6.32
Cyclopentanone	106.56	90.89	15.67	14.71
Decamethylcyclopentasiloxane	127.35	140.63	-13.28	-10.43
Decane	119.44	123.86	-4.42	-3.70
Decanedioic acid	219.02	218.11	0.91	0.42
Decylbenzene	152	155.89	-3.89	-2.56
Decylcyclohexane	157.61	165.92	-8.31	-5.27
Demeton	151.37	156.26	-4.89	-3.23
Deprenyl	127.45	142.14	-14.69	-11.53
Diamylamine	139.76	136.11	3.65	2.61
Dibenz[ah]anthracene	217.51	203.35	14.16	6.51
Dibenzo[b,d]thiophene	151.1	140.6	10.5	6.95
Dibenzo-p-dioxin	109.48	86.7	22.78	20.81
Dibutyl carbonate	142.04	149.59	-7.55	-5.32
Dibutyl oxalate	148.62	150.09	-1.47	-0.99
Dibutyl phthalate	191.92	193.86	-1.94	-1.01
Dibutylacetamide	148.15	167.03	-18.88	-12.74
Dibutylamine	122.96	123.23	-0.27	-0.22

Dibutylsulfide	128.53	122.62	5.91	4.60
Diethyl disulfide	107.16	109.71	-2.55	-2.38
Diethyl glutarate	148.75	143.69	5.06	3.40
Diethyl malonate	133.56	130.81	2.75	2.06
Diethyl methylphosphonate	125.1	123.73	1.37	1.10
Diethyl oxalate	131.65	124.37	7.28	5.53
Diethyl phthalate	167.33	168.14	-0.81	-0.48
Diethyl succinate	141.87	137.25	4.62	3.26
Diethyl sulphide	98.71	96.9	1.81	1.83
Diethylacetamide	128.32	141.3	-12.98	-10.12
Diethylamine	95.12	97.17	-2.05	-2.16
Diethylcarbonate	132.79	123.56	9.23	6.95
Diethylketone	104.14	103.61	0.53	0.51
Diheptylamine	170.92	162.17	8.75	5.12
Dihexylamine	153.98	148.95	5.03	3.27
Dihydropinene	108.1	117.56	-9.46	-8.75
Diisobutyl ether	106.26	105.28	0.98	0.92
Diisobutylene	96.39	93.41	2.98	3.09
Diisopropyl ether	96.09	93.68	2.41	2.51
Diisopropyl methylphosphonate	129.03	129.67	-0.64	-0.50
Diisopropyl sulfide	102.2	101.49	0.71	0.69
Diisopropylamine	97.17	97.77	-0.6	-0.62
Dimethoate	166.53	179.67	-13.14	-7.89
Dimethyl carbonate	105.42	121.18	-15.76	-14.95
Dimethyl disulfide	99.78	99.95	-0.17	-0.17
Dimethyl hexanedioate	141.1	147.74	-6.64	-4.71
Dimethyl oxalate	124.77	121.68	3.09	2.48
Dimethyl phthalate	160.46	165.45	-4.99	-3.11
Dimethyl sulfide	90.02	87.14	2.88	3.20
Dimethylacetal	110.05	109.11	0.94	0.85
Dimethylacetamide	124.33	136.21	-11.88	-9.56
Dimethylamine	94.52	92.4	2.12	2.24
Dimethylether	76.67	85.02	-8.35	-10.89
Di-n-butylether	109.91	113.43	-3.52	-3.20
Di-n-propyl phthalate	168.04	181.02	-12.98	-7.72
Dioctyl sulfide	193.63	174.41	19.22	9.93
Dioctylamine	181.28	175.01	6.27	3.46
Diphenyl ether	134.36	123.09	11.27	8.39
Diphenylmethane	136.37	138.08	-1.71	-1.25
Dipropyl carbonate	127.08	136.74	-9.66	-7.60
Dipropyl ether	98.94	100.59	-1.65	-1.67
Dipropyl malonate	140.84	143.69	-2.85	-2.02
Dipropyl sulfide	108.87	109.74	-0.87	-0.80

Dipropylamine	108.84	110.38	-1.54	-1.41
Di-s-butylsulfide	109.98	114.37	-4.39	-3.99
Disulfoton	130.4	140.13	-9.73	-7.46
Docosane	209.22	212.11	-2.89	-1.38
Dodecafluorotetrahydrothiophene	93.95	86.3	7.65	8.14
Dodecahydrosqualene	243.43	247.46	-4.03	-1.66
Dodecane	135.5	138.72	-3.22	-2.38
Dodecyl acetate	161.5	158.51	2.99	1.85
Dopentacontane	443.27	433.51	9.76	2.20
Dotetracontane	367.7	359.48	8.22	2.24
Dotriacontane	287.61	285.8	1.81	0.63
d-Verbenone	123.46	115.48	7.98	6.46
Eicosane	190.94	197.55	-6.61	-3.46
Endrin	117.36	129.93	-12.57	-10.71
Ethanethiol	83.41	87.81	-4.4	-5.28
Ethanol	118.67	124.1	-5.43	-4.58
Ethene	63.22	62.35	0.87	1.38
Ethyl 2-methylbutanoate	121.99	109.07	12.92	10.59
Ethyl 2-methylpentanoate	116.52	115.51	1.01	0.87
Ethyl acetate	101.86	93.88	7.98	7.83
Ethyl acetoacetate	123.8	127.69	-3.89	-3.14
Ethyl acrylate	108	103.24	4.76	4.41
Ethyl benzoate	135.3	127.86	7.44	5.50
Ethyl butanoate	109.88	106.72	3.16	2.88
Ethyl decanoate	147.91	145.33	2.58	1.74
Ethyl ether	87.91	87.41	0.5	0.57
Ethyl formate	98.47	97.53	0.94	0.95
Ethyl glycolate	133.89	154.62	-20.73	-15.48
Ethyl hexanoate	119.6	119.6	0	0.00
Ethyl iodide	87.84	92.1	-4.26	-4.85
Ethyl isobutanoate	105.22	102.67	2.55	2.42
Ethyl levulinate	134.63	134.13	0.5	0.37
Ethyl propanoate	105.58	100.29	5.29	5.01
Ethyl t-amyl ether	104.41	96.8	7.61	7.29
Ethyl t-butyl ether	95.79	90.36	5.43	5.67
Ethyl t-octyl ether	107.97	104.81	3.16	2.93
Ethyl trans-cinnamate	143.65	147.54	-3.89	-2.71
Ethylacetamide	140.26	141.37	-1.11	-0.79
Ethylamine	89.75	89.82	-0.07	-0.08
Ethylbenzene	105.72	103.77	1.95	1.84
Ethylcyclohexane	101.83	107.13	-5.3	-5.20
Ethylcyclopentane	97.94	95.46	2.48	2.53
Ethylenedichloride	99.01	99.88	-0.87	-0.88

Ethylepoxide	100.35	84.82	15.53	15.48
Ethylisopropylketone	102.36	105.95	-3.59	-3.51
Ethylnitrate	100.25	101.69	-1.44	-1.44
Ethyl-n-propylether	93.44	94.15	-0.71	-0.76
Ethyl-t-butylsulfide	101.36	100.79	0.57	0.56
Ethyne	78.99	68.42	10.57	13.38
Fenchone	116.32	109.68	6.64	5.71
Fluorene	124.37	111.59	12.78	10.28
Fluorobenzene	96.66	90.16	6.5	6.72
Formic acid	130.4	125.31	5.09	3.90
Freon 113	88.41	89.08	-0.67	-0.76
Furfural	121.82	110.35	11.47	9.42
Furfuranol	138.72	157.44	-18.72	-13.49
Glycerol formal	133.93	148.99	-15.06	-11.24
Hemimellitene	113.63	116.55	-2.92	-2.57
Heneicosane	201.58	205	-3.42	-1.70
Heptadecane	169.58	175.58	-6	-3.54
Heptane	99.18	101.56	-2.38	-2.40
Heptanedioic acid	204.26	198.49	5.77	2.82
Heptanonitrile	121.42	126.55	-5.13	-4.23
Heptyl bromide	114.98	118.5	-3.52	-3.06
Heptyl butyrate	139.59	138.89	0.7	0.50
Heptylbenzene	135.94	136.24	-0.3	-0.22
Hexachlorobenzene	143.75	136.47	7.28	5.06
Hexacosane	239.64	241.82	-2.18	-0.91
Hexadecylamine	174.91	180.55	-5.64	-3.22
Hexafluorobenzene	87.34	103.74	-16.4	-18.78
Hexamethyleneglycol	187.09	174.95	12.14	6.49
Hexane	92.81	94.45	-1.64	-1.77
Hexanenitrile	114.54	119.77	-5.23	-4.57
Hexapentacontane	471.31	462.92	8.39	1.78
Hexyl bromide	110.78	112.06	-1.28	-1.16
Hexyl hexanoate	150.13	145.33	4.8	3.20
Hexyl t-octyl ether	120.78	130.54	-9.76	-8.08
Hexylbenzene	128.39	129.83	-1.44	-1.12
Icosafluorononane	115.65	115.81	-0.16	-0.14
Iodobenzene	107.8	114.07	-6.27	-5.82
Isoamyl acetate	114.84	109.07	5.77	5.02
Isoamyl isobutyrate	120.74	118.2	2.54	2.10
Isobutyl acetate	89.22	102.67	-13.45	-15.08
Isobutyl chloride	92.34	90.56	1.78	1.93
Isobutyl isobutyrate	119.4	111.76	7.64	6.40
Isobutyl t-butylether	102.36	99.14	3.22	3.15

Isobutylbenzene	111.02	112.9	-1.88	-1.69
Isobutylene	78.08	78.95	-0.87	-1.11
Isobutyronitrile	98.61	103.17	-4.56	-4.62
Isocumene	109.84	110.21	-0.37	-0.34
Isooctane	94.31	93.64	0.67	0.71
Isopentane	81.84	82.94	-1.1	-1.34
Isopentene	84.96	85.36	-0.4	-0.47
Isopentyl alcohol	137.41	139.33	-1.92	-1.40
Isophytol	215.36	226.87	-11.51	-5.34
Isoprene	86.06	88.61	-2.55	-2.96
Isopropyl methyl ether	86.53	89.35	-2.82	-3.26
iso-Propyl nitrite	99.71	97.94	1.77	1.78
Isopropylacetate	102.93	96.83	6.1	5.93
Isopropylamine	92.84	90.12	2.72	2.93
Isopropylcyclopentane	95.09	106.32	-11.23	-11.81
Isoquinoline	119.54	127.75	-8.21	-6.87
Linalool	151.6	180.78	-29.18	-19.25
Linalyl acetate	134.6	144.66	-10.06	-7.47
L-Menthone	114.98	113.73	1.25	1.09
Malathion	189.5	173.97	15.53	8.20
Menthol	164.72	167.77	-3.05	-1.85
Methacrolein	94.78	92.57	2.21	2.33
Methanethiol	81.23	82.78	-1.55	-1.91
Methanol	112.53	122.76	-10.23	-9.09
Methoxybenzene	103.34	115.51	-12.17	-11.78
Methyl acetate	97.7	92.54	5.16	5.28
Methyl acrylate	102.06	101.86	0.2	0.20
Methyl arachidate	220.02	208.62	11.4	5.18
Methyl benzoate	123.23	126.51	-3.28	-2.66
Methyl decanoate	142.85	144.29	-1.44	-1.01
Methyl docosanoate	235.12	221.8	13.32	5.67
Methyl formate	93.04	96.19	-3.15	-3.39
Methyl heneicosanoate	223.58	215.36	8.22	3.68
Methyl heptacosanoate	273.45	253.97	19.48	7.12
Methyl heptadecanoate	193.93	189.3	4.63	2.39
Methyl hexacosanoate	265.37	247.53	17.84	6.72
Methyl hexadecanoate	187.49	182.89	4.6	2.45
Methyl hexanoate	114.64	118.23	-3.59	-3.13
Methyl isobutyl ketone	107.26	105.95	1.31	1.22
Methyl laurate	160.15	157.17	2.98	1.86
Methyl levulinate	131.54	132.79	-1.25	-0.95
Methyl methacrylate	105.25	108.1	-2.85	-2.71
Methyl nonadecanoate	207.04	202.18	4.86	2.35

Methyl nonanoate	135.6	137.55	-1.95	-1.44
Methyl octacosanoate	281.97	260.37	21.6	7.66
Methyl octadecanoate	201.17	195.74	5.43	2.70
Methyl octanoate	130.94	131.11	-0.17	-0.13
Methyl pentacosanoate	257.25	241.09	16.16	6.28
Methyl pentadecanoate	178.23	176.45	1.78	1.00
Methyl propanoate	101.26	98.94	2.32	2.29
Methyl propyl sulfide	98.94	98.27	0.67	0.68
Methyl t-butyl sulfide	91.93	96.09	-4.16	-4.53
Methyl tetracosanoate	245.61	234.68	10.93	4.45
Methyl tetradecanoate	172.97	170.02	2.95	1.71
Methyl t-pentyl ether	97.67	95.46	2.21	2.26
Methyl tridecanoate	163.84	163.61	0.23	0.14
Methyl undecanoate	147.44	150.73	-3.29	-2.23
Methyl valerate	110.95	111.82	-0.87	-0.78
Methylacetamide	133.39	138.82	-5.43	-4.07
Methylamine	86.8	87.61	-0.81	-0.93
Methylbenzene	99.51	97.33	2.18	2.19
Methylchloroacetate	118.3	110.95	7.35	6.21
Methylcyclohexane	95.39	99.68	-4.29	-4.50
Methylcyclopentane	91.7	86.06	5.64	6.15
Methylenanthate	125.54	124.67	0.87	0.69
Methylenecyclohexane	96.6	88.11	8.49	8.79
Methylisocyanate	91.97	85.63	6.34	6.89
Methylnitrite	87.77	93.61	-5.84	-6.65
Methyl-n-propylketone	103.54	103.61	-0.07	-0.07
Methyloxiran	90.89	78.38	12.51	13.76
Methylphosphonic acid dimethyl ester	120.44	121.01	-0.57	-0.47
Methylpropylether	88.45	92.81	-4.36	-4.93
Morpholine	113.03	110.88	2.15	1.90
MTBE	90.29	89.02	1.27	1.41
m-Toluidine	130.34	121.38	8.96	6.87
Mustard gas	127.25	133.72	-6.47	-5.08
Myristyl alcohol	200.97	201.61	-0.64	-0.32
N,N-Dibutylformamide	139.16	146.47	-7.31	-5.25
N,N-Diethylaniline	116.05	124.6	-8.55	-7.37
N,N-Diethylformamide	122.05	120.41	1.64	1.34
N,N-Dimethyl-1-naphthylamine	128.96	148.45	-19.49	-15.11
N,N-Dimethyl-2,3-dimethyl-3-phenyl-2-butanamine	131.81	133.42	-1.61	-1.22
N,N-Dimethyl-3-methyl-3-phenyl-2-butaneamine	121.99	126.92	-4.93	-4.04
N,N-Dimethylaniline	119.27	119.5	-0.23	-0.19
N,N-Dimethylbenzylamine	113.3	118.6	-5.3	-4.68
N,N-Dimethyldodecylamine	135.23	149.25	-14.02	-10.37

N,N-Dimethylformamide	116.15	115.65	0.5	0.43
N,N-Dimethylhexadecylamine	151.23	174.98	-23.75	-15.70
N,N-Dimethyloctylamine	127.28	123.53	3.75	2.95
N,N-Dimethyltetradecylamine	143.45	162.1	-18.65	-13.00
N,N-Dipropylformamide	134.73	133.59	1.14	0.85
Naphthalene	126.11	116.52	9.59	7.60
N-Benzylformamide	152.27	154.79	-2.52	-1.65
n-Butyl acetate	111.62	106.72	4.9	4.39
n-Butyl acrylate	117.59	116.08	1.51	1.28
n-Butyl methyl ether	95.05	99.25	-4.2	-4.42
n-Butylethylether	99.75	100.59	-0.84	-0.84
N-Butylformamide	146.64	133.66	12.98	8.85
n-Butylformate	107.19	110.41	-3.22	-3.00
n-Butylmethylsulfide	103.54	105.05	-1.51	-1.46
n-Decyl alcohol	176.59	175.88	0.71	0.40
n-Decylacetate	148.92	145.33	3.59	2.41
n-Decylamine	143.22	141.61	1.61	1.12
n-Dodecylamine	162.57	154.49	8.08	4.97
Neohexane	85.63	82.84	2.79	3.26
N-Ethylmorpholine	100.02	103.1	-3.08	-3.08
n-Heptaldehyde	118.63	109.14	9.49	8.00
n-Heptylamine	122.25	122.32	-0.07	-0.06
n-Hexanoic acid	151.13	147.34	3.79	2.51
n-Hexylacetate	121.11	119.6	1.51	1.25
n-Hexylamine	109.58	115.88	-6.3	-5.75
Nitrobenzene	115.88	116.25	-0.37	-0.32
Nitrocyclohexane	119.54	129.7	-10.16	-8.50
Nitroethane	109.54	108.67	0.87	0.79
N-Methyl-2,3-dimethyl-3-phenyl-2-butaneamine	153.68	143.79	9.89	6.44
N-Methyl-2-pyrrolidone	143.59	127.99	15.6	10.86
N-Methyl-3-methyl-3-phenyl-2-butaneamine	149.92	137.25	12.67	8.45
N-Methylformamide	122.25	117.93	4.32	3.53
N-Methylmorpholine	102.63	100.55	2.08	2.03
N-Methylpiperidine	92.87	95.46	-2.59	-2.79
N-Methylpropionamide	157.54	145.26	12.28	7.79
N-Methylpyrrolidine	94.45	92.34	2.11	2.23
n-Nonanal	126.51	121.99	4.52	3.57
n-Octanoic acid	169.34	160.22	9.12	5.39
n-Octylacetate	137.65	132.45	5.2	3.78
n-Octylamine	126.55	128.76	-2.21	-1.75
Nonacosane	263.36	263.83	-0.47	-0.18
Nonadecane	183.67	190.11	-6.44	-3.51
Nonafluorocyclopentane	94.28	96.7	-2.42	-2.57

Nonane	113.03	116.42	-3.39	-3.00
Nonanedinitrile	192.49	170.42	22.07	11.47
Nonanedioic acid	208.62	211.71	-3.09	-1.48
Nonylbenzene	147.85	149.45	-1.6	-1.08
Nopinene	99.85	103.3	-3.45	-3.46
n-Propyl acetate	107.19	100.29	6.9	6.44
n-Propyl iodide	97.87	98.54	-0.67	-0.68
n-Propylformate	104.38	103.97	0.41	0.39
n-Propylnitrate	107.19	108.1	-0.91	-0.85
n-Valeraldehyde	102.13	96.29	5.84	5.72
O,O-Diethyl O-2-diethylaminoethyl phosphate	157.87	135.67	22.2	14.06
O,O-Diethyl O-2-diethylaminoethyl thiophosphate	156.8	148.75	8.05	5.13
O,O-Diethyl O-2-dimethylaminoethyl thiophosphate	147.91	143.99	3.92	2.65
O,O-Diethyl O-3-(1-dimethylamino)prop-2-yl thiophosphate	140.23	146.94	-6.71	-4.78
O,O-Diethyl O-3-diethylaminopropyl thiophosphate	158.04	155.16	2.88	1.82
Octacosane	255.71	256.38	-0.67	-0.26
Octadecane	175.55	182.69	-7.14	-4.07
Octamethylcyclotetrasiloxane	135.23	119.3	15.93	11.78
Octane	106.02	108.97	-2.95	-2.78
Octanedioic acid	204.93	204.93	0	0.00
Octatetracontane	413.45	403.76	9.69	2.34
o-Cumenol	172.13	162.84	9.29	5.40
o-Toluidine	125.64	121.38	4.26	3.39
Oxetane	93.07	88.55	4.52	4.86
Oxirane	91.36	75.06	16.3	17.84
Parathion	150.73	152.27	-1.54	-1.02
p-Cymene	115.91	115.91	0	0.00
Pelargonic acid	165.35	166.66	-1.31	-0.79
Pentachlorobenzene	135.74	128.26	7.48	5.51
Pentachloroethane	114.17	110.68	3.49	3.06
Pentacontane	428.68	418.65	10.03	2.34
Pentacosane	232.06	234.41	-2.35	-1.01
Pentadecane	156.4	160.69	-4.29	-2.74
Pentadecylamine	168.98	174.11	-5.13	-3.04
Pentafluorobenzene	101.29	100.82	0.47	0.46
Pentane	85.86	87	-1.14	-1.33
Pentanedinitrile	169.91	144.36	25.55	15.04
Pentanedioic acid	182.79	185.64	-2.85	-1.56
Pentanenitrile	107.6	113.33	-5.73	-5.33
Pentanoic acid	144.69	140.94	3.75	2.59
Pentyl butyrate	122.92	126.01	-3.09	-2.51

Pentyl t-octyl ether	116.65	124.1	-7.45	-6.39
Pentylcyclopentane	116.99	124.94	-7.95	-6.80
Perfluoro-1,4-dithiane S,S'-bis(tetrafluoride)	99.55	104.24	-4.69	-4.71
Perfluoro-2,3-dimethylbutane	94.95	94.25	0.7	0.74
Perfluoro-2-methylbutane	86.9	87.2	-0.3	-0.35
Perfluoro-2-methylpentane	95.22	94.18	1.04	1.09
Perfluoro-3-methylpentane	93.21	94.18	-0.97	-1.04
Perfluorobutylhexane	120.44	118.23	2.21	1.83
Perfluorobutyloctane	131.14	131.11	0.03	0.02
Perfluorobutylpentane	116.22	111.82	4.4	3.79
Perfluorocyclohexane	110.38	94.72	15.66	14.19
Perfluorocyclopentane	86.63	90.69	-4.06	-4.69
Perfluorodiethylsulfur disulfide	94.05	93.34	0.71	0.75
Perfluorodiglyme	102.97	97.47	5.5	5.34
Perfluorodimethylsulfur difluoride	97.8	91.16	6.64	6.79
Perfluoroethylcyclohexane	104.44	108.77	-4.33	-4.15
Perfluoroethylmethylsulfur difluoride	92.07	92.44	-0.37	-0.40
Perfluorohexylhexane	123.9	132.25	-8.35	-6.74
Perfluoromethylcyclohexane	106.36	101.76	4.6	4.32
Perfluoromethylpropylsulfide	88.78	92.47	-3.69	-4.16
Perfluoromethylpropylsulfur difluoride	95.66	99.41	-3.75	-3.92
Perfluoropentane	87.91	87.14	0.77	0.88
Perfluorotetrahydrothiophene	86.06	88.31	-2.25	-2.61
Perfluorotoluene	108.7	111.99	-3.29	-3.03
Perfluoro-trans-decalin	132.89	129.5	3.39	2.55
Perfluorotributylamine	141.47	141.54	-0.07	-0.05
Perfluorotriethylamine	99.31	98.84	0.47	0.47
Perfluorotriglyme	113.1	113.33	-0.23	-0.20
Perfluorotripropylamine	118.83	120.21	-1.38	-1.16
Perylene	181.85	179.04	2.81	1.55
Phenanthrene	137.35	145.46	-8.11	-5.90
Phenethyl acetate	131.18	134.29	-3.11	-2.37
Phenethyl alcohol	158.54	164.51	-5.97	-3.77
Phenethyl butyrate	143.28	147.14	-3.86	-2.69
Phenethyl hexanoate	156.26	159.99	-3.73	-2.39
Phenethyl propionate	137.21	140.7	-3.49	-2.54
Phenol	133.29	144.29	-11	-8.25
Phenylacetic acid	163.84	162.03	1.81	1.10
Phenylisocyanate	108.87	109.81	-0.94	-0.86
Phlorol	145.9	160.49	-14.59	-10.00
Phytane	178.03	181.59	-3.56	-2.00
Phytol	228.27	234.95	-6.68	-2.93
Pimelonitrile	181.69	157.57	24.12	13.28

Pinacolone	102.23	98.44	3.79	3.71
Piperidine	104.85	105.82	-0.97	-0.93
p-Menthadiene	102.13	104.11	-1.98	-1.94
Pristane	171.05	174.14	-3.09	-1.81
Propanal	92.37	83.41	8.96	9.70
Propane	73.12	72.14	0.98	1.34
Propane-1,3-dithiol	122.69	119.07	3.62	2.95
Propanenitrile	97.67	100.49	-2.82	-2.89
Propanoic acid	137.75	128.06	9.69	7.03
Propanone	92.74	90.42	2.32	2.50
Propyl butyrate	119.1	113.16	5.94	4.99
Propyl nitrite	100.15	101.39	-1.24	-1.24
Propyl propanoate	111.82	106.72	5.1	4.56
Propyl tert-amyl ether	114.2	103.24	10.96	9.60
Propyl t-octyl ether	117.06	111.25	5.81	4.96
Propylacetamide	147.07	147.81	-0.74	-0.50
Propylcyclohexane	107.8	114.54	-6.74	-6.25
Propylcyclopentane	103.47	102.9	0.57	0.55
Propylene	68.32	72.72	-4.4	-6.44
Propylenediamine	121.42	117.26	4.16	3.43
Propyne	73.96	77.31	-3.35	-4.53
p-Toluidine	127.42	121.38	6.04	4.74
Pulegone	122.39	121.48	0.91	0.74
Pyrene	149.42	150.43	-1.01	-0.68
Pyridine	105.18	98.81	6.37	6.06
Pyrrolidine	104.71	102.36	2.35	2.24
Pyruvaldehyde	99.58	104.38	-4.8	-4.82
Quinoline	120.64	113.63	7.01	5.81
s-Butylacetate	104.81	103.27	1.54	1.47
s-Butylamine	101.19	96.56	4.63	4.58
s-Butylbenzene	115.18	112.9	2.28	1.98
s-Butylnitrite	103.37	104.34	-0.97	-0.94
Sebaconitrile	197.28	176.86	20.42	10.35
s-Phenethyl alcohol	160.25	166.43	-6.18	-3.86
s-trans-1,3-Butadiene	79.26	82.41	-3.15	-3.97
Styrene	107.8	107.03	0.77	0.71
Suberonitrile	187.22	163.98	23.24	12.41
Sulcatone	122.32	129.2	-6.88	-5.62
t-Butylamine	96.26	96.63	-0.37	-0.38
t-Butylbenzene	110.58	105.35	5.23	4.73
t-Butylhydroperoxide	119.84	109.98	9.86	8.23
t-Butylmercaptan	91.4	91.7	-0.3	-0.33
t-Butylnitrite	102.16	97.6	4.56	4.46

Tetracontane	351.97	344.96	7.01	1.99
Tetracosane	223.81	226.97	-3.16	-1.41
Tetradecane	146.5	153.28	-6.78	-4.63
Tetradecylbenzene	186.55	181.96	4.59	2.46
Tetraethylmethane	104.61	105.15	-0.54	-0.52
Tetrahydrofuran	95.35	83.41	11.94	12.52
Tetrahydropyran	108.4	96.03	12.37	11.41
Tetramethylene glycol	165.42	162.1	3.32	2.01
Tetramethylethene	94.35	95.19	-0.84	-0.89
Tetrapentacontane	457.96	448.06	9.9	2.16
Tetratetracontane	384.14	374.34	9.8	2.55
Thietane	97.84	93.44	4.4	4.50
Thiolane	99.51	100.12	-0.61	-0.61
Thiophene	99.18	94.21	4.97	5.01
trans,trans-2,4-Hexadienyl acetate	124.94	133.29	-8.35	-6.68
trans-1,2-Dibromocycloheptane	104.11	117.32	-13.21	-12.69
trans-1,2-Dibromocyclohexane	102.16	104.71	-2.55	-2.50
trans-1,2-Dibromocyclooctane	107.06	129.9	-22.84	-21.33
trans-1,2-Dibromocyclopentane	105.79	101.56	4.23	4.00
trans-1,2-Dichloroethylene	87.64	88.45	-0.81	-0.92
trans-1,2-Dimethylcyclohexane	95.52	103.04	-7.52	-7.87
trans-1,2-Dimethylcyclopentane	95.49	98.91	-3.42	-3.58
trans-1,3-Dimethylcyclohexane	99.75	103.04	-3.29	-3.30
trans-1,3-Dimethylcyclopentane	95.19	98.91	-3.72	-3.91
trans-1,3-Pentadiene	88.45	92.4	-3.95	-4.47
trans-1,4-Dimethylcyclohexane	96.63	103.04	-6.41	-6.63
trans-2-Butene	78.42	83.08	-4.66	-5.94
trans-2-Heptene	98.61	102.36	-3.75	-3.80
trans-2-Hexene	94.78	95.92	-1.14	-1.20
trans-2-Methylcyclohexanol	158.91	156.3	2.61	1.64
trans-2-Octene	103.61	109.14	-5.53	-5.34
trans-2-Pentene	86.37	89.49	-3.12	-3.61
trans-2-Phenylcyclopropylamine	129.26	127.32	1.94	1.50
trans-3-Heptene	96.83	102.36	-5.53	-5.71
trans-3-Hexene	92.77	95.92	-3.15	-3.40
trans-3-Octene	103.37	109.14	-5.77	-5.58
trans-4,4-Dimethyl-2-pentene	93.95	91.1	2.85	3.03
trans-4,6-Dimethyl-1,3-dioxane	98.88	97.9	0.98	0.99
trans-4-Octene	112.86	109.14	3.72	3.30
trans-Crotononitrile	102.67	113.77	-11.1	-10.81
Triacontane	271.47	271.24	0.23	0.08
Tributyl phosphate	161.06	147.54	13.52	8.39
Tributylamine	123.9	128.29	-4.39	-3.54

Trichloroethylene	95.99	109.31	-13.32	-13.88
Trichlorotoluene	126.85	122.96	3.89	3.07
Tridecane	142.34	145.83	-3.49	-2.45
Tridecylbenzene	168.74	175.18	-6.44	-3.82
Triethyl phosphate	114.88	108.97	5.91	5.14
Triethylamine	97.17	89.72	7.45	7.67
Trimethyl phosphate	103.04	105.25	-2.21	-2.14
Trimethylamine	82.84	82.07	0.77	0.93
Trimethylethylene	86.87	88.98	-2.11	-2.43
Tripropylamine	103.17	109.01	-5.84	-5.66
Triptane	92	86.2	5.8	6.30
Undecane	126.38	131.31	-4.93	-3.90
Undecanedioic acid	222.37	224.55	-2.18	-0.98
Undecylbenzene	162.64	162.3	0.34	0.21
Vinyl acetate	109.07	93.54	15.53	14.24
Vinylidenechloride	87.2	96.26	-9.06	-10.39
Zingiberene	150.16	130.97	19.19	12.78
