# Supplementary material for: Calculation of the Vapour Pressure of Organic Molecules by Means of a Group-Additivity Method and Their Resultant Gibbs Free Energy and Entropy of Vaporization at 298.15 K
Source: Molecules. 2021 Feb 17;26(4):1045. doi: 10.3390/molecules26041045 (PMC7922249; doi:10.3390/molecules26041045)
Supplement: Supplementary file 1 [file molecules-26-01045-s001.zip › molecules-1089923-SM-proofed/Table 1.pdf]

| Atom Type | Neighbours | Meaning |
|---|---|---|
| O(prim) | HC | Primary alcohol |
| O(sec) | HC | Secondary alcohol |
| O(tert) | HC | Tertiary alcohol |
| (COH)n | n>1 | Molecule contains more than 1 OH group |
| (COOH)n | n>1 | Molecule contains more than 1 carboxylic acid group |
| Endocyclic bonds | No of single bonds | Number of single bonds in cyclic ring |
| Bridgehead atoms | No of bonds | Number of bridgehead C or N (e.g. camphor, DABCO) |
