# Supplementary material for: Calculation of the Vapour Pressure of Organic Molecules by Means of a Group-Additivity Method and Their Resultant Gibbs Free Energy and Entropy of Vaporization at 298.15 K
Source: Molecules. 2021 Feb 17;26(4):1045. doi: 10.3390/molecules26041045 (PMC7922249; doi:10.3390/molecules26041045)
Supplement: Supplementary file 1 [file molecules-26-01045-s001.zip › molecules-1089923-SM-proofed/Table 2.pdf]

Atom Type / Neighours	C sp3 / H3C	C sp3 / HC3	C sp3 / HC2O	C sp3 / H2C2	O(sec) / HC	Endocyclic Single bds	Const	Checksum
**Contribution**	0.60	−1.28	−2.65	−0.47	0.72	0.31	4.71	
**n**	1	1	1	4	1	6	1	
**n x Contrib.**	0.6	−1.28	−2.65	−1.88	0.72	1.86	4.71	**2.08**
