# Supplementary material for: Calculation of the Vapour Pressure of Organic Molecules by Means of a Group-Additivity Method and Their Resultant Gibbs Free Energy and Entropy of Vaporization at 298.15 K
Source: Molecules. 2021 Feb 17;26(4):1045. doi: 10.3390/molecules26041045 (PMC7922249; doi:10.3390/molecules26041045)
Supplement: Supplementary file 1 [file molecules-26-01045-s001.zip › molecules-1089923-SM-proofed/Table 3.pdf]

Entry	Atom Type	Neighbours	Contribution	Occurrences	Molecules
1	Const		4.71	2036	2036
2	B	HN2	-1.17	6	2
3	B	BN2	-1.6	2	1
4	B	BO2	-1.71	4	2
5	B	C2N	-0.35	1	1
6	B	C2O	-0.44	1	1
7	B	C2S	-0.44	1	1
8	B	CO2	-1.56	1	1
9	B	O3	-1.57	6	6
10	B	S3	-3.17	1	1
11	C sp3	H3B	0	7	4
12	C sp3	H3C	0.6	2211	1077
13	C sp3	H3N	-1.07	113	62
14	C sp3	H3N(+)	-1.64	1	1
15	C sp3	H3O	-0.95	152	116
16	C sp3	H3S	-0.52	23	17
17	C sp3	H3P	-1.22	8	7
18	C sp3	H3Si	-0.42	87	16
19	C sp3	H2C2	-0.47	4196	831
20	C sp3	H2CN	-2.07	240	138
21	C sp3	H2CN(+)	-2.01	5	5
22	C sp3	H2CO	-1.82	460	314
23	C sp3	H2CP	-2.3	5	3
24	C sp3	H2CS	-1.6	79	54
25	C sp3	H2CF	0.39	15	15
26	C sp3	H2CCl	-0.48	59	48
27	C sp3	H2CBr	-0.76	22	20
28	C sp3	H2CJ	-1.23	11	11
29	C sp3	H2CSi	-1.58	11	6
30	C sp3	H2N2	-11.73	1	1
31	C sp3	H2NO	-3.82	2	2
32	C sp3	H2NS	-1.24	3	3
33	C sp3	H2O2	-3.81	6	6
34	C sp3	H2OF	-1.22	3	3
35	C sp3	H2OCl	-2.1	2	2
36	C sp3	H2S2	-2.59	3	3
37	C sp3	HC3	-1.28	342	231
38	C sp3	HC2N	-2.87	35	28
39	C sp3	HC2N(+)	-2.87	3	3
40	C sp3	HC2O	-2.65	115	95
41	C sp3	HC2S	-2.36	11	8
42	C sp3	HC2F	-0.6	10	9
43	C sp3	HC2Cl	-1.22	31	15
44	C sp3	HC2Br	-1.59	16	12
45	C sp3	HC2J	-1.96	1	1
46	C sp3	HCN2	-2.07	2	1
47	C sp3	HCNO	-5.99	1	1
48	C sp3	HCNS	-2.48	1	1

49	C sp3	HCO2	-3.65	7	7
50	C sp3	HCOBr	-4.78	1	1
51	C sp3	HCF2	0.37	31	27
52	C sp3	HCFCl	-0.01	7	7
53	C sp3	HCCl2	-0.94	12	11
54	C sp3	HCClBr	-0.76	1	1
55	C sp3	HCBr2	-1.93	3	2
56	C sp3	HOF2	-1.09	6	6
57	C sp3	C4	-2.19	98	87
58	C sp3	C3N	-3.6	11	11
59	C sp3	C3N(+)	-3.57	2	2
60	C sp3	C3O	-3.46	36	35
61	C sp3	C3S	-3.21	6	6
62	C sp3	C3Si	-3.37	3	2
63	C sp3	C3Cl	-2.84	6	3
64	C sp3	C3Br	-2.2	2	2
65	C sp3	C3F	-1.39	13	10
66	C sp3	C2O2	-5.46	4	2
67	C sp3	C2OF	-2.8	5	5
68	C sp3	C2F2	-0.37	184	71
69	C sp3	C2FCl	-0.8	1	1
70	C sp3	C2Cl2	0	3	3
71	C sp3	CNF2	-2.03	12	5
72	C sp3	CNF2(+)	-0.37	1	1
73	C sp3	CNCl2	-0.4	1	1
74	C sp3	COF2	-1.69	49	39
75	C sp3	CSF2	-1.15	24	12
76	C sp3	CF3	0.67	152	107
77	C sp3	CF2Cl	0.3	8	7
78	C sp3	CF2Br	-0.07	5	4
79	C sp3	CFCl2	-0.37	5	4
80	C sp3	CFClBr	-0.73	1	1
81	C sp3	CCl3	-0.98	15	14
82	C sp3	CCl2Br	0	1	1
83	C sp3	NF3	-1.09	5	3
84	C sp3	OF3	-0.36	13	10
85	C sp3	O2F2	-2.67	1	1
86	C sp3	S2F2	-1.83	4	2
87	C sp3	SF3	-0.01	10	7
88	C sp3	SCl3	-7.92	1	1
89	C sp3	PF3	-0.08	20	8
90	C sp2	H2=C	0.67	127	113
91	C sp2	HC=C	-0.38	272	175
92	C sp2	HC=N	-1.49	7	7
93	C sp2	HC=O	-0.47	27	27
94	C sp2	H=CN	-1.84	19	12
95	C sp2	H=CO	-0.79	5	5
96	C sp2	H=CS	-0.79	8	6
97	C sp2	H=CP	-1.03	3	1

98	C sp2	H=CF	0.68	3	3
99	C sp2	H=CCl	-0.15	13	11
100	C sp2	H=CBr	-0.56	5	3
101	C sp2	H=CJ	-1.2	2	1
102	C sp2	HN=N	-1.89	11	9
103	C sp2	HN=O	-2.47	9	8
104	C sp2	HO=O	-1.25	8	8
105	C sp2	C2=C	-1.25	79	67
106	C sp2	C2=N	-3.09	2	2
107	C sp2	C=CN	-2.26	2	2
108	C sp2	C2=O	-1.27	56	53
109	C sp2	C=CO	-1.5	6	6
110	C sp2	C=CP	-3.09	1	1
111	C sp2	C=CS	-1.78	6	5
112	C sp2	C=CF	-0.25	3	3
113	C sp2	C=CCl	-1.24	18	13
114	C sp2	CN=N	-4.13	2	2
115	C sp2	CN=O	-3.17	35	32
116	C sp2	C=NS	-1.47	2	1
117	C sp2	CO=O	-2.33	222	184
118	C sp2	C=OCl	-0.54	4	4
119	C sp2	C=OBr	-1.1	1	1
120	C sp2	C=OJ	-1.67	1	1
121	C sp2	=CF2	0.95	7	6
122	C sp2	=CFCl	0.14	1	1
123	C sp2	=CFBr	-0.25	1	1
124	C sp2	=CCl2	-0.53	10	8
125	C sp2	=CBr2	0.65	1	1
126	C sp2	N2=N	-4.7	1	1
127	C sp2	N2=O	-5.19	5	5
128	C sp2	N=NS	-1.58	1	1
129	C sp2	N2=S	0.14	2	1
130	C sp2	NO=O	-4.55	15	13
131	C sp2	N=OS	-0.64	7	7
132	C sp2	=NOS	-0.26	1	1
133	C sp2	NS=S	1.24	1	1
134	C sp2	O2=O	-3.59	4	4
135	C aromatic	H:C2	-0.2	3662	751
136	C aromatic	H:C:N	-0.41	34	21
137	C aromatic	H:N2	0.48	2	2
138	C aromatic	:C3	-1.06	260	85
139	C aromatic	C:C2	-1.06	929	508
140	C aromatic	C:C:N	-1.21	15	13
141	C aromatic	:C2N	-2.3	40	38
142	C aromatic	:C2N(+)	-2.59	33	29
143	C aromatic	:C2:N	-1.42	4	3
144	C aromatic	:C2O	-2	381	195
145	C aromatic	:C2P	-4.07	1	1
146	C aromatic	:C2S	-1.69	8	6

147	C aromatic	:C2F	-0.1	63	26
148	C aromatic	:C2Cl	-0.84	1630	386
149	C aromatic	:C2Br	-1.13	166	58
150	C aromatic	:C2J	-1.57	10	9
151	C aromatic	:C2Si	0.89	1	1
152	C aromatic	C:N2	-1.39	2	2
153	C aromatic	:C:NO	-1.85	6	6
154	C aromatic	:C:NCl	-1.33	5	5
155	C aromatic	N:N2	-2.72	17	10
156	C aromatic	:N2O	-0.96	2	2
157	C aromatic	:N2S	1.89	3	3
158	C aromatic	:N2Cl	-1.38	3	3
159	C sp	H#C	0.81	14	13
160	C sp	C#C	-0.49	22	17
161	C sp	=C2	-0.51	3	3
162	C sp	C#N	-0.61	34	27
163	C sp	=N=O	0.75	3	3
164	C sp	=N=S	1.19	1	1
165	N sp3	HB2	0.45	3	2
166	N sp3	H2C	1.45	62	47
167	N sp3	H2C(pi)	0.15	18	18
168	N sp3	H2N	-0.52	3	3
169	N sp3	HC2	2.36	26	26
170	N sp3	HC2(pi)	0.56	35	26
171	N sp3	HC2(2pi)	0.44	14	10
172	N sp3	HCN	0.7	3	2
173	N sp3	HCN(pi)	-0.36	1	1
174	N sp3	HCN(2pi)	0.38	1	1
175	N sp3	HCP(pi)	-4.25	1	1
176	N sp3	HCS(pi)	5.56	1	1
177	N sp3	B2C	1.1	3	2
178	N sp3	BC2	2.13	5	2
179	N sp3	C3	3.52	49	45
180	N sp3	C3(pi)	2.95	27	26
181	N sp3	C3(2pi)	3.52	11	11
182	N sp3	C3(3pi)	3.4	3	3
183	N sp3	C2N(pi)	0.11	4	4
184	N sp3	C2N(2pi)	3.37	8	8
185	N sp3	C2N(3pi)	2.89	1	1
186	N sp3	C2O	3.47	1	1
187	N sp3	C2S	2.57	3	3
188	N sp3	C2S(pi)	3.96	3	2
189	N sp3	C2S(2pi)	7.1	1	1
190	N sp3	C2P	2.07	7	4
191	N sp3	C2F(pi)	4.38	1	1
192	N sp3	CF2	0.61	1	1
193	N sp3	CSi2	1.18	2	2
194	N sp3	SF2	0.07	1	1
195	N sp2	C=C	0.39	16	15

196	N sp2	C=N	-3.19	1	1
197	N sp2	C=N(+)	0.96	7	7
198	N sp2	=CN	-0.04	10	9
199	N sp2	=CO	0.68	4	3
200	N sp2	=CS	-0.39	1	1
201	N sp2	N=N	0	1	1
202	N sp2	N=O	0	4	4
203	N sp2	=NP(+)	-0.39	1	1
204	N sp2	O=O	1.58	6	6
205	N aromatic	:C2	-0.06	61	39
206	N(+) sp2	CO=O(-)	0.34	45	41
207	N(+) sp2	O2=O(-)	0.54	50	26
208	N(+) sp	=N2(-)	0	8	8
209	O(prim)	HC	0.44	95	78
210	O(sec)	HC	0.72	48	47
211	O(tert)	HC	0.74	11	11
212	O	HC(pi)	0.04	102	90
213	O	HN(pi)	-1.29	1	1
214	O	HO	-1.16	4	3
215	O	BC	1.39	26	8
216	O	BP	0.16	3	2
217	O	C2	2.38	150	132
218	O	C2(pi)	2.3	228	191
219	O	C2(2pi)	1.49	151	130
220	O	CN	0	1	1
221	O	CN(pi)	0	6	6
222	O	CN(2pi)	0.26	3	2
223	O	CN(+)(pi)	0	50	26
224	O	CO	1.03	8	3
225	O	CO(pi)	1.59	3	2
226	O	CS	1.25	6	4
227	O	CS(pi)	1.44	2	2
228	O	CP	0.06	95	44
229	O	CP(pi)	-0.29	14	12
230	O	CSi	0.65	7	2
231	O	OS	-0.67	3	2
232	O	S2	-1.14	5	3
233	O	Si2	-0.3	22	7
234	P3	C3	0	1	1
235	P3	HC2	2.57	1	1
236	P3	C2N	1.59	2	2
237	P3	C2O	0	3	2
238	P3	C2S	-0.09	5	4
239	P3	CN2	-0.35	1	1
240	P3	CS2	-0.94	1	1
241	P4	HO2=O	-0.55	1	1
242	P4	C3=S	0.19	1	1
243	P4	CO2=O	0.62	4	4
244	P4	CO2=S	3.03	1	1

245	P4	CO=OS	0.38	2	2
246	P4	COS=S	-0.5	1	1
247	P4	N3=O	-0.83	1	1
248	P4	NO=OS	-0.06	1	1
249	P4	N=OF2	0	1	1
250	P4	O3=O	0.23	9	9
251	P4	O3=S	0.22	13	13
252	P4	O2=OS	-0.36	1	1
253	P4	O=OS2	-1.76	1	1
254	P4	O2S=S	-0.58	12	11
255	S2	HC	0.83	29	23
256	S2	HC(pi)	0.28	1	1
257	S2	HS	-0.26	2	1
258	S2	HP	0.06	1	1
259	S2	BC	0.52	4	2
260	S2	C2	1.07	30	28
261	S2	C2(pi)	-1.97	14	13
262	S2	C2(2pi)	1.52	9	9
263	S2	CN	0	1	1
264	S2	CN(2pi)	-2.3	1	1
265	S2	CS	0.05	8	4
266	S2	CP	-0.07	22	19
267	S2	CP(pi)	0	1	1
268	S2	N2	-1.45	2	2
269	S2	NCl	-0.43	1	1
270	S2	P2	-0.7	1	1
271	S2	Si2	0.33	3	3
272	S4	C2=O	-0.96	4	4
273	S4	C2=O2	1.6	2	2
274	S4	C2O2	-2.15	1	1
275	S4	C2F2	0.41	5	5
276	S4	CO=O2	2.16	1	1
277	S4	CN=O2	-2.13	1	1
278	S4	NO=O2	-2.51	1	1
279	S4	N=O2Cl	0	1	1
280	S4	O2=O	-0.56	1	1
281	S4	O2=O2	-0.94	1	1
282	S4	O=O2F	0.12	4	4
283	S6	C2F4	0.72	5	3
284	S6	O2F4	-0.78	1	1
285	S6	OF5	1.08	7	5
286	Si	H3C	1.72	4	4
287	Si	H3N	0	4	2
288	Si	H3S	-0.3	2	1
289	Si	H3Si	-0.53	2	1
290	Si	H2C2	1.78	2	2
291	Si	H2Si2	0	2	1
292	Si	HC2O	0.78	2	1
293	Si	HC2S	0.11	2	1

294	Si	HC2J	0.23	1	1
295	Si	HCCl2	0.47	1	1
296	Si	HO3	0.21	1	1
297	Si	C4	1.97	2	2
298	Si	C3O	1.08	6	3
299	Si	C3S	0.19	2	1
300	Si	C3Cl	1.05	1	1
301	Si	C3Si	-0.79	2	1
302	Si	C2O2	-0.18	18	5
303	Si	C2F2	1.69	1	1
304	Si	C2Cl2	0.41	1	1
305	Si	CF3	0	1	1
306	Si	CCl3	0.06	1	1
307	Si	O4	-0.16	1	1
308	(COH)n	n>1	-0.74	23	22
309	(COOH)n	n>1	-1.73	12	12
310	Endocyclic bonds	No of single bds	0.31	1072	193
311	Bridgehead atoms	No of atoms	0.23	80	27
312	Angle60		0.19	42	14
313	Angle90		0.17	72	21
314	Angle102		0.11	323	110
**A**	**Based on**	**Valid groups**	**171**		**2036**
**B**	**Goodness of fit**	**R^2**	**0.9946**		**1908**
**C**	**Deviation**	**Average**	**0.18**		**1908**
**D**	**Deviation**	**Standard**	**0.24**		**1908**
**E**	**K-fold cv**	**K**	**10**		**1842**
**F**	**Goodness of fit**	**Q^2**	**0.9938**		**1842**
**G**	**Deviation**	**Average (cv)**	**0.2**		**1842**
**H**	**Deviation**	**Standard (cv)**	**0.26**		**1842**
